# Photoredox ketone catalysis for the direct C–H imidation and acyloxylation of arenes†
†Electronic supplementary information (ESI) available: Experimental procedures and characterization data for all relevant compounds. CCDC 1544882. For ESI and crystallographic data in CIF or other electronic format see DOI: 10.1039/c7sc01700f



**DOI:** 10.1039/c7sc01700f

**Published:** 2017-06-05

**Authors:** Chandra Bhushan Tripathi, Tsuyoshi Ohtani, Michael T. Corbett, Takashi Ooi

**Affiliations:** a Institute of Transformative Bio-Molecules (WPI-ITbM) , Department of Molecular and Macromolecular Chemistry , Graduate School of Engineering , Nagoya University , Nagoya 464-8601 , Japan . Email: tooi@chembio.nagoya-u.ac.jp; b CREST , Japan Science and Technology Agency (JST) , Nagoya University , Nagoya 464-8601 , Japan

## Abstract

The photoexcited aryl ketone-catalyzed C–H imidation of arenes and heteroarenes is reported. Using 3,6-dimethoxy-9*H*-thioxanthen-9-one as a catalyst in combination with a bench-stable imidating reagent, C–N bond formation proceeds with high efficiency and a broad substrate scope. A key part of this method is that the thioxanthone catalyst acts as an excited-state reductant, thus establishing an oxidative quenching cycle for radical aromatic substitution. The synthetic potential of this photoexcited ketone catalysis is further demonstrated by application to the direct C–H acyloxylation of arenes.

## Abstract 2

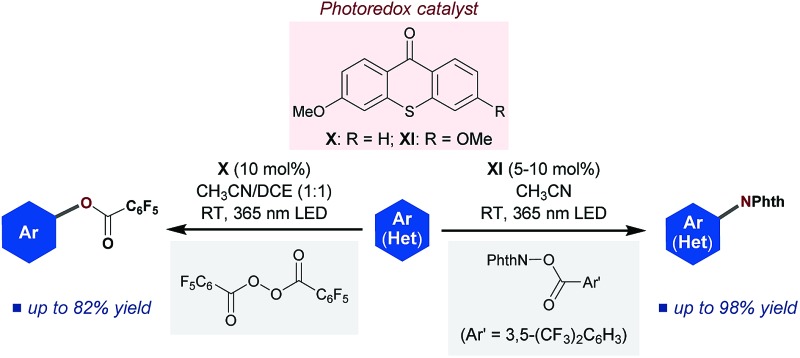
Using a tuned yet simple catalyst, the photoexcited ketone-catalyzed C–H imidation and acyloxylation of arenes through an oxidative quenching cycle has been developed.

## Introduction

**Scheme 1:**
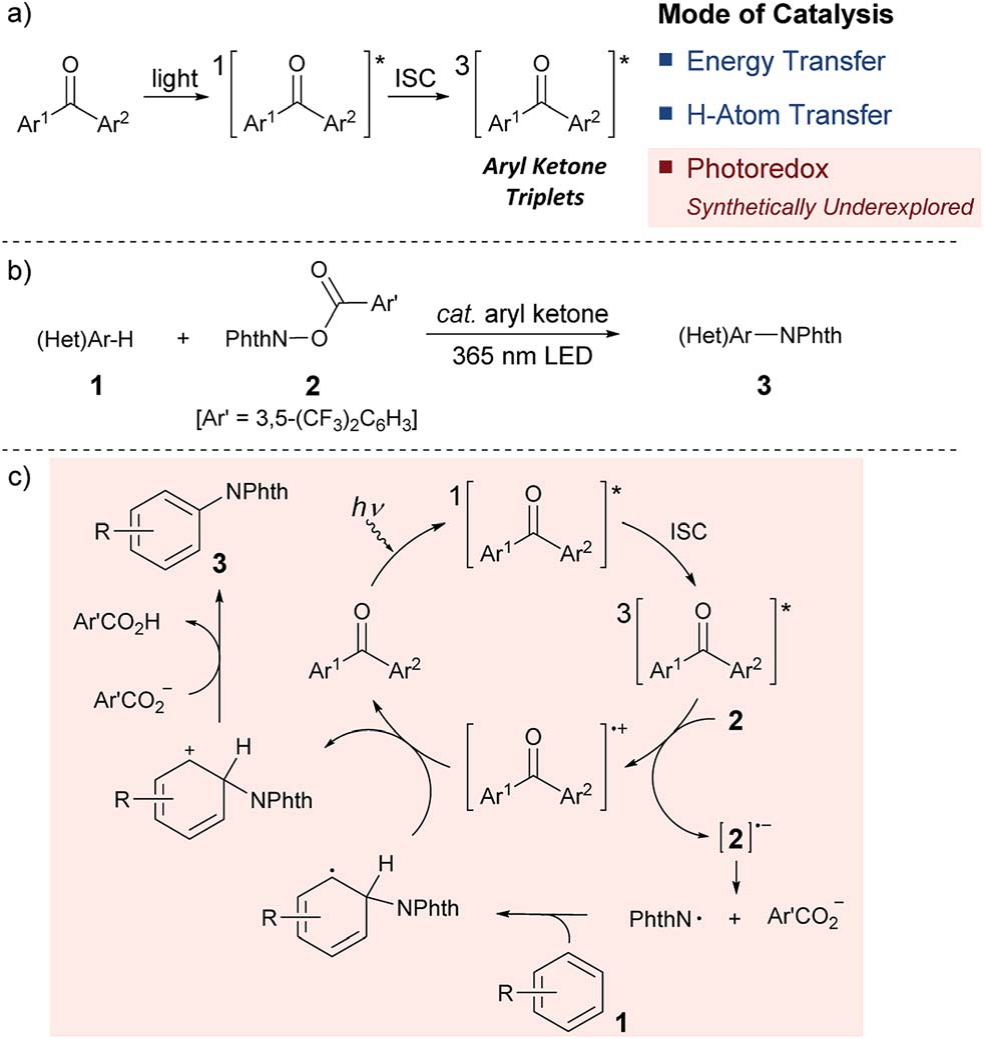
(a) Modes of photoexcited aryl ketone catalysis. (b) Photoexcited aryl ketone-catalyzed C–H imidation of arenes (Phth = phthaloyl). (c) Proposed catalytic cycle.

Since its inception, the photochemistry of carbonyl compounds, especially ketones, has been studied extensively, and the electronically excited state of ketones is known to undergo different types of bond scission and reformation depending on the reaction conditions.^1^ In addition to their own structural reorganizations and transformations, a series of aryl ketones, such as benzophenone and its derivatives, act as effective photosensitizers.^2^ Upon exposure to light, they are excited to a singlet state, and subsequent rapid transition to a triplet state through intersystem crossing (ISC) proceeds almost quantitatively. Owing to their relatively long lifetimes, aryl ketone triplets have long been appreciated for their capability to facilitate photochemical reactions. However, their actual usage as catalysts in selective organic synthesis has been rather limited (Scheme 1a). In particular, while the ability of photoexcited aryl ketones to mediate triplet energy transfer (EnT)^3^ and hydrogen atom transfer (HAT)^4^ has been exploited in several reaction systems, the utility of their photoinduced electron transfer (PET) reactivity in catalysis remains largely underexplored.^5^ This is rather intriguing as the simple aryl ketones offer a unique opportunity to tune the redox properties for a given transformation through elaboration and ready modification of the primary ketone frameworks. In this context, and in consideration of the prevailing mode of photocatalysis with the currently available organic chromophores,^6^ we became interested in exploring the potential of aryl ketones as photoredox catalysts, specifically as excited-state reductants, in synthetically valuable bond-forming reactions. As an initial step, we disclose herein the efficient catalysis of appropriately modified thioxanthones under photoirradiation for the direct C–H imidation of arenes and heteroarenes (Scheme 1b). The applicability of thioxanthone catalysis to the C–H acyloxylation of arenes is also demonstrated.

Aromatic and heteroaromatic amines constitute the core structural components of a wide array of functional organic molecules.^7^ Accordingly, the development of reliable methods for the assembly of arylamines has been a subject of central importance in synthetic chemistry, and direct arene C–H aminations have emerged as powerful means for this pursuit.^8,9^ Among the various strategies developed to date, the photocatalytic system reported by Sanford is unique,^9*a*^ wherein a key nitrogen-based radical was generated from *N*-acyloxyphthalimide through one-electron reduction by an iridium-centred photosensitizer under visible light irradiation. This mechanistic proposal, in addition to the inherent synthetic value of C–N bond formation in its own right, inspired us to choose this class of C–H amination as a testing ground for photoredox ketone catalysis. We envisaged that if the triplet excited state of an aryl ketone could donate an electron to the aminating reagent, the corresponding anion radical would form with concomitant generation of a ketone cation radical. The anion radical then fragments to generate a requisite phthalimidyl radical that participates in the radical aromatic substitution process (Scheme 1c). At this stage, we recognized that aryl ketones are generally poor reductants, and distinguishing between the energy and electron transfer pathways may also be challenging.^10^ Nevertheless, we reasoned that the use of aryl ketones with appropriate structural features in combination with an electronically modulated *N*-acyloxyphthalimide would enable the establishment of an oxidative quenching cycle, thereby allowing the photoredox ketone-catalyzed C–H imidation of arenes.

## Results and discussion

At the outset of our investigation to assess the validity of this hypothesis, we selected benzotrifluoride (**1a**) as a model substrate with the expectation that if a sufficient level of reactivity was attained with this generally less reactive arene, we could demonstrate the advantages of our approach through the reaction development (Table 1). An initial experiment was thus conducted by stirring a mixture of 3,5-bis(trifluoromethyl)phenylacyloxyphthalimide (**2**),^11^ **1a** (10 equiv.) and a catalytic amount of benzophenone (**I**) (5 mol%) in acetonitrile (CH_3_CN) under 365 nm LED light irradiation (1500 W m^–2^) at ambient temperature for 15 h. However, ^1^H-NMR analysis of the crude material showed very low conversion. Subsequent attempts with benzophenone derivatives, such as **II** and **III**, revealed that an electron-rich catalyst exhibited better efficacy, while changing the ketone skeleton to fluorenone (**IV**) turned out to be ineffective. To further evaluate the relationship between the structure and activity of aryl ketone catalysts, we examined the reaction in the presence of thioxanthone (**V**), which is known to have a long-lived triplet excited state, and observed an improved reactivity profile.^12^ On the other hand, the use of structurally related xanthone (**VI**) and 10-benzylacridin-9(10*H*)-one (**VII**) resulted in lower conversions. We next pursued the structural modification of the thioxanthone framework by introducing an electron-donating group to the 3-position, which had a notable yet beneficial impact on the catalytic activity. Under the influence of 3-methyl and 3-dimethylamino-substituted **VIII** and **IX** as catalysts, C–N bond formation occurred with significantly higher efficiency and the imidated product **3a** was isolated in good yields. Interestingly, 3-methoxy derivative **X** exerted even higher catalytic activity. These observations led us to prepare 3,6-dimethoxy-9*H*-thioxanthen-9-one (**XI**) and we found that it delivered a critical improvement in the reactivity. Eventually, by increasing the loading of **XI** to 10 mol%, this imidation of the electron-deficient arene **1a** proceeded smoothly to afford **3a** in near quantitative yield (98%). Meanwhile, we screened other representative *N*-acyloxyphthalimides with different leaving abilities of the carboxylate anion as imidating agents; however, **2** remained optimal.^13^ It is also worth adding that the C–H imidation relied on the intensity of 365 nm LED, as the reaction under irradiation with an intensity of 500 W m^–2^ exhibited lower conversion (75%), whereas full conversion was observed with an intensity of 1000 W m^–2^ and 1500 W m^–2^.^13^

**Table 1:** Optimization for the photoexcited ketone-catalyzed C–H imidation of arenes^*a*^ ^,^ ^*b*^

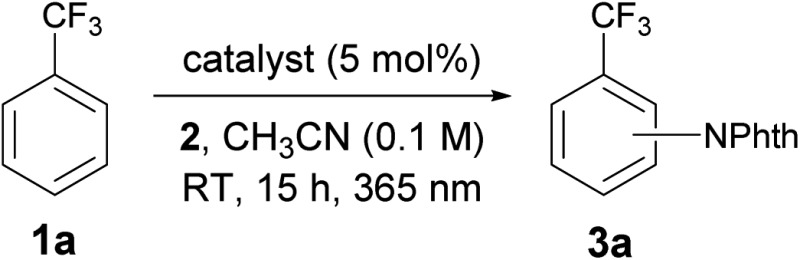
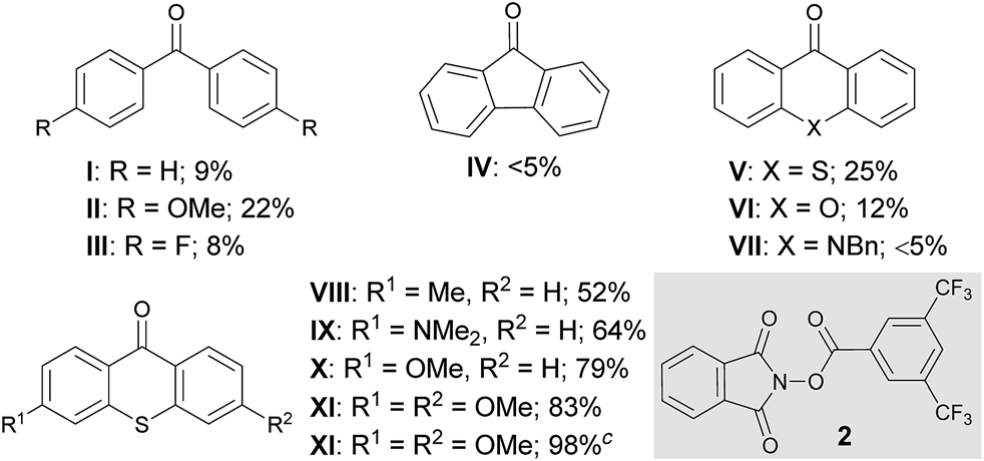

^*a*^Reactions were carried out on a 0.1 mmol scale with **2** (1.0 equiv.) and **1a** (10.0 equiv.) under light irradiation (1500 W m^–2^).

^*b*^The conversions were determined through ^1^H-NMR analysis of the crude reaction mixture.

^*c*^10 mol% of **XI** was used and isolated yields are indicated (*o*/*m*/*p* = >0.1 : 2.6 : 1.0).

The optimal catalyst and reaction conditions were applied to probe the scope of this photoexcited ketone-catalyzed C–H imidation protocol (Table 2). As summarized in Table 2, a broad range of arenes and heteroarenes underwent imidations in good to high yields under the catalysis of **XI**. The reactivity profile depended on the electronic nature of the arenes. The present system accommodated simple arenes, heteroarenes and electron-rich arenes, and the use of 5 mol% of **XI** was sufficient for smooth reactions. The imidations of electron-deficient arenes were generally challenging; however, a satisfactory level of reactivity could be attained by increasing the loading of **XI** to 10 mol%. It should be noted that the observed site selectivity is analogous to that anticipated for a radical aromatic substitution reaction.^9b,14^ Moreover, reactions with the arene as the limiting reagent also appeared feasible under slightly modified conditions, as exemplified by the direct installation of the phthalimide functionality onto caffeine.

**Table 2:** Substrate scope of the photoexcited ketone-catalyzed C–H imidation of arenes^*a*^ ^,^ ^*b*^

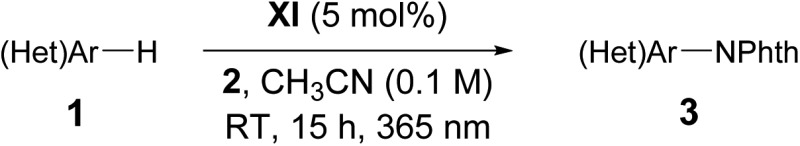
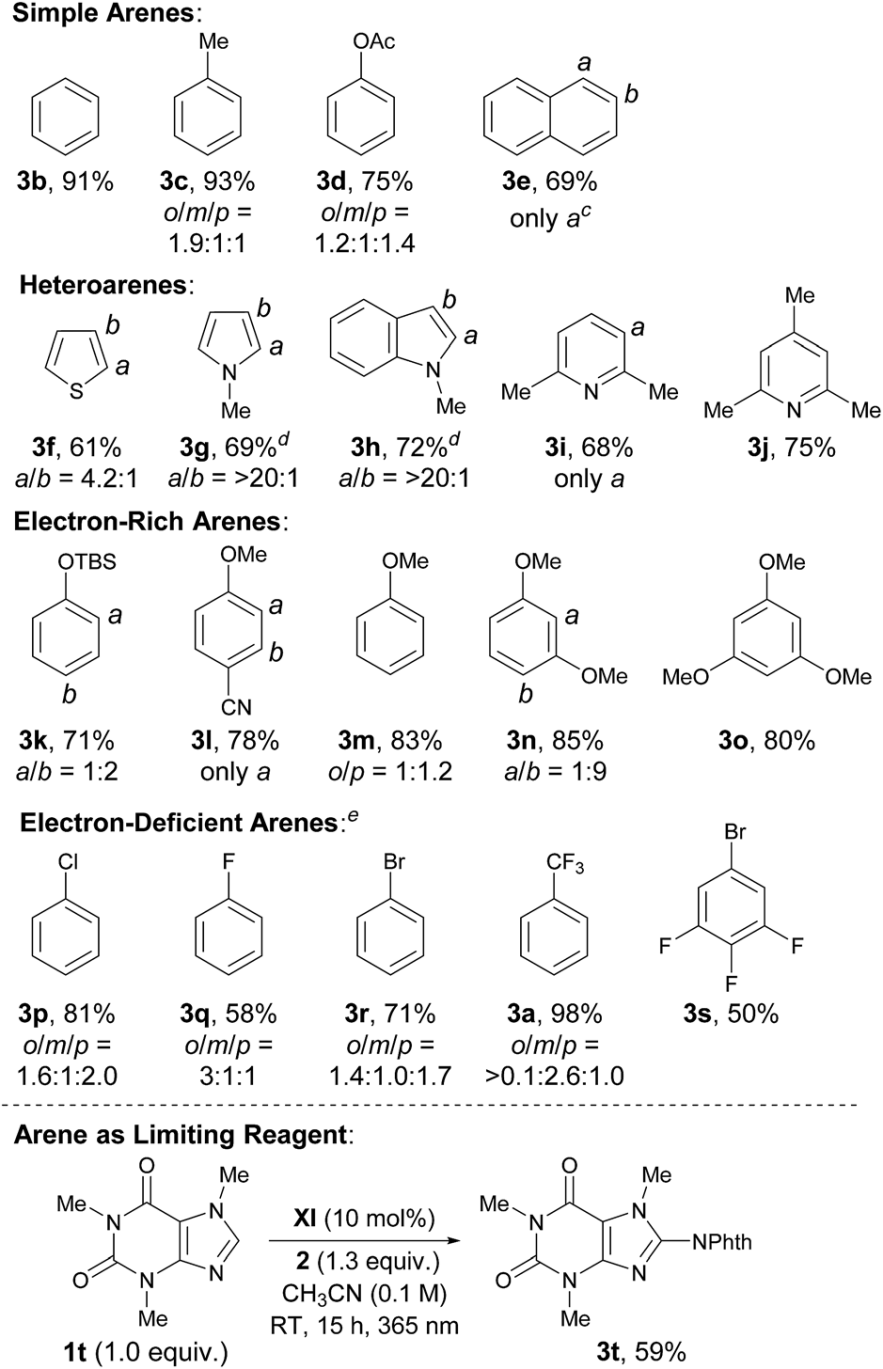

^*a*^Reactions were carried out on a 0.1 mmol scale with **2** (1.0 equiv.) and arenes (10.0 equiv.) under light irradiation (1500 W m^–2^).

^*b*^Isolated yields of **3** are indicated.

^*c*^Crude products contained two isomers in a ratio of *a*/*b* = 7 : 1.

^*d*^2.0 equiv. of K_2_CO_3_ were used.

^*e*^Performed with 10 mol% of **XI** (TBS = *tert*-butyldimethylsilyl).

**Scheme 2:**
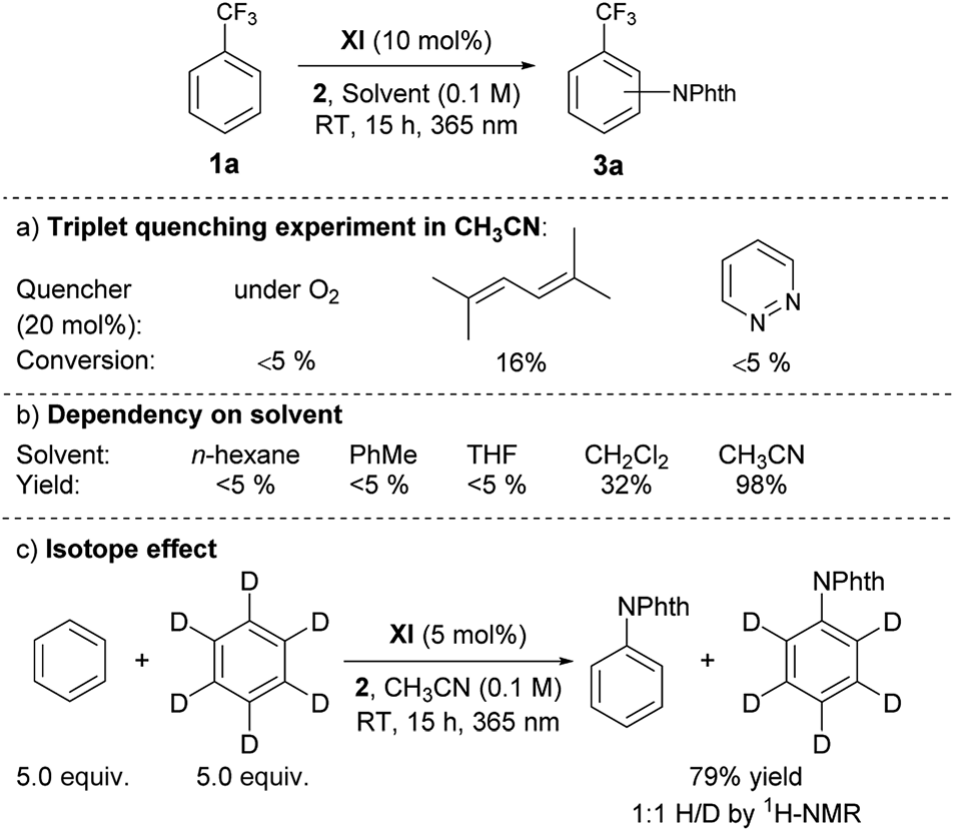
(a) Influence of triplet quenchers. (b) Solvent dependency. (c) Isotope effect.

Having grasped the general applicability, we then studied the reaction mechanism with the primary objective of distinguishing the presumed electron transfer (ET) pathway from the possible alternative that involves energy transfer (EnT) from the triplet excited state of **XI** to the imidating agent **2**, followed by homolytic cleavage of the N–O bond. This mechanistic study was initiated by measuring the UV-visible spectra of the representative catalysts, **V** and **XI**, and **2** in CH_3_CN, which revealed that only the catalyst has an absorption in the range of 365 nm. We then performed a reaction with benzotrifluoride (**1a**) under the optimized conditions but with light irradiation at fixed intervals, and observed that the reaction proceeded only when irradiated.^13^ We also detected a low quantum yield (*Φ* = 0.036) for the imidation.^13^ These results not only confirmed that photoexcitation was essential but also suggested the limited intervention of a radical chain process.^15^ Another useful piece of information to ascertain the involvement of the triplet excited state of the ketone catalyst was that the reaction was significantly suppressed by triplet quenchers (O_2_, pyridazine and 2,5-dimethylhexa-2,4-diene)^16*a*^ (Scheme 2a).

**Scheme 3:**
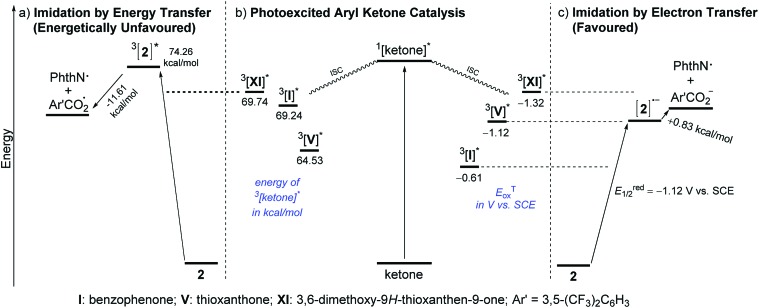
(a–c) Representative diagram for the comparison of C–H imidation through energy or electron transfer pathways (values determined through phosphorescence spectra, cyclic voltammetry measurements and UB3RYP/6-311+G(d,p) calculations).^18^

Unlike reactions that proceed through EnT, this imidation reaction depended heavily on the solvent, and substantial product formation was observed only in CH_3_CN, a general characteristic of reactions involving ET processes (Scheme 2b).^16^ Furthermore, the Δ*G*_et_ for **XI** was calculated to be –4.61 kcal mol^–1^ by the Rehm–Weller equation, indicating the feasibility of ET from **XI** to **2**.^13^ At the same time, however, we recognized that these observations were still circumstantial, and thus, more compelling evidence was obtained by determining the triplet excited state energies and redox potentials of ketone catalysts **XI**, **I** and **V**, and the imidating agent **2**, by measuring phosphorescence spectra and using cyclic voltammetry as well as theoretical calculations (Scheme 3).^13^

As illustrated in Scheme 3a and b, the triplet excited state of **2**, ^3^[**2**]*, has an energy of 74.26 kcal mol^–1^, whereas those of the ketones, ^3^[ketone]*, lie at much lower energy levels (^3^[**XI**]* = 69.74 kcal mol^–1^, ^3^[**I**]* = 69.24 kcal mol^–1^ and ^3^[**V**]* = 64.53 kcal mol^–1^). This energy gap between ^3^[ketone]* and ^3^[**2**]* (4.5 kcal mol^–1^ even for **XI**) is significant enough to preclude the possibility of the EnT pathway. In addition, even if the triplet excited state energy is a critical element for the ketone catalyst to be able to mediate the imidation, the nearly equal energy levels of ^3^[**XI**]* and ^3^[**I**]* could not rationalize the experimentally observed considerable difference in reactivity between **I** and **XI**. On the other hand, comparison of the triplet excited state oxidation potentials (*E*Tox) of the ketones and the reduction potential (*E*red1/2) of **2** strongly supported the operation of the ET pathway. Among **XI**, **V** and **I** with *E*Tox values of –1.32 V, –1.12 V and –0.61 V, respectively, *versus* SCE, **XI** should be the most competent electron donor to **2** (*E*red1/2 = –1.12 V *vs.* SCE), followed by **V** and **I** (Scheme 3b and c), which is in accordance with the experimental results.

The outcomes of these investigations prove that the photoexcited ketone-catalyzed direct arene imidation proceeds through an ET pathway, meaning that aryl ketones with suitable electronic properties, such as the optimal catalyst **XI**, act as excited-state reductants to establish an oxidative quenching cycle for radical aromatic substitution, as we initially postulated (Scheme 1b). The catalytic cycle commences with photoexcitation and subsequent ISC to afford ^3^[ketone]*. The ketone triplet with an appropriate oxidation potential donates an electron to the imidating agent, **2**, to form a ketone cation radical ([ketone]˙^+^)^17^ and an anion radical of **2** ([**2**]˙^–^) that undergoes fragmentation to generate a phthalimidyl radical (PhthN˙) and a 3,5-bis(trifluoromethyl)benzoate anion. The PhthN˙ adds to the arene to bring forth a neutral radical species that can be oxidized by [ketone]˙^+^ to provide a Wheland intermediate and regenerate the ketone catalyst. Deprotonation of the Wheland intermediate by the 3,5-bis(trifluoromethyl)benzoate anion yields **3** and the corresponding carboxylic acid.^19^ It is noteworthy that the absence of the kinetic isotope effect rules out the possibility of C–H abstraction as a rate-determining step (Scheme 2c).

After establishing the C–H imidation of arenes, we decided to further explore the synthetic potential of photoexcited ketone catalysis and found it to be applicable to the C–H acyloxylation of simple arenes.^20^ For instance, light irradiation (325 W m^–2^) over a mixture of pentafluorobenzoyl peroxide (**4**) and benzene (10 equiv.) in CH_3_CN/DCE (1 : 1) in the presence of 3-methoxy-9*H*-thioxanthen-9-one (**X**) (10 mol%) at room temperature for 15 h resulted in the formation of the acyloxylated product **5a** in good yield (Table 3).^21^ Other selected examples listed in Table 3 show the tolerance of the present system to the electronic property of arenes.

**Table 3:** Photoexcited ketone-catalyzed C–H acyloxylation of arenes^*a*^ ^,^ ^*b*^

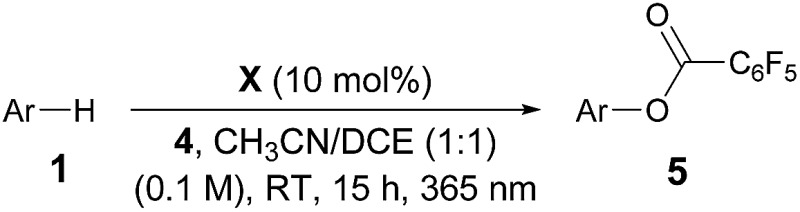
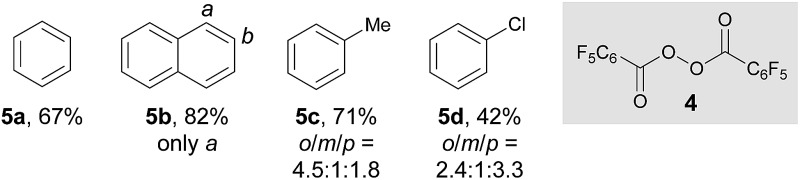

^*a*^Reactions were carried out on a 0.2 mmol scale with **4** (1.0 equiv.) and arenes (10.0 equiv.) under light irradiation (325 W m^–2^).

^*b*^Isolated yields of **5** are indicated.

## Conclusions

In conclusion, we have developed a photoexcited ketone-catalyzed C–H imidation of arenes. Under simple and mild conditions, direct C–N bond formation proceeds efficiently with a broad range of arenes and heteroarenes. A distinct feature of this novel photocatalytic system is that the thioxanthone-derived catalyst behaves as an excited-state one-electron reductant and thus establishes an oxidative quenching cycle, as verified unambiguously through mechanistic investigations based on experimental and theoretical approaches. The utility of photoexcited ketone catalysis has also been demonstrated by application to the direct C–H acyloxylation of arenes. We believe that the present study indicates the possibility of designing and structurally manipulating simple aryl ketones to explore their potential utility as photoredox catalysts, which would be valuable in the development of unique synthetic transformations under organic photoredox catalysis.
