# Supplementary material for: Photoredox ketone catalysis for the direct C–H imidation and acyloxylation of arenes
Source: Chem Sci. 2017 Jun 5;8(8):5622–7. doi: 10.1039/c7sc01700f (PMC5620997; doi:10.1039/c7sc01700f)
Supplement: Supplementary file 1 [file SC-008-C7SC01700F-s001.pdf]

Electronic Supplementary Material (ESI) for Chemical Science.
This journal is © The Royal Society of Chemistry 2017

# Photoredox Ketone Catalysis for Direct C-H Imidation and Acyloxylation of Arenes

Chandra Bhushan Tripathi,[†] Tsuyoshi Ohtani,[‡] Michael T. Corbett[†] and Takashi Ooi*[,†,‡,‡]

[†]*Institute of Transformative Bio-Molecules (WPI-ITbM),* [‡] *Department of Molecular and Macromolecular Chemistry, Graduate School of Engineering, Nagoya University, Nagoya 464-8601, Japan*

‡*CREST, Japan Science and Technology Agency (JST), Nagoya University, Nagoya 464-8601, Japan*

tooi@chembio.nagoya-u.ac.jp

## SUPPORTING INFORMATION

A. General Information S-2

B. Synthesis of Ketone Catalysts S-2

C. Synthesis and Characterization of Substituted *N*-Acyloxyphthalimides S-2

D. Reaction Conditions Optimization for the Ketone-Catalyzed C-H Imidation S-3

E. Photoexcited Ketone-Catalyzed C-H Imidation of Arenes S-6

F. Calculation of $\Delta G_{et}$ by Rehm-Weller Equation S-13

G. UV-Visible Absorption Spectroscopy S-14

H. Phosphorescence Spectroscopy S-15

I. Electrochemical Studies S-16

J. Determination of Excited State Potentials S-18

K. Quantum Yield Measurement S-19

L. Crystallographic Structure Determination of **3l** S-22

M. Computational Studies S-23

N. Synthesis and Characterization of Aryl-Acylperoxide S-36

O. Reaction Conditions Optimization for the Ketone-Catalyzed C-H Acyloxylation S-36

P. Photoexcited Ketone-Catalyzed C-H Acyloxylation of Arenes S-38

Q. Copies of NMR Spectra S-40

## A. General Information:

Infrared (FT-IR) spectra were recorded on a Shimadzu IRAffinity-1 spectrometer ($\nu_{max}$ in $cm^{-1}$). $^{1}$H-NMR spectra were recorded on a JEOL JNM-ECS600 (600 MHz), JNM-ECA500II (500 MHz) and JNM-ECS400 (400 MHz) spectrometers. Chemical shifts are reported in ppm from the solvent resonance as the internal standard ($CDCl_3$: δ 7.26 and DMSO-$d_6$: δ 2.50). Data are reported as follows: chemical shift, multiplicity (s = singlet, d = doublet, dd = doublet of doublet, t = triplet, q = quartet, br = broad, m = multiplet), coupling constants (Hz) and integration. $^{13}$C-NMR spectra were recorded on JEOL JNM-ECS600 (150 MHz), JNM-ECA500II (125 MHz) and JNM-ECS400 (100 MHz) spectrometers with complete proton decoupling. Chemical shifts are reported in ppm from tetramethylsilane with the solvent resonance as the internal standard ($CDCl_3$: δ 77.16 and DMSO-$d_6$: δ 39.52). Mass spectra were measured with a Thermo Fisher Scientific Exactive (ESI). Analytical thin layer chromatography (TLC) was performed on Merck precoated TLC plates (silica gel 60 GF254, 0.25 mm). Flash column chromatography was performed on silica gel 60 (spherical, 40-50 µm; Kanto Chemical Co., Inc.), silica gel 60 (Merck 1.09385.9929, 230–400 mesh). Unless otherwise noted all reactions were carried out with distilled and dried solvents under an atmosphere of argon. All work up and purifications were carried out with reagent grade solvents in air.

## B. Synthesis of Ketone Catalysts:

The commercially available ketone derivatives were used as such without any purification. Catalysts **VII-XI** were prepared by following the literature procedure.[1, 2] The spectral data are in agreement with those reported in the literature.

## C. Synthesis and Characterization of Substituted *N*-Acyloxyphthalimides:

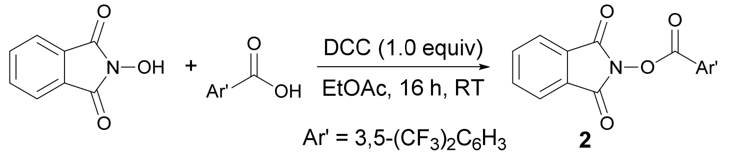


In a 200 mL round bottom flask equipped with magnetic stir bar, *N*-hydroxyphthalimide (10.0 mmol, 1.63 g, 1.0 equiv) and dicyclohexylcarbodiimide (10.0 mmol, 2.06 g 1.0 equiv) were placed. Ethyl acetate (50 mL) and 3,5-bis(trifluoromethyl)benzoic acid (10 mmol, 2.58 g, 1.0 equiv) were successively added. The resulting mixture was stirred for 16 h and the formation of a white precipitate was observed. The solid was removed by vacuum filtration and dried in vacuo to afford the crude product. Purification by column chromatography over silica gel using (Hexane/EtOAc = 3:1 as eluent) gave **2** as a white solid. Recrystallization from hot Hexane/EtOAc yielded **2** as colorless crystals (2.50

1. T. Stopka, L. Marzo, M. Zurro, S. Janich, E.-U. Würthwein, C. G. Daniliuc, J. Alemán and O. G. Mancheño, *Angew. Chem. Int. Ed.* 2015, **54**, 5049.
2. S. Khan, P. L. Bernad, V. A. Korshun, E. M. Southern and M. S. Shchepinov, *Synlett* 2005, 2453.

g, 6.2 mmol, 62% yield). **FT-IR (thin film)**: 3068, 1782, 1745. **$^1$H-NMR ($CDCl_3$, 500 MHz)**: δ 8.65 (s, 2H), 8.21 (s, 1H), 7.97 (dd, $J$ = 5.5, 3.0 Hz, 2H), 7.86 (dd, $J$ = 5.5, 3.0 Hz, 2H). **$^{13}$C-NMR ($CDCl_3$, 125 MHz)**: δ 161.6, 160.8, 135.2, 133.0 (q, $J$ = 29 Hz), 128.9, 128.3 (m), 127.9, 124.5, 122.7 (q, $J$ = 228 Hz). **$^{19}$F-NMR (471 MHz, $CDCl_3$):** δ –62.934. **HRMS (ESI+)**: Calcd. for $C_{18}H_{11}O_5NF_6Na$ ([M + Na + MeOH]$^+$): 458.0424, Found: 458.0429.

The spectral data of remaining imidation reagents are in agreement with those reported in the literature.[3]

**D. Reaction Conditions Optimization for the Ketone-Catalyzed C-H Imidation:**

***Effect of Concentration***

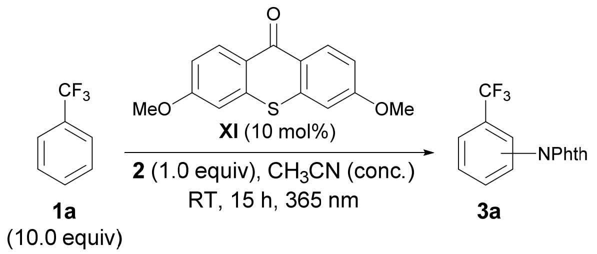


| | | | |
|---|---|---|---|
| conc.: | 0.1 M | 0.05 M | 0.5 M |
| conv.: | >99% | >99% | 52% |

The optimization of substrate concentration revealed that 0.1 M and 0.05 M were equally efficient.

***Effect of Imidating Agent***

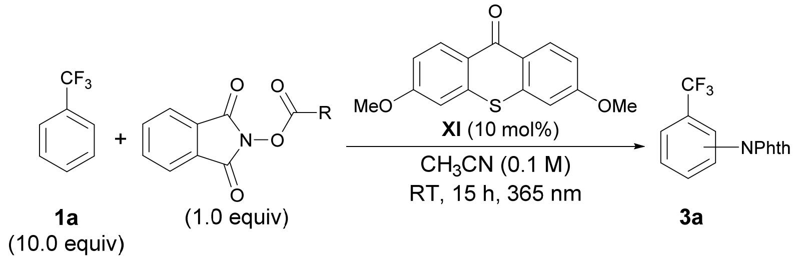


| | | | | | |
|---|---|---|---|---|---|
| R: | 4-OMe$C_6H_4$ | R = Ph | R = 4-$CF_3C_6H_4$ | R = $CF_3$ | R = 3,5-$(CF_3)_2C_6H_3$ (**2**) |
| conv. (yield): | 61% (<10%) | 40% (<10%) | 57% (55%) | >99% (64%) | >99% (98%) |

We envisioned that the imidating agents with electron-withdrawing groups might be appropriate for this direct C-H imidation of arenes. With this premise in mind, several imidating agents with different leaving ability of carboxylate anion were synthesized and screened. As shown above, the results were obtained on the expected lines. The reactions of **1a** with imidating agents possessing 4-(trifluoromethyl)phenyl and trifluoromethyl groups, which were utilized by Sanford and co-workers,[3] afforded the product **3a** in good yields. At this point, we prepared 3,5-bis(trifluoromethyl)phenyl-substituted **2** and its use for the imidation of **1a** led to the production of **3a**

3. L. J. Allen, P. J. Cabrera, M. Lee and M. S. Sanford, *J. Am. Chem. Soc.* 2014, **136**, 5607.

in 98% yield. It is important to note that unlike other imidating agents, **2** can be stored at room temperature for months without any deterioration.

***ON/OFF Experiment***

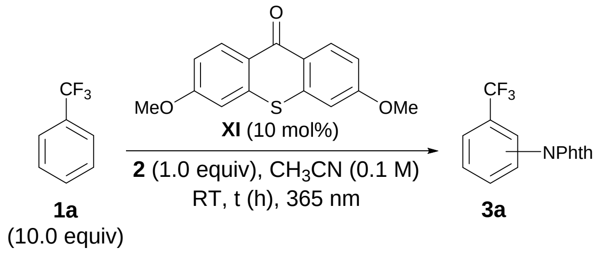


| Conditions | Yield (%) |
|---|---|
| ON, 2 h | 61 |
| ON, 2 h → OFF, 2 h | 61 |
| ON, 2 h → OFF, 2 h → ON, 2 h | 83 |

The reaction mixture was irradiated at fixed intervals. As evident from the data listed in the above Table, the reaction proceeded only in the presence of light. This result showed that light is essential for the reaction and it also provided a preliminary support for the absence of a radical chain process, which was later confirmed by a low quantum yield of 0.036 (see page S-19).

***Effect of Light Intensity***

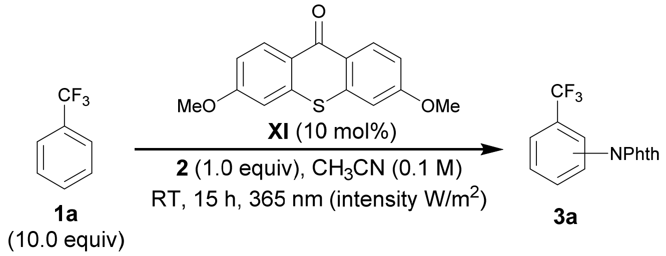


| intensity W/m$^2$: | 500 | 1000 | 1500 |
|---|---|---|---|
| conv.: | 75% conversion | >99% conversion | >99% conversion (98% yield) |

The reaction relied upon the intensity of the 365 nm LED. As summarized in the above scheme, when the reaction mixture was irradiated at 500W/m$^2$, low conversion (75%) was observed, whereas the imidation proceeded smoothly with the intensity of 1000 W/m$^2$ and 1500 W/m$^2$. Unless otherwise stated, all reactions in this study are carried out with the intensity of 1500 W/m$^2$.

## ***Recovery of the Acid***

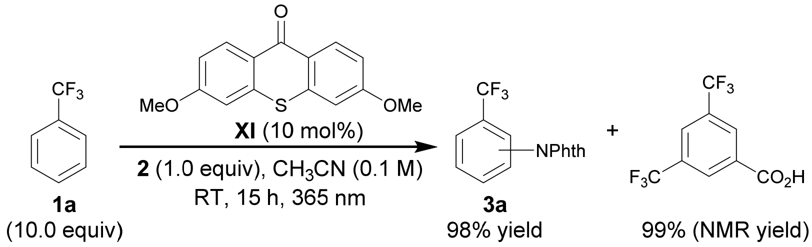


In the C-H imidation of **1a** using **2**, 3,5-bis(trifluoromethyl)benzoic acid was recovered in 99% NMR yield. This experiment corroborates the generation of a 3,5-bis(trifluoromethyl)benzoate anion in the catalytic cycle rather than the corresponding radical that would be susceptible towards decarboxylation to eventually form 1,3-bis(trifluoromethyl)benzene.

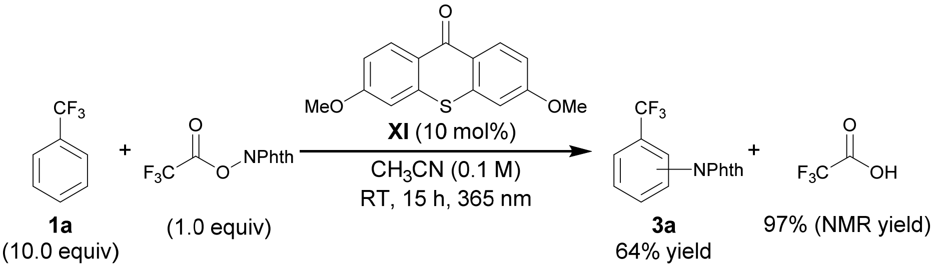


The point described above was further confirmed by carrying out the C-H imidation of **1a** with the imidating agent introduced by Sanford,[3] which furnished **3a** in 64% yield and trifluoroacetic acid (TFA) in 97% NMR yield. The generation of TFA in an amount larger than that of **3a** can be accounted for by the hydrolysis of the moisture sensitive imidating agent. This outcome supports the generation of trifluoroacetate anion in the catalytic cycle rather than trifluoroacetate radical that is known to undergo rapid decarboxylation to generate the trifluoromethyl radical.[4]

## ***Triplet Lifetime of Ketones (τ) and Catalytic Activity***

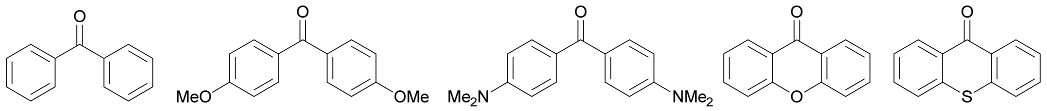

| | | | | | |
|---|---|---|---|---|---|
| Triplet lifetime (μs): | 6.9 | 47 | 28 | 0.09 | 95 |
| Triplet energy (kJ/mol): | 287 | 293 | 275 | 310 | 265 |
| Conversion (%): | 9 | 22 | 70 | <5 | 25 |

The screening of ketones with different $\tau$ values in the imidation of **1a** with **2** (triplet energy: 310.7 kJ/mol) under the conditions described for Table 1 (main text) showed no direct correlation between $\tau$ and catalytic activity. As seen in the above scheme, 4,4'-dimethoxybenzophenone was significantly less active than more electron-rich Michler's ketone that has shorter triplet lifetime and lower triplet

4. J. W. Beatty, J. J. Douglas, K. P. Cole and C. R. J. Stephenson, *Nat. Commun.*, 2015, **6**, 7919

energy.[5] Because of the difficulty associated with further modifications of Michler's ketone and presumed instability of the resulting even more electron-rich ketones under the reaction conditions, we decided to proceed toward structural tuning of thioxanthone framework for reactivity enhancement.

**E. Photoexcited Ketone-Catalyzed C-H Imidation of Arenes:**

**General procedure for the ketone-catalyzed C-H imidation of arenes:**

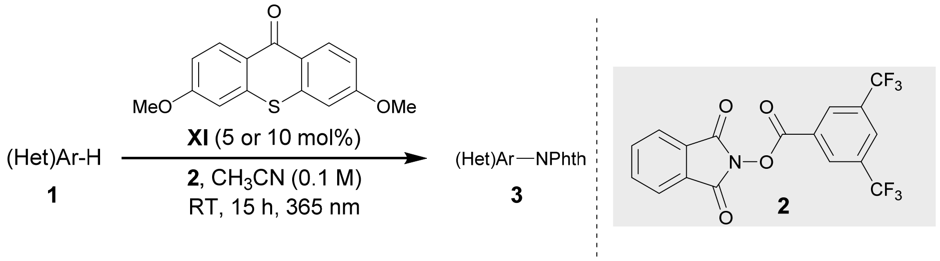


In a flame and vacuum dried reaction tube, **2** (40.3 mg, 0.1 mmol, 1.0 equiv) and **XI** were taken. The reaction flask was degassed in *vacuo* and backfilled with argon. To the mixture $CH_3CN$ (1.0 mL) and arene (1.0 mmol, 10.0 equiv) were added. The resulting solution was stirred at 25 °C under 365 nm irradiation (1500 $W/m^2$) for 15 h. The reaction was quenched by adding a saturated aqueous solution of $NaHCO_3$ (5 mL). The aqueous phase was extracted with $CH_2Cl_2$ (3 × 10 mL) and the combined organic phase was dried over anhydrous $Na_2SO_4$, concentrated under reduced pressure. Purification of the residue by column chromatography over silica gel afforded **3**.

**3a**: Following the general procedure described above with **XI** (2.7 mg, 0.01 mmol, 0.1 equiv) and benzotrifluoride (0.122 mL, 1.0 mmol, 10.0 equiv) as the arene source, **3a** was obtained as a white solid (28.5 mg, 0.098 mmol, 98% yield, *o*/*m*/*p* = >0.1:2.6:1.0). The isomer ratios were determined through $^{19}$F-NMR. The isolated product was an inseparable mixture of isomers. All peaks observed in the $^{1}$H-NMR, $^{13}$C-NMR and $^{19}$F-NMR were assigned. **$^{1}$H-NMR (500 MHz, $CDCl_3$)**: δ 7.99-7.98 (m, 2.1H), 7.84-7.82 (m, 2.1H), 7.78-7.77 (m, 1.25H), 7.70-7.64 (m, 3H). **$^{13}$C-NMR (125 MHz, $CDCl_3$)**: δ 167.0, 134.9, 132.5, 131.7, 129.8, 126.6, 126.4, 124.8, 124.1, 123.5. **$^{19}$F-NMR (471 MHz, $CDCl_3$):** δ –61.3, –62.5, –62.6. The spectral data are consistent with those reported in the literature.[3]

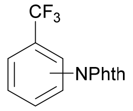


**3b**: Following the general procedure described above with **XI** (1.35 mg, 0.005 mmol, 0.05 equiv) and benzene (0.093 mL, 1.0 mmol, 10.0 equiv) as the arene source, **3b** was obtained as a white solid (20.2 mg, 0.091 mmol, 91% yield). **$^{1}$H-NMR (600 MHz, $CDCl_3$)**: δ 7.97 (dd, *J* = 5.4, 3.6 Hz, 2H), 7.80 (dd, *J* = 5.4, 3.6 Hz, 2H), 7.51 (t, *J* = 7.8 Hz, 2H), 7.44 (d, *J* = 7.8 Hz, 2H),

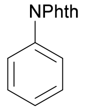


5. a) M. W. Wolf, R. E. Brown and L. A. Singer, *J. Am. Chem. Soc.*, 1977, **99**, 526; b) A. Garner and F. Wilkinson, *J. Chem. Soc., Faraday Trans. 2*, 1976, **72**, 1010; c) "Handbook of Photochemistry": Third Edition, Taylor and Francis, 2005, pp.86-156.

7.42 (t, $J$ = 7.8 Hz, 1H). **$^{13}$C-NMR (150 MHz, $CDCl_3$)**: δ 167.4, 134.6, 131.9, 131.8, 129.3, 128.3, 126.7, 123.9. The spectral data are consistent with those reported in the literature.[3]

**3c**: Following the general procedure described above with **XI** (1.35 mg, 0.005 mmol, 0.05 equiv) and toluene (0.10 mL, 1.0 mmol, 10.0 equiv) as the arene source, **3c** was obtained as a white solid (22.0 mg, 0.093 mmol, 93% yield, *o*/*m*/*p* = 1.9:1:1). The isomer ratios were determined through $^{1}$H-NMR. The isolated product was an inseparable mixture of isomers. All peaks observed in the $^{1}$H-NMR and $^{13}$C-NMR were assigned. **$^{1}$H-NMR (600 MHz, $CDCl_3$)**: δ 7.97-7.90 (m, 8H), 7.78-7.86 (m, 8H), 7.39-7.33 (m, 5H), 7.32-7.27 (m, 7H), 7.22-7.16 (m, 5H), 2.40 (s, 3H), 2.39 (s, 3H), 2.19 (s, 5.9H). **$^{13}$C-NMR (150 MHz, $CDCl_3$)**: δ 167.51, 167.45, 167.43, 138.3, 136.6, 134.4, 132.1, 131.91, 131.88, 131.2, 130.6, 129.8, 129.5, 129.1, 129.04, 129.01, 128.8, 127.3, 126.9, 126.5, 123.8, 123.78, 123.75, 21.47, 21.3, 18.1. The spectral data are in agreement with those reported in the literature.[3]

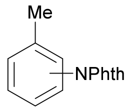


**3d**: Following the general procedure described above with **XI** (1.35 mg, 0.005 mmol, 0.05 equiv) and phenyl acetate (0.13 mL, 1.0 mmol, 10.0 equiv) as the arene source, **3d** was obtained as a white solid (21.0 mg, 0.075 mmol, 75% yield, *o*/*m*/*p* = 1.2:1:1.4). The isomer ratios were determined through $^{1}$H-NMR. The isolated product was an inseparable mixture of isomers. All peaks observed in the $^{1}$H-NMR and $^{13}$C-NMR were assigned. **$^{1}$H-NMR (600 MHz, $CDCl_3$)**: δ 8.01-7.92 (m, 7H), 7.85-7.76 (m, 7H), 7.52-7.45 (m, 5H), 7.41-7.35 (m, 4H), 7.32-7.29 (m, 1H), 7.25 (t, $J$ = 6 Hz, 4H), 7.16 (d, $J$ = 8.4 Hz, 1H), 2.32 (s, 4.4H), 2.31 (s, 3H), 2.13 (s, 3.6H). **$^{13}$C-NMR (150 MHz, $CDCl_3$)**: δ 169.2, 169.1, 168.2, 167.2, 166.9, 166.6, 150.9, 150.0, 146.5, 134.63, 134.56, 132.7, 131.9, 131.8, 131.7, 129.9, 129.7, 129.5, 129.3, 127.5, 126.3, 124.01, 123.97, 123.94, 123.89, 123.76, 123.54, 122.34, 121.18, 119.7, 21.2, 21.0. The spectral data are in agreement with those reported in the literature.[6]

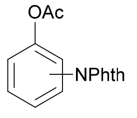


**3e**: Following the general procedure described above with **XI** (1.35 mg, 0.005 mmol, 0.05 equiv) and naphthalene (129.0 mg, 1.0 mmol, 10.0 equiv) as the arene source, **3e** was obtained as a white solid. The crude product was obtained as a mixture of two isomers (*a*/*b* = 7:1). The isomer ratios were determined through $^{1}$H-NMR. During purification by column chromatography, only major product was isolated (19.0 mg, 0.069 mmol, 69% yield). **$^{1}$H-NMR (600 MHz, $CDCl_3$)**: δ 8.01 (dd, $J$ = 5.4, 3.0 Hz, 2H), 7.99 (d, $J$ = 8.4 Hz, 1H), 7.94 (d, $J$ = 7.8 Hz, 1H), 7.84 (dd, $J$ = 5.4, 3.0 Hz, 2H), 7.64-7.58 (m, 2H), 7.55-7.44 (m, 3H). **$^{13}$C-NMR (150 MHz, $CDCl_3$)**: δ 167.9, 134.6, 132.1, 130.4, 130.0, 128.7, 128.3, 127.2, 127.1, 126.6, 125.5, 124.0, 122.5. The spectral data are in agreement with those reported in the literature.[3]

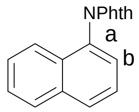


6. H. J. Kim, J. Kim, S. H. Cho and S. Chang, *J. Am. Chem. Soc.* 2011, **133**, 16382.

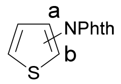


**3f**: Following the general procedure described above with **XI** (1.35 mg, 0.005 mmol, 0.05 equiv) and thiophene (0.08 mL, 1.0 mmol, 10.0 equiv) as the arene source, **3f** was obtained as a yellow solid (14.2 mg, 0.061 mmol, 61% yield). The crude product was obtained as a mixture of *a*/*b* = 4.2:1. The isolated product was an inseparable mixture of isomers; however, only the peaks for major isomer are reported. **$^1$H-NMR (600 MHz, $CDCl_3$)**: δ 7.95-7.93 (m, 2H), 7.80-7.77 (m, 2H), 7.53 (d, *J* = 3.6 Hz, 1H), 7.22 (dd, *J* = 5.4, 1.8 Hz, 1H), 7.06 (dd, *J* = 5.4, 3.6 Hz, 1H). **$^{13}$C-NMR (150 MHz, $CDCl_3$)**: δ 166.1, 134.8, 132.4, 131.6, 125.4, 124.1, 122.1, 120.6. The spectral data are consistent with those reported in the literature.[3]

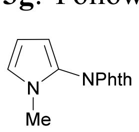


**3g**: Following the general procedure described above with **XI** (1.35 mg, 0.005 mmol, 0.05 equiv), $K_2CO_3$ (27.6 mg, 0.2 mmol, 2.0 equiv) and *N*-methylpyrrole (0.09 mL, 1.0 mmol, 10.0 equiv) as the arene source, **3g** was obtained as a white solid (15.2 mg, 0.069 mmol, 69% yield). **$^1$H-NMR (600 MHz, $CDCl_3$)**: δ 7.97 (dd, *J* = 5.4, 3.0 Hz, 2H), 7.81 (dd, *J* = 5.4, 3.0 Hz, 2H), 6.72 (m, 1H), 6.24 (t, *J* = 3.6 Hz, 1H), 6.19 (m, 1H), 3.48 (s, 3H). **$^{13}$C-NMR (150 MHz, $CDCl_3$)**: δ 167.8, 134.7, 131.9, 124.1, 122.3, 118.7, 107.7, 107.7, 33.3. The spectral data are consistent with those reported in the literature.[3]

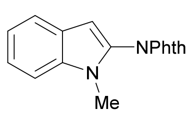


**3h**: Following the general procedure described above with **XI** (1.35 mg, 0.005 mmol, 0.05 equiv), $K_2CO_3$ (27.6 mg, 0.2 mmol, 2.0 equiv) and 1-methylindole (131 mg, 1.0 mmol, 10.0 equiv) as the arene source, **3h** was obtained as a yellow solid (19.6 mg, 0.072 mmol, 72% yield). **$^1$H-NMR (600 MHz, $CDCl_3$)**: δ 8.01 (dd, *J* = 5.4, 3.3 Hz, 2H), 7.85 (dd, *J* = 5.4, 3.3 Hz, 2H), 7.66 (d, *J* = 7.8 Hz, 1H), 7.36 (d, *J* = 8.4 Hz, 1H). 7.30 (t, *J* = 8.4 Hz, 1H), 7.16 (t, *J* = 7.8 Hz, 1H), 6.60 (s, 1H), 3.61 (s, 3H) **$^{13}$C-NMR (150 MHz, $CDCl_3$)**: δ 167.3, 136.2, 135.0, 132.0, 126.6, 124.3, 122.8, 121.5, 120.3, 109.8, 100.8, 29.7. The spectral data are consistent with those reported in the literature.[3]

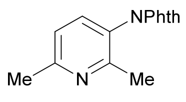


**3i**: Following the general procedure described above with **XI** (1.35 mg, 0.005 mmol, 0.05 equiv) and 2,6-lutidine (0.115 mL, 1.0 mmol, 10.0 equiv) as the arene source, **3i** was obtained as a white solid (17.1 mg, 0.068 mmol, 68% yield). **$^1$H-NMR (600 MHz, $CDCl_3$)**: δ 7.97 (dd, *J* = 5.4, 3.0 Hz, 2H), 7.82 (dd, *J* = 5.4, 3.0 Hz, 2H), 7.41 (d, *J* = 7.8 Hz, 1H), 7.15 (d, *J* = 7.8 Hz, 1H), 2.60 (s, 1H), 2.42 (s, 3H). **$^{13}$C-NMR (150 MHz, $CDCl_3$)**: δ 167.1, 159.2, 156.1, 136.8, 134.7, 132.1, 124.4, 124.1, 121.6, 24.5, 21.3. The spectral data are consistent with those reported in the literature.[3]

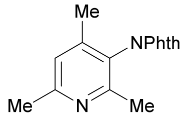


**3j**: Following the general procedure described above with **XI** (1.35 mg, 0.005 mmol, 0.05 equiv) and 2,4,6-colidine (0.133 mL, 1.0 mmol, 10.0 equiv) as the arene source, **3j** was obtained as a white solid (19.9 mg, 0.075 mmol, 75% yield). **$^1$H-NMR (600 MHz, $CDCl_3$)**: δ 7.97 (dd, *J* = 5.4, 3.0 Hz, 2H), 7.83 (dd, *J* = 5.4, 3.0 Hz, 2H), 7.01 (s, 1H), 2.55 (s,

3H), 2.36 (s, 3H), 2.14 (s, 3H). **$^{13}$C-NMR (150 MHz, $CDCl_3$)**: δ 167.1, 158.8, 156.4, 146.5, 134.7, 132.1, 124.1, 124.0, 123.4, 24.4, 21.1, 17.8. The spectral data are consistent with those reported in the literature. [3]

**3k**: Following the general procedure described above with **XI** (1.35 mg, 0.005 mmol, 0.05 equiv) and

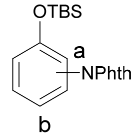


*tert*-butyldimethyl(phenoxy)silane (208 mg, 1.0 mmol, 10.0 equiv) as the arene source, **3k** was obtained as a white solid (25.1 mg, 0.071 mmol, 71% yield, *a*/*b* = 1:2). The isomer ratios were determined through $^1$H-NMR. The isolated product was an inseparable mixture of isomers. All peaks observed in the $^1$H-NMR and $^{13}$C-NMR were assigned. **FT-IR (thin film)**: 2929, 1735, 1714, 1558, 1508, 1381. **$^1$H-NMR (600 MHz, $CDCl_3$)**: δ 7.95-7.93 (m, 6.4H), 7.79-7.76 (m, 6.5H), 7.33 (m, 1H), 7.28 (d, *J* = 8.1 Hz, 4H), 7.23 (m, 1H), 7.08 (t, *J* = 6.9 Hz, 1H), 7.00 (d, *J* = 8.4 Hz, 1H), 6.94 (d, *J* = 7.8 Hz, 2H), 1.00 (s, 18H), 0.75 (s, 9H), 0.24 (s, 6H), 0.23 (s, 12H), 0.14 (s, 6H). **$^{13}$C-NMR (150 MHz, $CDCl_3$)**: δ 167.7, 167.5, 134.4, 134.3, 132.4, 132.0, 130.6, 130.3, 129.8, 127.9, 124.9, 123.8, 123.7, 121.7, 120.8, 120.6, 120.0, 115.4, 25.8, 25.5, 18.3, 17.9, –4.2, –4.3. **HRMS (ESI+)**: Calcd. for $C_{20}H_{23}O_3NNaSi$ ($[M + Na]^+$): 376.1339, Found: 376.1336.

**General procedure for the synthesis of authentic 3k:**

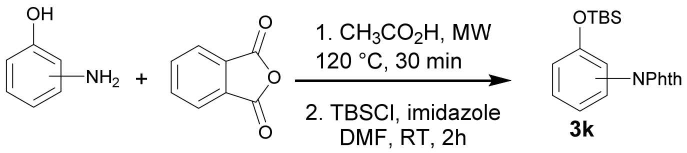


In a microwave reaction tube equipped with a magnetic stir bar, phthalic anhydride (740 mg, 5 mmol, 1.0 equiv), acetic acid (10.0 mL) and aniline derivative (5.0 mmol, 1.0 equiv) were taken. The mixture was heated at 120 °C for 30 min under microwave irradiation. The reaction mixture was cooled to rt and treated with water. A white precipitate was observed, which was filtered, washed with water and dried *in vacuo*. To the crude solid, TBSCl (900 mg, 6 mmol), imidazole (816 mg, 12 mmol) and DMF (10 mL) were added. The resulting mixture was stirred at rt for 2 h. The reaction mixture was cooled to 0 °C and a solution of 1N HCl (20 mL) was slowly added. The aqueous phase was extracted with diethyl ether (3 × 10 mL), washed with brine (1 × 30 mL), dried over $Na_2SO_4$ and concentrated under reduced pressure. Purification of the residue by flash column chromatography over silica gel afforded the desired product.

**3k (*ortho*)**: The general procedure described above was followed with 2-amino phenol (5.0 mmol, 1.29

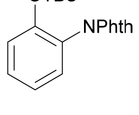


g) to obtain a white solid (882 mg, 51% yield). **FT-IR (thin film)**: 2927, 1735, 1716, 1504, 1382. **$^1$H-NMR (600 MHz, $CDCl_3$)**: δ 7.94 (dd, *J* = 5.4, 3.0 Hz, 2H), 7.77 (dd, *J* = 5.4, 3.0 Hz, 2H), 7.33 (dt, *J* = 7.8, 1.2 Hz, 1H), 7.26 (dd, *J* = 7.8, 1.2 Hz, 1H), 7.08 (dd, *J*

= 7.8, 1.2 Hz, 1H), 7.00 (dt, *J* = 7.8, 1.2 Hz, 1H), 0.75 (s, 9H), 0.14 (s, 6H). **$^{13}$C-NMR (150 MHz, CDCl$_3$)**: δ 167.5, 152.1, 134.3, 132.4, 130.6, 130.3, 123.7, 123.0, 121.7, 119.8, 25.5, 17.9, –4.3. **HRMS (ESI+)**: Calcd. for $C_{20}H_{23}O_3NNaSi$ ([M + Na]$^+$): 376.1339, Found: 376.1328.

**3k (*para*)**: The general procedure described above was followed with 4-amino phenol (5.0 mmol, 1.29 g) to obtain a white solid (1.412 g, 80% yield). **FT-IR (thin film)**: 2929, 1742, 1510, 1384. **$^1$H-NMR (600 MHz, CDCl$_3$)**: δ 7.93 (dd, *J* = 5.4, 3.0 Hz, 2H), 7.77 (dd, *J* = 5.4, 3.0 Hz, 2H), 7.27 (dd, *J* = 6.6, 2.1 Hz, 2H), 6.93 (dd, *J* = 6.6, 2.1 Hz, 2H), 0.99 (s, 9H), 0.23 (s, 6H). **$^{13}$C-NMR (150 MHz, CDCl$_3$)**: δ 167.7, 155.6, 134.4, 132.0, 127.9, 124.9, 123.8, 120.6, 25.8, 18.3, –4.2. **HRMS (ESI+)**: Calcd. for $C_{20}H_{23}O_3NNaSi$ ([M + Na]$^+$): 376.1339, Found: 376.1328.

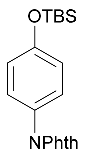


**3l**: Following the general procedure described above with **XI** (1.35 mg, 0.005 mmol, 0.05 equiv) and anisonitrile (0.133 mg, 1.0 mmol, 10.0 equiv) as the arene source, **3l** was obtained as a white solid (21.6 mg, 0.078 mmol, 78% yield). **FT-IR (thin film)**: 2846, 2225, 1778, 1608, 1506, 1373. **$^1$H-NMR (600 MHz, CDCl$_3$):** δ 7.96 (dd, *J* = 5.4, 3.0 Hz, 2H), 7.82 (dd, *J* = 5.4, 3.0 Hz, 2H), 7.76 (dd, *J* = 9.0, 2.4 Hz, 1H), 7.58 (d, *J* = 2.4 Hz, 1H), 7.11 (d, *J* = 9.0 Hz, 1H), 3.87 (s, 3H). **$^{13}$C-NMR (150 MHz, CDCl$_3$):** δ 166.8, 159.1, 135.2, 134.7, 134.2, 132.1, 124.1, 121.6, 118.2, 112.9, 104.7, 56.5. **HRMS (ESI+)**: Calcd. for $C_{16}H_{10}O_3N_2Na$ ([M + Na]$^+$): 301.0584, Found: 301.0579.

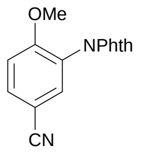


Recrystallization of **3l** from $CH_2Cl_2$/hexane (1:9) mixture afforded diffraction quality crystals. The thermal ellipsoids of non-hydrogen atoms are shown at the 50% probability level. Calculated hydrogen atoms are omitted for clarity. The atoms are shown through following colour sequence: blue = nitrogen, red = oxygen, grey = carbon.

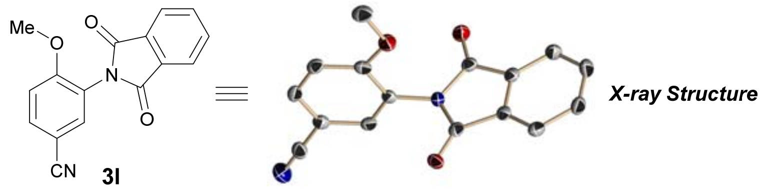


**3l**

**3m**: Following the general procedure described above with **XI** (1.35 mg, 0.005 mmol, 0.05 equiv) and anisole (0.10 mL, 1.0 mmol, 10.0 equiv) as the arene source, **3m** was obtained as a white solid (22.0 mg, 0.083 mmol, 83% yield, *o*/*m*/*p* = 1.0:0:1.2). The isomer ratios were determined through $^1$H-NMR. The isolated product was an inseparable mixture of isomers. All peaks observed in the $^1$H-NMR and $^{13}$C-NMR were assigned. **$^1$H-NMR (600 MHz, CDCl$_3$)**: δ 7.97-7.91 (m, 4H), 7.79-7.74 (m, 4H), 7.43 (t, *J* = 7.8 Hz, 1H), 7.34 (d, *J* = 9.0 Hz, 1H), 7.27-7.24 (m, 1H), 7.09-7.00 (m, 4H), 3.85 (s, 3H), 3.79 (s, 3.56H). **$^{13}$C-NMR (150 MHz, CDCl$_3$)**: δ 167.7, 167.5, 159.4, 155.5, 134.4, 134.2, 132.4, 131.9, 130.8, 130.1, 128.0, 124.4, 123.8, 120.9, 120.4, 114.6, 112.2, 55.9, 55.6. The spectral data are in agreement with those reported in the literature.[3]

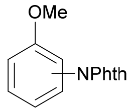

**3n**: Following the general procedure described above with **XI** (1.35 mg, 0.005 mmol, 0.05 equiv) and

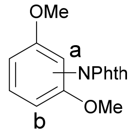

1,3-dimethoxybenzene (0.13 mL, 1.0 mmol, 10.0 equiv) as the arene source, **3n** was obtained as a white solid (24.0 mg, 0.085 mmol, 85% yield, *a*/*b* = 1:9). The isomer ratios were determined through $^{1}$H-NMR. The isolated product was an inseparable mixture of isomers, however, only the peaks for major isomer are reported. **$^{1}$H-NMR (400 MHz, DMSO-d$_6$)**: δ 7.91 (dd, *J* = 5.4, 2.4 Hz, 2H), 7.74 (dd, *J* = 5.4, 2.4 Hz, 2H), 7.26 (d, *J* = 8.8 Hz, 1H), 6.73 (d, *J* = 2.4 Hz, 1H), 6.64 (dd, *J* = 8.8, 2.4 Hz, 1H), 3.83 (s, 3H), 3.72 (s, 3H). **$^{13}$C-NMR (100 MHz, DMSO-d$_6$)**: δ 167.2, 161.2, 156.2, 134.8, 131.6, 130.9, 123.4, 112.8, 105.2, 99.2, 55.9, 55.6. The spectral data are in agreement with those reported in the literature.[7]

**3o**: Following the general procedure described above with **XI** (1.35 mg, 0.005 mmol, 0.05 equiv) and

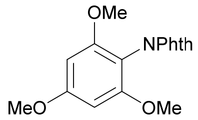

1,3,5-trimethoxybenzene (168.0 mg, 1.0 mmol, 10.0 equiv) as the arene source, **3o** was obtained as a white solid (25.0 mg, 0.080 mmol, 80% yield). **$^{1}$H-NMR (600 MHz, CDCl$_3$)**: δ 7.90 (dd, *J* = 5.4, 3.6 Hz, 2H), 7.73 (dd, *J* = 5.4, 3.6 Hz, 2H), 6.20 (s, 2H), 3.83 (s, 3H), 3.74 (s, 6H). **$^{13}$C-NMR (150 MHz, CDCl$_3$)**: δ 167.8, 162.2, 157.7, 133.9, 132.7, 123.6, 101.8, 91.2, 56.1, 55.6. The spectral data are in agreement with those reported in the literature.[3]

**3p**: Following the general procedure described above with **XI** (2.7 mg, 0.01 mmol, 0.1 equiv) and

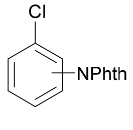

chlorobenzene (0.10 mL, 1.0 mmol, 10.0 equiv) as the arene source, **3p** was obtained as a white solid (20.9 mg, 0.081 mmol, 81% yield, *o*/*m*/*p* = 1.6:1:2). The isomer ratios were determined through $^{1}$H-NMR. The isolated product was an inseparable mixture of isomers. All peaks observed in the $^{1}$H-NMR and $^{13}$C-NMR were assigned. **$^{1}$H-NMR (600 MHz, CDCl$_3$): δ** 7.99-7.94 (m, 10H), 7.83-7.79 (m, 10H), 7.58 (m, 1.69H), 7.50 (m, 1H), 7.48 (m, 3.73H). 7.45-7.35 (m, 13H). **$^{13}$C-NMR (150 MHz, CDCl$_3$):** δ 167.1, 167.0, 166.8, 134.8, 134.7, 133.9, 133.4, 132.9, 132.1, 131.7, 130.8, 130.6, 130.3, 130.2, 129.8, 129.4, 139.3, 128.3, 127.9, 127.8, 127.6, 126.8, 124.7, 124.1, 124.0, 124.0. The spectral data are consistent with those reported in the literature. [8]

**3q**: Following the general procedure described above with **XI** (2.7 mg, 0.01 mmol, 0.1 equiv) and

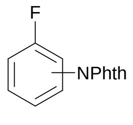

fluorobenzene (0.094 mL, 1.0 mmol, 10.0 equiv) as the arene source, **3q** was obtained as a white solid (13.3 mg, 0.058 mmol, 58% yield, *o*/*m*/*p* = 3:1:1). The isomer ratios were determined through $^{19}$F-NMR. The isolated product was an inseparable mixture of isomers. All peaks observed in the $^{1}$H-NMR, $^{13}$C-NMR and $^{19}$F-NMR were assigned. **$^{1}$H-NMR (500 MHz, CDCl$_3$)**: δ 7.99-7.94 (m, 9H), 7.83-7.79 (m, 9H), 7.50-7.40 (m, 6H), 7.37 (dt, *J* = 7.5, 1.5 Hz, 0.88H), 7.31-7.27 (m, 3H), 7.24-7.18 (m, 4H), 7.12 (dt, *J* = 8.5, 1.5 Hz, 1H). **$^{13}$C-NMR (125 MHz, CDCl$_3$)**: δ 167.4, 167.0, 166.7, 163.9, 163.8, 163.1, 161.1, 134.8, 134.7, 134.6, 132.1, 131.8, 131.7, 131.08, 131.0, 130.4, 130.3, 130.0, 128.6, 128.5, 127.7, 124.8, 124.13, 124.06, 124.02, 122.2, 122.1, 117.0,

---

7. J. Vamecq, P. Bac, C. Herrenknecht, P. Maurois, P. Delcourt and J. P. Stables, *J. Med. Chem.* 2000, **43**, 1311.
8. H. Kim, T. Kim, D. G. Lee, A. W. Roh and C. Lee, *Chem. Commun.* 2014, **50**, 9273.

116.9, 116.4, 116.2, 115.2, 115.1, 114.2, 114.0. **$^{19}$F-NMR (471 MHz, $CDCl_3$):** δ –111.1, –112.9, –118.6. The spectral data are consistent with those reported in the literature.[8]

**3r**: Following the general procedure described above with **XI** (2.7 mg, 0.01 mmol, 0.1 equiv) and

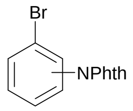


bromobenzene (0.104 mL, 1.0 mmol, 10.0 equiv) as the arene source, **3r** was obtained as a white solid (21.4 mg, 0.071 mmol, 71% yield, *o*/*m*/*p* = 1.4:1.0:1.7). The isomer ratios were determined through $^1$H-NMR. The isolated product was an inseparable mixture of isomers. All peaks observed in the $^1$H-NMR and $^{13}$C-NMR were assigned. **$^1$H-NMR (600 MHz, $CDCl_3$)**: δ 7.99-7.95 (m, 9H), 7.82-7.80 (m, 9H), 7.75 (dd, *J* = 7.8, 1.2 Hz, 1.38H), 7.66 (m, 1.01H), 7.64 (dd, *J* = 6.5, 1.8 Hz, 3.36H), 7.54 (dd, *J* = 6.3, 1.5 Hz, 1.00H), 7.47 (dt, *J* = 7.8, 1.2 Hz, 1.40H), 7.43 (dd, *J* = 8.4, 1.2 Hz, 0.91H), 7.39-7.34 (m, 8H). **$^{13}$C-NMR (150 MHz, $CDCl_3$)**: δ 167.1, 167.0, 166.8, 134.8, 134.7, 134.6, 133.7, 133.1, 132.4, 132.1, 131.8, 131.7, 131.6, 131.3, 131.1, 130.9, 130.4, 129.7, 128.6, 128.1, 125.2, 124.1, 124.1, 124.0, 123.5, 122.6, 122.0. The spectral data are consistent with those reported in the literature.[8]

**3s**: Following the general procedure described above with **XI** (2.7 mg, 0.01 mmol, 0.1 equiv) and

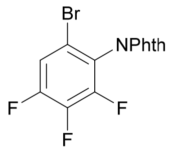


1-bromo-3,4,5-trifluorobenzene (0.12 mL, 1.0 mmol, 10.0 equiv) as the arene source, **3s** was obtained as a white solid (18.0 mg, 0.05 mmol, 50% yield). **FT-IR (thin film):** 1753, 1508, 1481, 1379, 1103, 1076. **$^1$H-NMR (600 MHz, $CDCl_3$)**: δ 7.99 (dd, *J* = 5.4, 3.0 Hz, 2H), 7.84 (dd, *J* = 5.4, 3.0 Hz, 2H), 7.45-7.39 (m, 1H). **$^{13}$C-NMR (150 MHz, $CDCl_3$)**: δ 165.4, 152.5 (dd, *J* = 10.5, 4.2 Hz), 150.8 (dd, *J* = 11.2, 3.5 Hz), 150.1 (dd, *J* = 10.5, 4.2 Hz), 148.4 (dd, *J* = 11.2, 4.4 Hz), 139.0 (m), 134.9, 131.8, 124.4, 118.2-117.9 (m), 116.6 (dd, *J* = 21.6, 4.2 Hz). **HRMS (APCI-MS):** Calcd for $C_{14}H_5NO_2BrF_3$ ($[M]^+$): 354.9450, Found: 354.9452.

**3t**: Following the general procedure described above with caffeine (19.4 mg, 0.1 mmol, 1.0 equiv) as

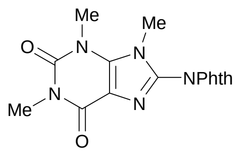


the arene source, **2** (52.4 mg, 0.13 mmol, 1.3 equiv) and **XI** (2.7 mg, 0.01 mmol, 0.1 equiv), **3t** was obtained as a white solid (20.0 mg, 0.059 mmol, 59% yield). **$^1$H-NMR (600 MHz, $CDCl_3$)**: δ 8.00 (dd, *J* = 5.4, 3.0 Hz, 2H), 7.88 (dd, *J* = 5.4, 3.0 Hz, 2H), 3.88 (s, 3H), 3.57 (s, 3H), 3.42 (s, 3H). **$^{13}$C-NMR (150 MHz, $CDCl_3$)**: δ 166.5, 155.3, 151.5, 146.9, 137.0, 135.5, 131.5, 124.8, 108.3, 32.4, 30.0, 28.1. The spectral data are in agreement with those reported in the literature.[3]

## F. Calculation of $\Delta G_{et}$ by Rehm-Weller Equation:

The $\Delta G_{et}$ (et: electron transfer) was calculated by the following equation:

$$\Delta G_{et} = E_{ox}\,(D) - E_{red}\,(A) - \Delta E_{triplet} + \Delta E_{coloumbic}$$

$E_{ox}$ (D) is the oxidation potential of the donor, $E_{red}$ (A) is the reduction potential of the acceptor, $\Delta E_{triplet}$ is the triplet energy of the ketone and $\Delta E_{columbic}$ is the term for columbic interaction in a particular solvent. The value of $\Delta E_{coloumbic}$ is usually very small in polar solvents (~ 0.03 eV for $CH_3CN$) and thus this parameter is not incorporated in the calculations. The calculated values are listed below:

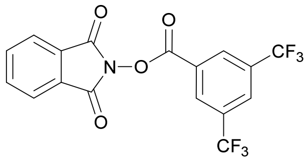


$E_{red}$ = – 1.1 V vs. SCE

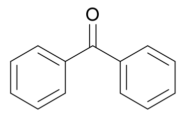


$E_{ox}$ = + 2.4 V vs. SCE
$E_{triplet}$ = + 3.0 eV
$\Delta G_{et}$ = + 11.5 kcal/mol

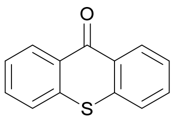


$E_{ox}$ = + 1.7 V vs. SCE
$E_{triplet}$ = + 2.8 eV
$\Delta G_{et}$ = + 0 kcal/mol

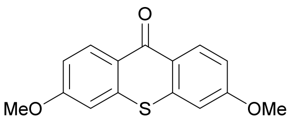


$E_{ox}$ = + 1.7 V vs. SCE
$E_{triplet}$ = + 3.0 eV
$\Delta G_{et}$ = – 4.61 kcal/mol

## G. UV-Visible Absorption Spectroscopy:

UV-Visible absorption spectra of ketone catalysts were measured in $CH_3CN$ (for Spectrochemical Analysis) on a Shimadzu UV-3510 spectrometer.

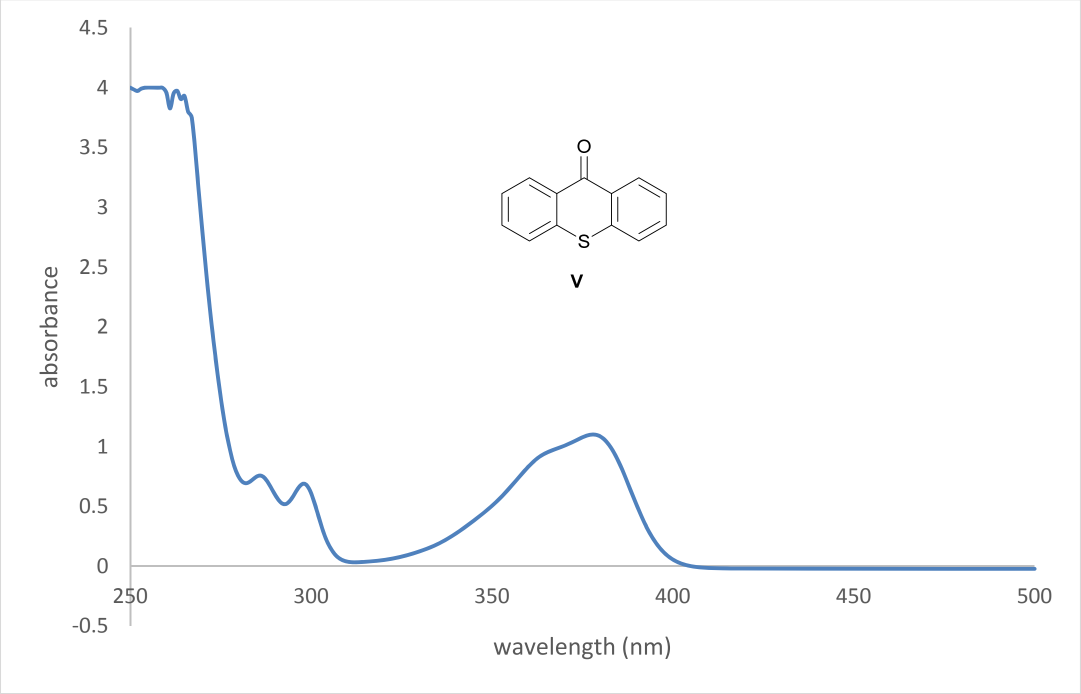


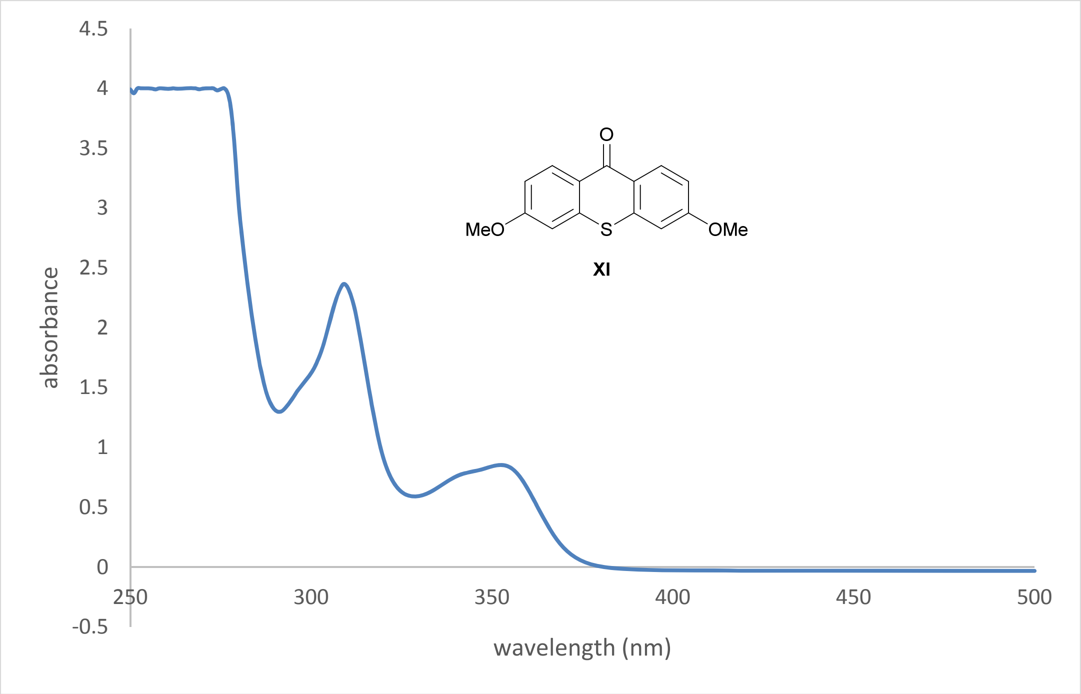

## H. Phosphorescence Spectroscopy:

Phosphorescence spectra of ketone catalysts were measured at 77 K in MeOH (for Spectrochemical Analysis) with an appropriate wave length, delay time, and gate time. Experiments were performed on a LS-55 luminescence spectrometer (Perkin-Elmer Ltd, Beaconsfield, UK) equipped with low temperature phosphorescence measurement unit using a quartz tube (5 mm OD) as a sample cell.

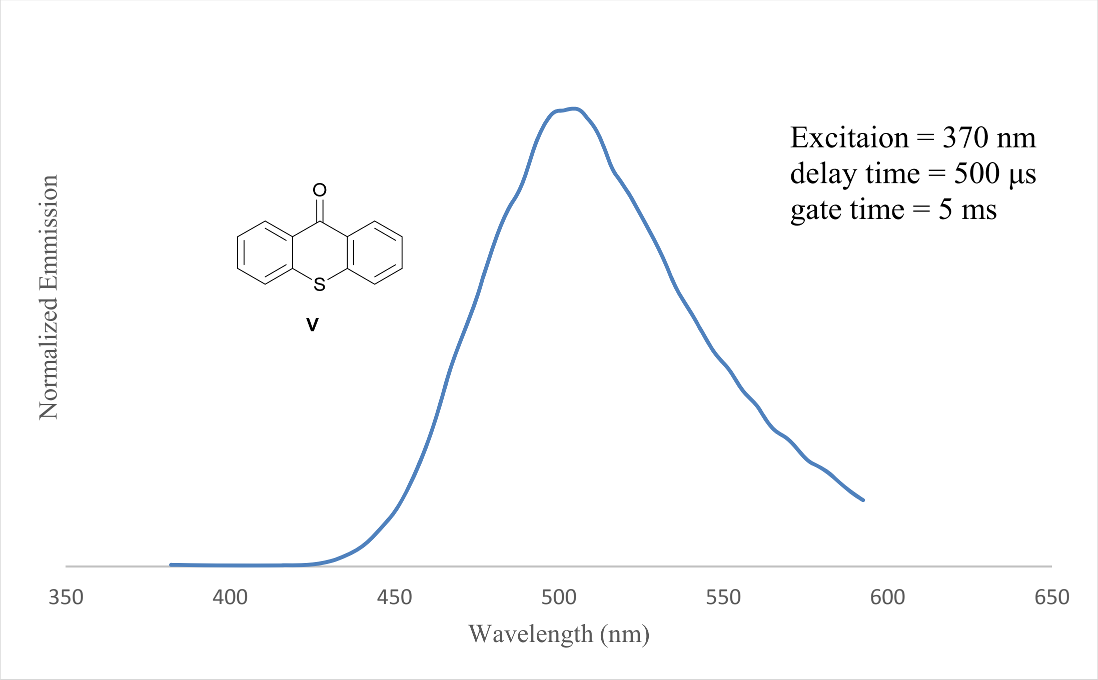


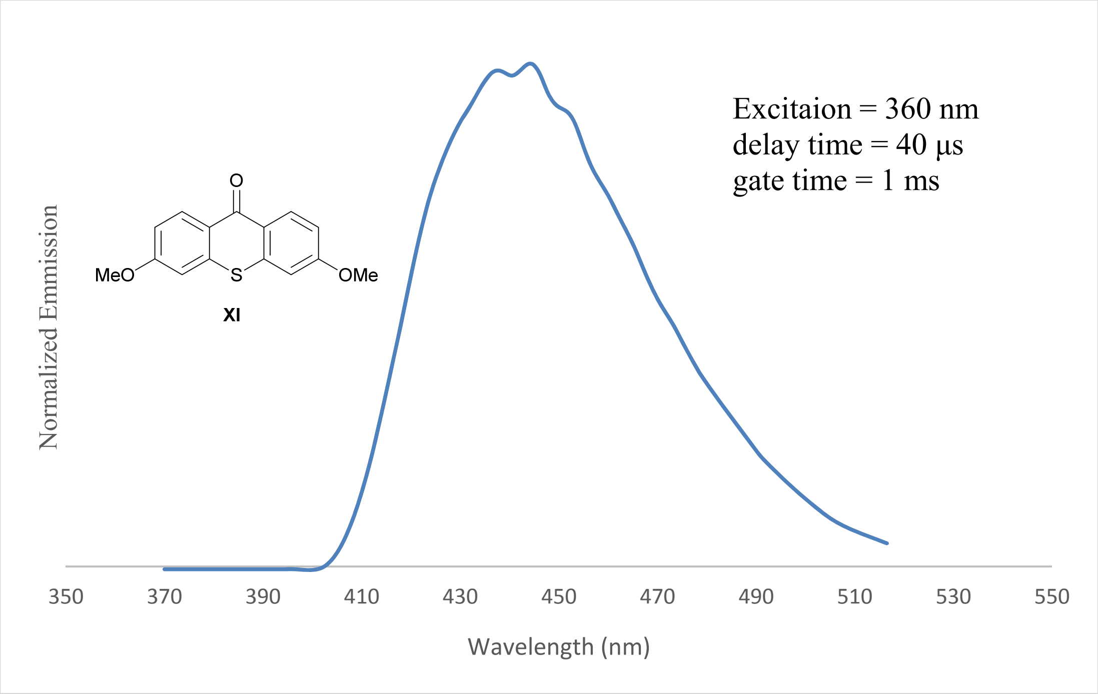

**I. Electrochemical Studies:** Cyclic voltammetry was performed on an ALS/chi-617A electrochemical analyser. The voltammetric cell consisted of a glassy carbon electrode, a Pt wire counter electrode and an Ag/$AgNO_3$ reference electrode. The measurements were carried out under $N_2$ using a solution of sample with a concentration of 1.0 mM in $CH_3CN$ containing $Bu_4NClO_4$ as a supporting electrolyte (0.10 M). The scan rate was 100 mV/s. Nitrogen was passed through the sample between measurements to avoid the deleterious influence of oxygen. The redox potentials were calibrated to the SCE scale with a ferrocene/ferrocenium ion couple.

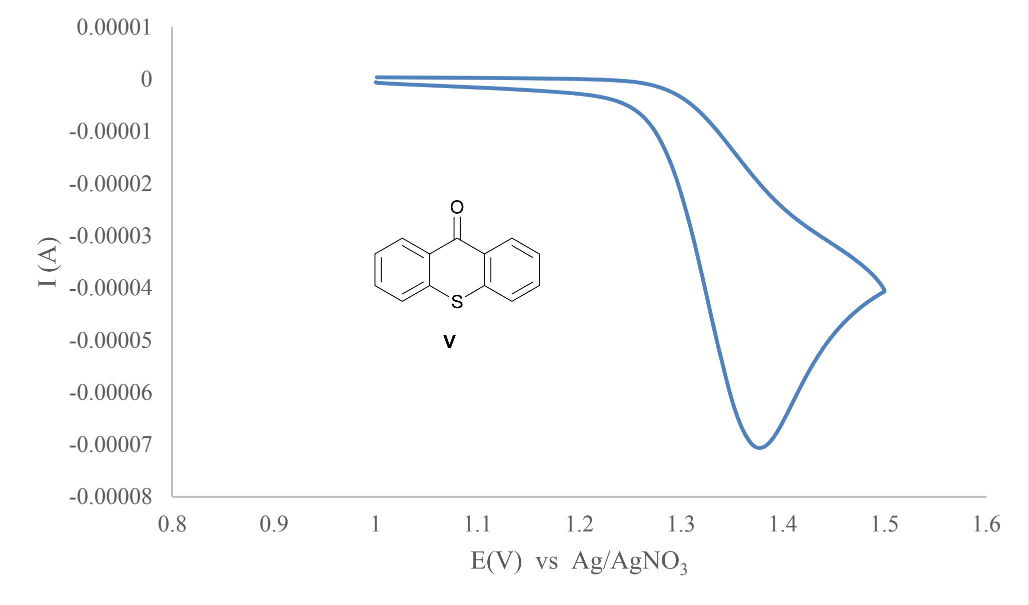


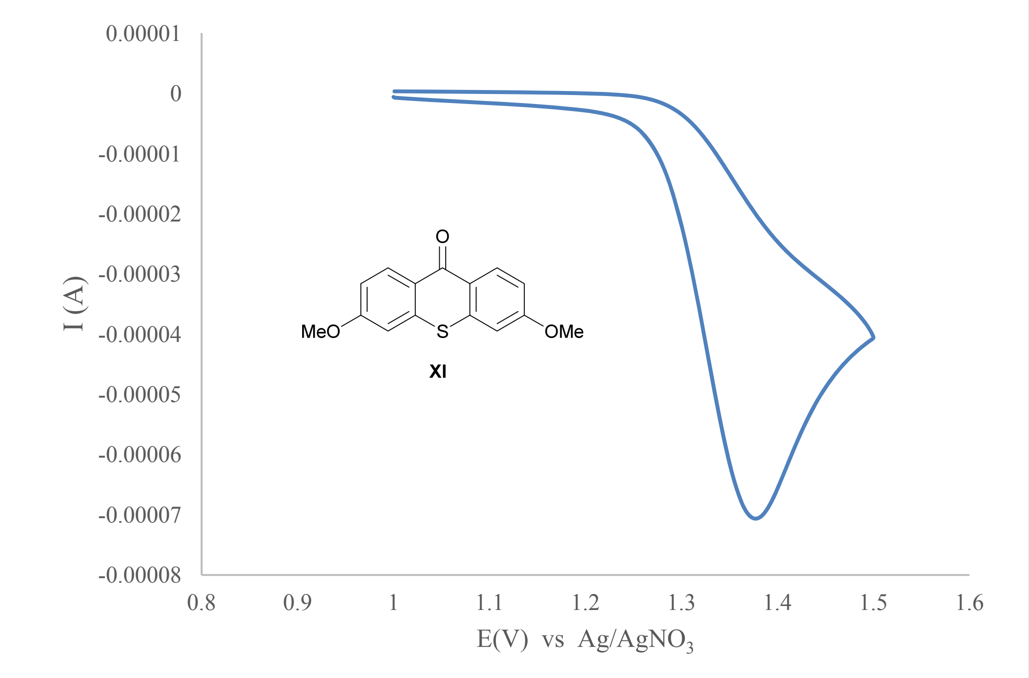

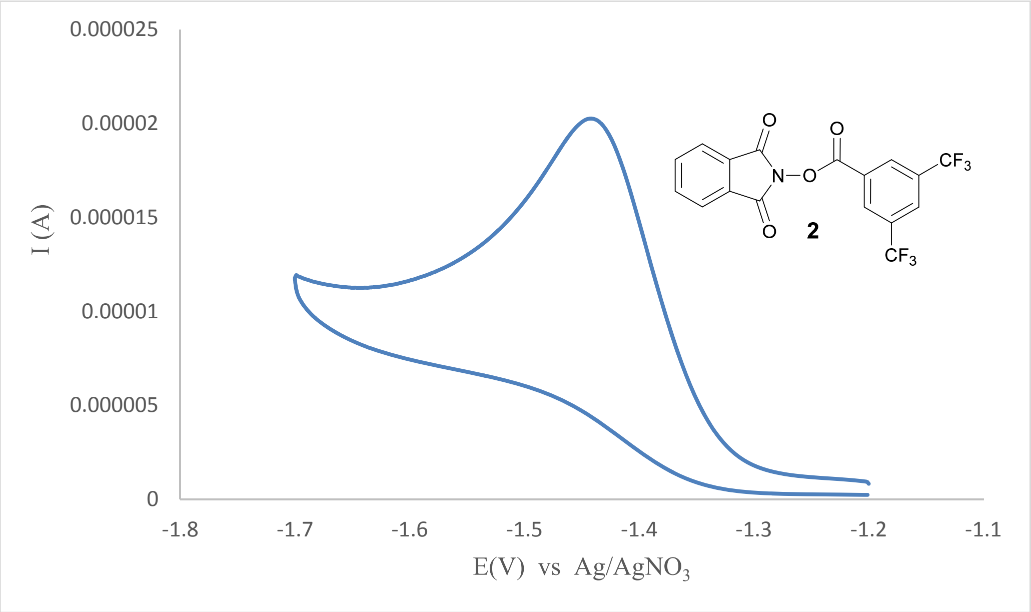
0.000025
0.00002
0.000015
0.00001
0.000005
0
I (A)
-1.8
-1.7
-1.6
-1.5
-1.4
-1.3
-1.2
-1.1
E(V) vs Ag/AgNO3
CF3
CF3
N
O
O
O
O
2

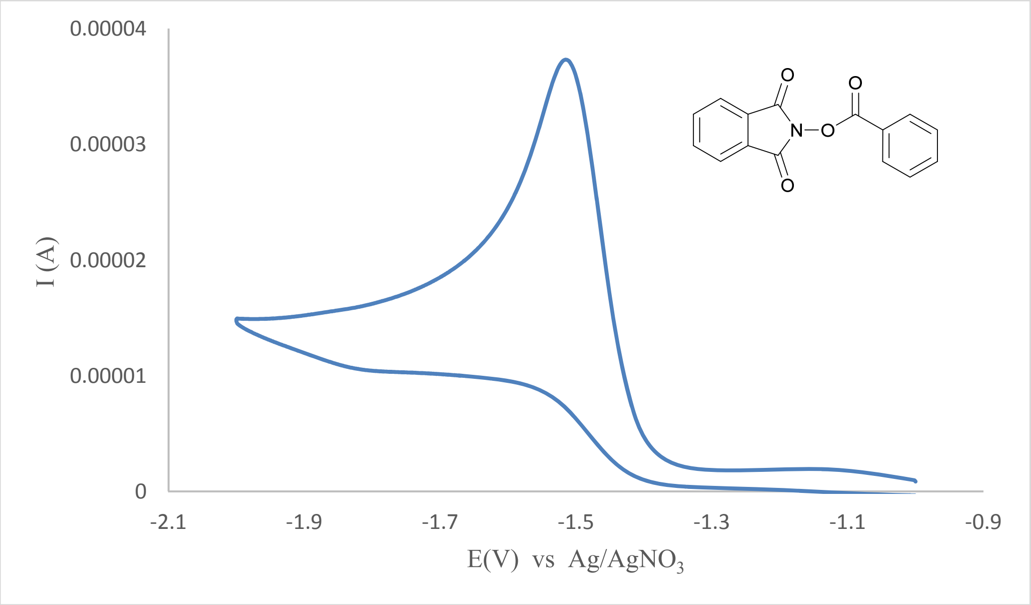
0.00004
0.00003
0.00002
0.00001
0
I (A)
-2.1
-1.9
-1.7
-1.5
-1.3
-1.1
-0.9
E(V) vs Ag/AgNO3
N
O
O
O
O

**J. Determination of Excited State Potentials:**

Excited state potentials are estimated by using the Rehm-Weller equations as shown below.[9]

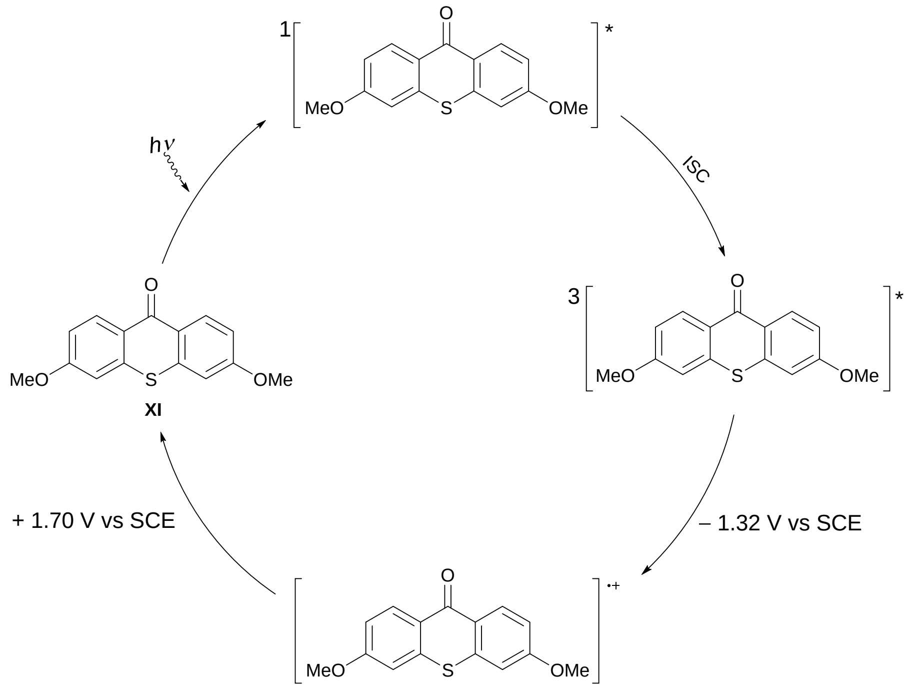


$$E^{O^*}{}_{Red} = E^{O'}{}_{Red} - E^{0-0}$$

Where $E^{O^*}$ represents the excited state potential, $E^{O'}$ stands for the ground state potential and $E^{0\text{-}0}$ refers to the energy gap between the zeroeth vibrational levels of the ground and excited state. $E_{Red}$ refers to the ketone/ketone(radical cation) couples. $E^{0\text{-}0}$ is approximated as the high energy onset of phosphorescence, where the emission intensity is 10% of that obtained at the maximum emission wavelength, using the "10% rule".[10]

$$E^{O^*}{}_{Red} = E^{O'}{}_{Red} - E^{0-0} = 1.70 - 3.02 = -1.32\ V\ vs\ SCE$$

9. J. L. Brennan, T. E. Keyes and R. J. Forster, *Langmuir* 2006, **22**, 10754.
10. A. Dossing, C. K. Ryu, S. Kudo and P. C. Ford, *J. Am. Chem. Soc.* 1993, **115**, 5132.

## K. Quantum Yield Measurement:

The quantum yield was measured by standard ferrioxalate actinometry.[11] A 300W Xenon lamp (5% of light intensity, 365 ± 5 nm band pass filter high transmittance) was used as the light source. The measurements were carried out by following the modified literature procedure.[12] The solutions were prepared and stored in dark room. Potassium ferrioxalate solution (0.15 M): 2.21 g of potassium ferrioxalate hydrate was dissolved in 30 mL of 0.05 M $H_2SO_4$. Buffered solution of phenanthroline: 50 mg of 1,10-phenanthroline and 11.25 g of sodium acetate were dissolved in 50 mL of 0.5 M $H_2SO_4$.

### a) Measurement of light intensity at 365 nm

To determine the photon flux of the spectrophotometer, 2.0 mL of ferrioxalate solution was placed in a cuvette and irradiated for 90.0 seconds at 365 nm with an emission slit width at 10.0 nm. After irradiation, 0.35 mL of phenanthroline solution was added to the cuvette. To ensure complete coordination of ferrous ions with phenanthroline, the solution was kept for 1 h. The absorbance of the solution was measured at 510 nm. A non-irradiated (in dark) sample was also prepared and the absorbance at 510 nm was measured. Conversion was calculated using equation 1.

$$mol\ Fe^{2+} = \frac{V \times \Delta A\ (510nm)}{L \times \varepsilon\ (510nm)} \qquad (1)$$

Where V is the total volume (0.00235 L) of the solution after addition of phenanthroline, ΔA is the difference in absorbance at 510 nm between irradiated and non-irradiated solutions, l is the path length (1.000 cm), and ε is the molar absorptivity at 510 nm (11,100 L $mol^{-1}$ $cm^{-1}$). The photon flux can be calculated using equation 2.

$$photon\ flux = \frac{mol\ Fe^{2+}}{\Phi \times t \times f} \qquad (2)$$

$$f = 1 - 10^{-A} \qquad (3)$$

Where *Φ* is the quantum yield for ferrioxalate actinometer (1.21 for a 0.15 M solution at λ = 366 nm),[10] t is the time (10 s, 20 s, 30 s, 40 s), and f is the fraction of light absorbed at 365 nm. The photon flux was calculated to be $5.00 \times 10^{-9}$ einstein/sec.

---

11. M. Montalti, A. Credi, L. Prodi and M. T. Gandolf, Handbook of photochemistry (3rd Ed.), New York, 2006.
12. M. A. Cismesia and T. P. Yoon, *Chem. Sci.*, 2015, **6**, 5426.

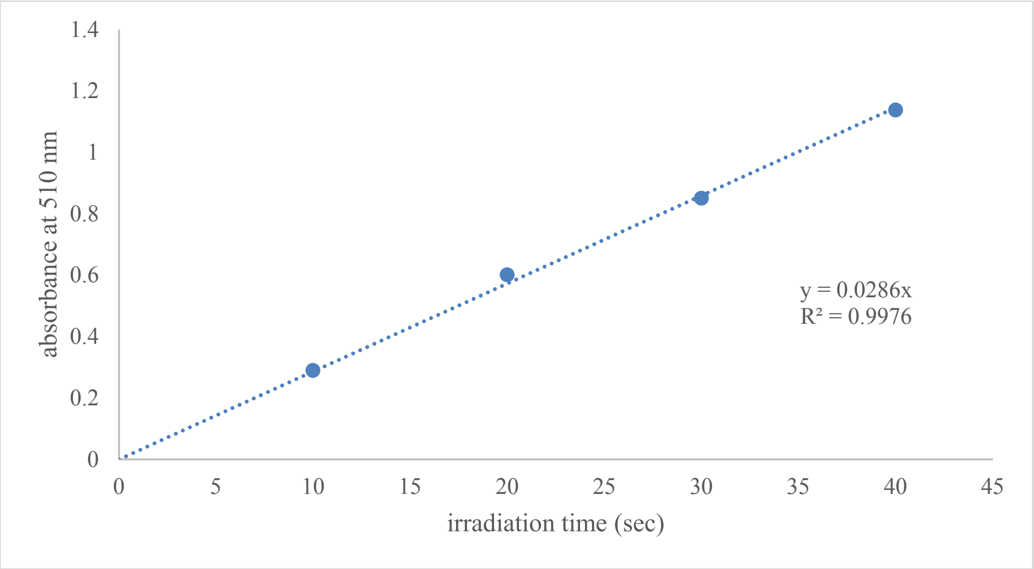


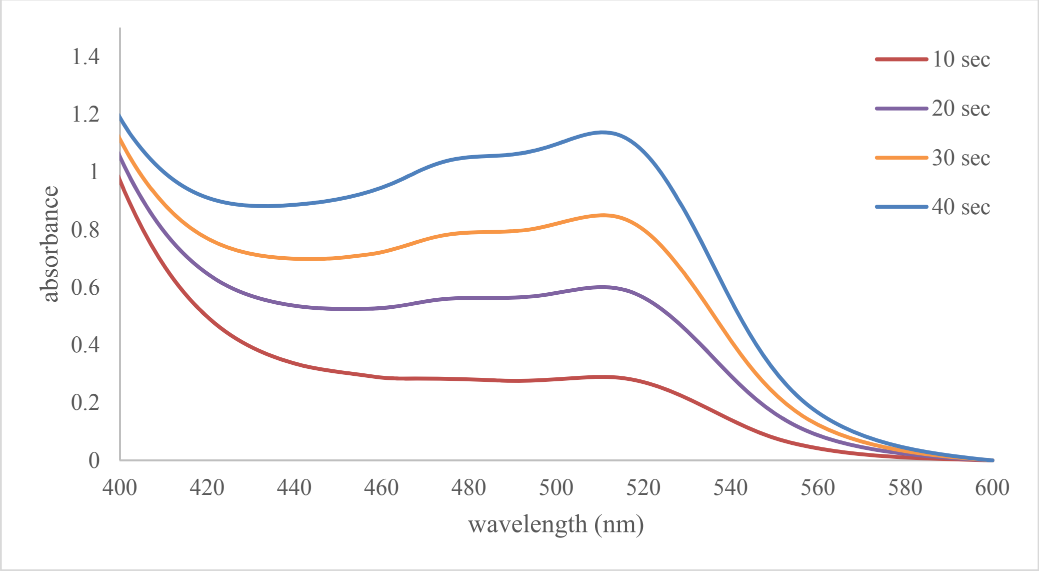


**Determination of fraction of light absorbed at 365 nm for the ferrioxalate solution:**

The absorbance of above ferrioxalate solution at 365 nm was measured to be > 3. The fraction of light absorbed (f) by this solution was calculated using equation 3.

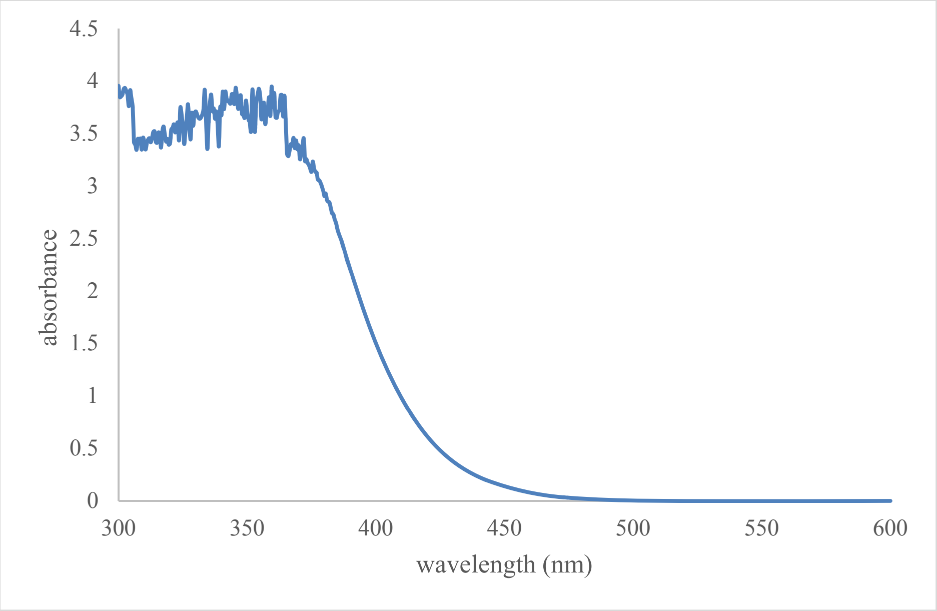


**b) Measurement of quantum yield**

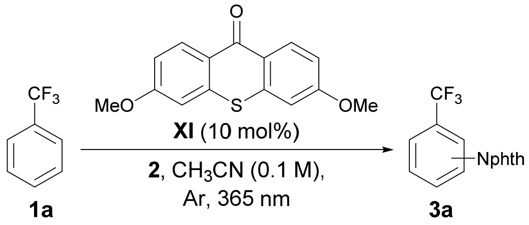


A screw-top cuvette was charged with the catalyst **XI** (10 mol%), **1a** (0.24 mL, 2.0 mmol, 10.0 equiv), **2** (0.2 mmol, 80 mg, 1.0 equiv), $CH_3CN$ (2 mL, 0.1 M), and a small magnetic stir bar was also introduced. The cuvette was degassed with a nitrogen stream for 10 min. After degassing, the reaction mixture was stirred and irradiated with 150 W Xenon lamp (5% of light intensity, 365 nm ± 5 nm band pass filter) for 86400 s (24 h). After irradiation, the reaction mixture was evaporated. The reaction yield was determined through $^1$H-NMR by using 1,3,5-trimethoxybenzene as internal standard. Essentially all the incident light ($f > 0.999$, vide infra) is absorbed by the catalyst under the conditions described above. The quantum yield is calculated as shown below:

$$\Phi = \frac{mol\ product}{flux \times t \times f}$$

$$\Phi = \frac{1.54 \times 10^{-5}\ mol}{5.004 \times 10^{-9} \times 86400 \times 1} = 0.036$$

**L. Crystallographic Structure Determination of 3l:** The single crystal obtained by recrystallisation in $CH_2Cl_2$/hexane (1:9) at room temperature was mounted on MicroMesh. X-ray diffraction data was collected at 123 K on a Rigaku VariMax with a Pilatus diffractometer and a fine-focus sealed tube Mo/Kα radiation (λ = 0.71075 Å). An absorption correction was made using Crystal Clear. The structure was solved by direct methods and Fourier syntheses. The data was refined by full-matrix least squares on $F^2$ by using SHELXL-2014.[13] All non-hydrogen atoms were refined with anisotropic displacement parameters. The other hydrogen atoms were placed in a calculated positions and isotropic thermal parameters were refined. The crystallographic data are summarized in the table below and the ORTEP diagram is shown on page S-10.

Crystal data and structure refinement for **3l**.

| | | |
|---|---|---|
| Identification code | **3l** | |
| Empirical formula | $C_{16} H_{10} N_2 O_3$ | |
| Formula weight | 278.26 | |
| Temperature | 123 (2) K | |
| Wavelength | 71.073 pm | |
| Crystal system | Orthorhombic | |
| Space group | F d d 2 | |
| Unit cell dimensions | a = 23.536(12) Å | α= 90°. |
| | b = 42.75(2) Å | β= 90°. |
| | c = 5.126(3) Å | γ = 90°. |
| Volume | 5158(5) Å | |
| Z | 16 | |
| Density (calculated) | 1.433 mg/m3 | |
| Absorption coefficient | 0.101 $mm^{-1}$ | |
| F(000) | 2304 | |
| Crystal size | 0.350 × 0.050 × 0.030 $mm^3$ | |
| Theta range for data collection | 3.343 to 25.493°. | |
| Index ranges | $-28 \leq h \leq 28$, $-51 \leq k \leq 51$, $-6 \leq l \leq 6$ | |
| Reflections collected | 11161 | |
| Independent reflections | 2364 [$R_{(int)}$ = 0.0934] | |
| Completeness to θ = 25.242° | 99.2 % | |
| Absorption correction | Semi-empirical from equivalents | |
| Max. and min. transmission | 1.000 and 0.799 | |
| Refinement method | Full-matrix least-squares on $F^2$ | |
| Data / restraints / parameters | 2364 / 1 / 191 | |

13. G. Sheldrick, *Acta Cryst. C* 2015, **71**, 3.

| | |
|---|---|
| Goodness-of-fit on $F^2$ | 0.889 |
| Final R indices [I > σ (I)] | R1 = 0.0372, ω R2 = 0.0805 |
| R indices (all data) | R1 = 0.0438, ω R2 = 0.0816 |
| Absolute structure parameter | –1.0(10) |
| Extinction coefficient | 0 |
| Largest diff. peak and hole | 0.157 and -0.188 e.$Å^{-3}$ |

**M. Computational Studies**

**M.1 Computational Methods:** Density functional theory (DFT)[14] calculations was performed using the Gaussian 09 program (Revision D.01)[15] and the results are produced with Gauss View 5.0.8. All calculations were performed at the B3LYP/6-311+G (d,p) level of theory, followed by frequency calculations at the same level. Homolytic bond dissociation enthalpies (BDE) were calculated using B3LYP/6-311+G (d,p). The results are summarized below.

**M.2 N–O Bond BDEs–DFT Studies**: The DFT studies were performed for determining the N–O bond dissociation energies of imidating agent (**2**) in the ground state, triplet excited state and the corresponding radical anion.

| N–O bond cleavage | N–O bond dissociation energy (kcal/mol) |
|---|---|
| 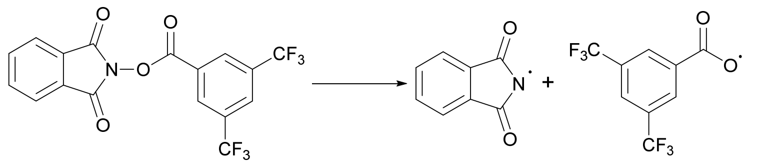 | 55.50 |
| 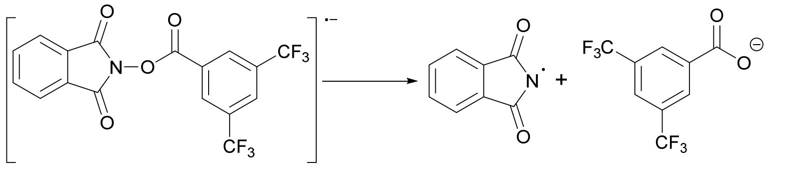 | 0.83 |

14. R. G. Parr, *Density-Functional Theory of Atoms and Molecules* 1989, Oxford University Press, Oxford, UK.
15. Gaussian 09, Revision B.01: M. J. Frisch, G. W. Trucks, H. B. Schlegel, G. E. Scuseria, M. A. Robb, J. R. Cheeseman, G. Scalmani, V. Barone, B. Mennucci, G. A. Petersson, H. Nakatsuji, M. Caricato, X. Li, H. P. Hratchian, A. F. Izmaylov, J. Bloino, G. Zheng, J. L. Sonnenberg, M. Hada, M. Ehara, K. Toyota, R. Fukuda, J. Hasegawa, M. Ishida, T. Nakajima, Y. Honda, O. Kitao, H. Nakai, T. Vreven, J. A. Montgomery, Jr., J. E. Peralta, F. Ogliaro, M. Bearpark, J. J. Heyd, E. Brothers, K. N. Kudin, V. N. Staroverov, T. Keith, R. Kobayashi, J. Normand, K. Raghavachari, A. Rendell, J. C. Burant, S. S. Iyengar, J. Tomasi, M. Cossi, N. Rega, J. M. Millam, M. Klene, J. E. Knox, J. B. Cross, V. Bakken, C. Adamo, J. Jaramillo, R. Gomperts, R. E. Stratmann, O. Yazyev, A. J. Austin, R. Cammi, C. Pomelli, J. W. Ochterski, R. L. Martin, K. Morokuma, V. G. Zakrzewski, G. A. Voth, P. Salvador, J. J. Dannenberg, S. Dapprich, A. D. Daniels, O. Farkas, J. B. Foresman, J. V. Ortiz, J. Cioslowski, and D. J. Fox, Gaussian, Inc., Wallingford CT, 2010.

| | |
|---|---|
| 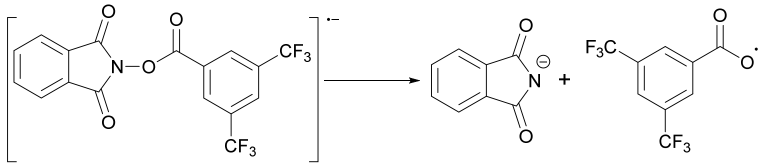 | 5.96 |
| 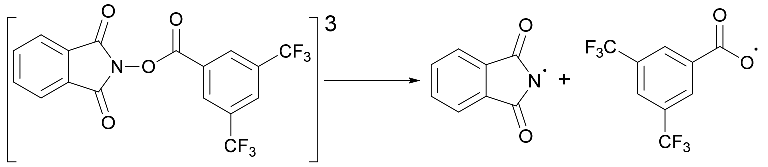 | –11.61 |
| 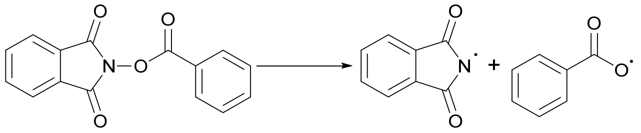 | 55.36 |
| 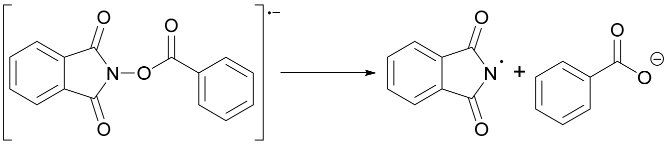 | 7.24 |
| 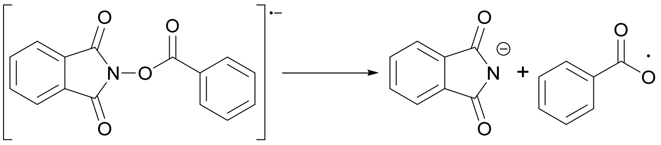 | –4.80 |
| 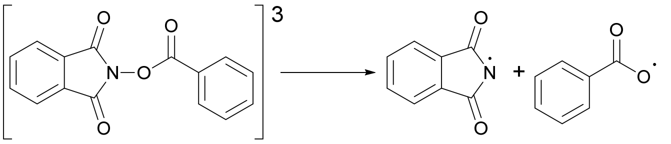 | –11.74 |

**M.3 Triplet Excited Energy of 2:** Energy of the imidating agents (2) in triplet excited state is determined through DFT calculations.

| Imidating agent (**2**) | Triplet excited energy (kcal/mol) |
|---|---|
| 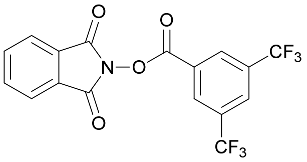 | 74.26 |

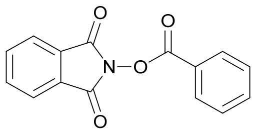

74.45

## M.4 Optimized Structures and Cartesian Coordinates

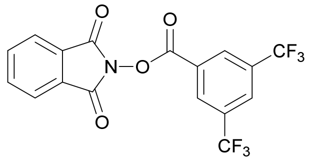

SCF Done: E(RB3LYP) = –1607.19140108 A.U.

Total Energy: E(TD-HF/TD-KS) = –1607.07298428 A.U.

Excited State 1: Triplet-A 3.2223 eV, 384.77 nm, f=0.0000 <S**2> = 2.000

| Center number | Atomic number | Atomic type | Coordinates (Angstroms) | | |
|---|---|---|---|---|---|
| | | | X | Y | Z |
| 1 | 6 | 0 | –2.896743 | 1.235918 | –0.039927 |
| 2 | 6 | 0 | –1.521133 | 1.030606 | –0.029458 |
| 3 | 6 | 0 | –1.021660 | –0.275420 | –0.016929 |
| 4 | 6 | 0 | –1.896742 | –1.362566 | –0.012922 |
| 5 | 6 | 0 | –3.270925 | –1.141901 | –0.021299 |
| 6 | 6 | 0 | –3.777575 | 0.154749 | –0.031947 |
| 7 | 1 | 0 | –0.842779 | 1.872022 | –0.040205 |
| 8 | 1 | 0 | –1.491434 | –2.366691 | –0.010356 |
| 9 | 1 | 0 | –4.846185 | 0.321404 | –0.047475 |
| 10 | 6 | 0 | 0.434271 | –0.575749 | –0.010840 |
| 11 | 8 | 0 | 0.937180 | –1.658620 | 0.004280 |
| 12 | 8 | 0 | 1.174495 | 0.608129 | –0.024197 |
| 13 | 7 | 0 | 2.529140 | 0.402703 | –0.014268 |
| 14 | 6 | 0 | 3.263475 | 0.252218 | 1.182257 |
| 15 | 8 | 0 | 2.812188 | 0.310297 | 2.294646 |
| 16 | 6 | 0 | 3.276418 | 0.216935 | –1.197731 |
| 17 | 8 | 0 | 2.837481 | 0.242023 | –2.316264 |
| 18 | 6 | 0 | 5.834389 | –0.202728 | –1.408400 |
| 19 | 6 | 0 | 4.664818 | 0.001084 | –0.695664 |
| 20 | 6 | 0 | 4.657171 | 0.021872 | 0.702092 |

| | | | | | |
|---|---|---|---|---|---|
| 21 | 6 | 0 | 5.818754 | –0.160353 | 1.433484 |
| 22 | 6 | 0 | 7.004865 | –0.367232 | 0.720653 |
| 23 | 6 | 0 | 7.012536 | –0.388102 | –0.676654 |
| 24 | 1 | 0 | 5.830206 | –0.219194 | –2.491436 |
| 25 | 1 | 0 | 5.802545 | –0.144629 | 2.516418 |
| 26 | 1 | 0 | 7.933212 | –0.515531 | 1.259825 |
| 27 | 1 | 0 | 7.946745 | –0.552283 | –1.200894 |
| 28 | 6 | 0 | –4.203281 | –2.328451 | 0.011783 |
| 29 | 6 | 0 | –3.446773 | 2.640819 | –0.008234 |
| 30 | 9 | 0 | –4.204325 | –2.921178 | 1.228620 |
| 31 | 9 | 0 | –5.477126 | –1.982506 | –0.262623 |
| 32 | 9 | 0 | –3.838346 | –3.273176 | –0.881572 |
| 33 | 9 | 0 | –3.641478 | 3.066068 | 1.263048 |
| 34 | 9 | 0 | –4.638300 | 2.727358 | –0.636644 |
| 35 | 9 | 0 | –2.613561 | –3.523564 | –0.594752 |

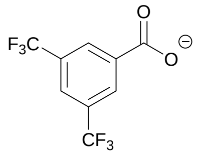


SCF Done: E(RB3LYP) = 1094.71336365 A.U.
Sum of electronic and thermal Enthalpies = –1094.590115 A.U

| Center number | Atomic number | Atomic type | Coordinates (Angstroms) | | |
|---|---|---|---|---|---|
| | | | X | Y | Z |
| 1 | 6 | 0 | 1.199210 | –0.431810 | 0.000000 |
| 2 | 6 | 0 | 1.199857 | 0.964849 | 0.000006 |
| 3 | 6 | 0 | 0.000106 | 1.676310 | 0.000008 |
| 4 | 6 | 0 | –1.199722 | 0.965145 | 0.000004 |
| 5 | 6 | 0 | –1.199321 | –0.431658 | –0.000002 |
| 6 | 6 | 0 | –0.000138 | –1.143615 | –0.000004 |
| 7 | 1 | 0 | 2.118325 | 1.541499 | 0.000010 |
| 8 | 1 | 0 | –2.118301 | 1.541542 | 0.000005 |
| 9 | 1 | 0 | –0.000060 | –2.224104 | –0.000009 |
| 10 | 6 | 0 | 0.000362 | 3.234663 | 0.000013 |
| 11 | 8 | 0 | –1.135268 | 3.755517 | –0.000009 |
| 12 | 8 | 0 | 1.136370 | 3.755178 | –0.000008 |
| 13 | 6 | 0 | –2.511201 | –1.158240 | –0.000001 |

| | | | | | |
|---|---|---|---|---|---|
| 14 | 6 | 0 | 2.510993 | –1.158644 | –0.000001 |
| 15 | 9 | 0 | 3.274241 | –0.857446 | –1.084323 |
| 16 | 9 | 0 | 3.274236 | –0.857456 | 1.084329 |
| 17 | 9 | 0 | 2.370380 | –2.510815 | –0.000008 |
| 18 | 9 | 0 | –2.370993 | –2.510327 | –0.000063 |
| 19 | 9 | 0 | –3.274440 | –0.856831 | 1.084358 |
| 20 | 9 | 0 | –3.274496 | –0.856736 | –1.084293 |

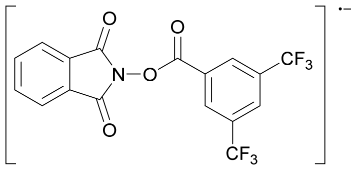

SCF Done: E(UB3LYP) = –1607.25506401 A.U.

Sum of electronic and thermal Enthalpies = –1607.017553 A.U.

| Center number | Atomic number | Atomic type | Coordinates (Angstroms) | | |
|---|---|---|---|---|---|
| | | | X | Y | Z |
| 1 | 6 | 0 | –2.903229 | 1.203707 | 0.013116 |
| 2 | 6 | 0 | –1.527029 | 1.052239 | 0.011927 |
| 3 | 6 | 0 | –0.957132 | –0.243010 | –0.006367 |
| 4 | 6 | 0 | –1.818310 | –1.365386 | –0.022506 |
| 5 | 6 | 0 | –3.190337 | –1.192799 | –0.022505 |
| 6 | 6 | 0 | –3.763605 | 0.091538 | –0.004495 |
| 7 | 1 | 0 | –0.881495 | 1.918051 | 0.025132 |
| 8 | 1 | 0 | –1.380008 | –2.353869 | –0.036651 |
| 9 | 1 | 0 | –4.837434 | 0.220389 | –0.006599 |
| 10 | 6 | 0 | 0.477632 | –0.483827 | –0.008843 |
| 11 | 8 | 0 | 1.046151 | –1.551201 | –0.023716 |
| 12 | 8 | 0 | 1.190827 | 0.738632 | 0.009029 |
| 13 | 7 | 0 | 2.548934 | 0.566320 | 0.007846 |
| 14 | 6 | 0 | 3.271751 | 0.313092 | 1.198009 |
| 15 | 8 | 0 | 2.817224 | 0.373314 | 2.327937 |
| 16 | 6 | 0 | 3.273939 | 0.347235 | –1.187709 |
| 17 | 8 | 0 | 2.821531 | 0.439766 | –2.316307 |
| 18 | 6 | 0 | 5.797116 | –0.207994 | –1.420043 |
| 19 | 6 | 0 | 4.625414 | 0.031499 | –0.705771 |
| 20 | 6 | 0 | 4.624111 | 0.011251 | 0.709737 |
| 21 | 6 | 0 | 5.794493 | –0.248641 | 1.419038 |

| | | | | | |
|---|---|---|---|---|---|
| 22 | 6 | 0 | 6.962259 | –0.497083 | 0.702432 |
| 23 | 6 | 0 | 6.963566 | –0.476867 | –0.708676 |
| 24 | 1 | 0 | 5.788821 | –0.189314 | –2.504153 |
| 25 | 1 | 0 | 5.784216 | –0.260842 | 2.503224 |
| 26 | 1 | 0 | 7.884000 | –0.711697 | 1.232781 |
| 27 | 1 | 0 | 7.886271 | –0.676287 | –1.243265 |
| 28 | 6 | 0 | –4.106404 | –2.379071 | 0.008997 |
| 29 | 6 | 0 | –3.519087 | 2.570369 | 0.010575 |
| 30 | 9 | 0 | –4.783781 | –2.495585 | 1.169509 |
| 31 | 9 | 0 | –5.066975 | –2.300829 | –0.972006 |
| 32 | 9 | 0 | –3.466953 | –3.553961 | –0.197330 |
| 33 | 9 | 0 | –4.419698 | 2.731420 | 1.020996 |
| 34 | 9 | 0 | –4.213847 | 2.820851 | –1.136376 |
| 35 | 9 | 0 | –2.618042 | 3.567852 | 0.135824 |

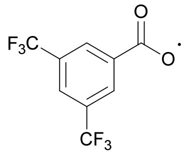


SCF Done: E(UB3LYP) = –1094.55831167 A.U.
Sum of electronic and thermal Enthalpies = –1094.435059 A.U.

| Center number | Atomic number | Atomic type | Coordinates (Angstroms) | | |
|---|---|---|---|---|---|
| | | | X | Y | Z |
| 1 | 6 | 0 | 1.204817 | –0.440453 | –0.000025 |
| 2 | 6 | 0 | 1.212040 | 0.951307 | 0.000001 |
| 3 | 6 | 0 | 0.000100 | 1.642115 | –0.000005 |
| 4 | 6 | 0 | –1.211935 | 0.951422 | –0.000030 |
| 5 | 6 | 0 | –1.204891 | –0.440312 | –0.000067 |
| 6 | 6 | 0 | –0.000068 | –1.141430 | –0.000063 |
| 7 | 1 | 0 | 2.146007 | 1.501237 | 0.000021 |
| 8 | 1 | 0 | –2.145829 | 1.501478 | –0.000041 |
| 9 | 1 | 0 | –0.000166 | –2.222683 | –0.000091 |
| 10 | 6 | 0 | 0.000214 | 3.120800 | 0.000007 |
| 11 | 8 | 0 | –1.041702 | 3.826853 | –0.000021 |
| 12 | 8 | 0 | 1.042110 | 3.826781 | 0.000024 |
| 13 | 6 | 0 | –2.525915 | –1.172106 | –0.000004 |
| 14 | 6 | 0 | 2.525777 | –1.172341 | 0.000000 |

| | | | | | |
|---|---|---|---|---|---|
| 15 | 9 | 0 | 3.265278 | –0.850992 | –0.850992 |
| 16 | 9 | 0 | 3.265142 | –0.851151 | 1.085324 |
| 17 | 9 | 0 | 2.369616 | –2.509525 | –0.000106 |
| 18 | 9 | 0 | –2.369854 | –2.509312 | –0.001071 |
| 19 | 9 | 0 | –3.264812 | –0.851608 | 1.085841 |
| 20 | 9 | 0 | –3.265826 | –0.849980 | –1.084671 |

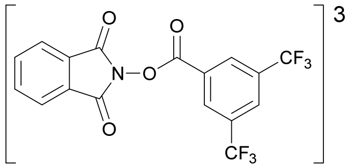


SCF Done: E(UB3LYP) = –1607.08023147 A.U.
Sum of electronic and thermal Enthalpies = –1606.842674 A.U.

| Center number | Atomic number | Atomic type | Coordinates (Angstroms) | | |
|---|---|---|---|---|---|
| | | | X | Y | Z |
| 1 | 6 | 0 | –2.845578 | 1.251859 | 0.024785 |
| 2 | 6 | 0 | –1.477799 | 1.008466 | –0.016503 |
| 3 | 6 | 0 | –1.015291 | –0.312190 | –0.013154 |
| 4 | 6 | 0 | –1.919400 | –1.373810 | –0.015855 |
| 5 | 6 | 0 | –3.288065 | –1.114654 | –0.022079 |
| 6 | 6 | 0 | –3.757403 | 0.194681 | –0.024886 |
| 7 | 1 | 0 | –0.776773 | 1.830567 | 0.019526 |
| 8 | 1 | 0 | –1.542550 | –2.388966 | –0.019272 |
| 9 | 1 | 0 | –4.821681 | 0.390304 | –0.037766 |
| 10 | 6 | 0 | 0.431357 | –0.652229 | –0.009685 |
| 11 | 8 | 0 | 0.908647 | –1.745151 | –0.005591 |
| 12 | 8 | 0 | 1.201257 | 0.517889 | –0.011055 |
| 13 | 7 | 0 | 2.544496 | 0.296820 | –0.005755 |
| 14 | 6 | 0 | 3.271907 | 0.191655 | 1.220978 |
| 15 | 8 | 0 | 2.782901 | 0.250848 | 2.330694 |
| 16 | 6 | 0 | 3.280336 | 0.181353 | –1.226513 |
| 17 | 8 | 0 | 2.799085 | 0.231184 | –2.340066 |
| 18 | 6 | 0 | 5.831034 | –0.171238 | –1.452994 |
| 19 | 6 | 0 | 4.633512 | 0.002905 | –0.748939 |
| 20 | 6 | 0 | 4.628322 | 0.009237 | 0.754322 |
| 21 | 6 | 0 | 5.820882 | –0.158940 | 1.468186 |
| 22 | 6 | 0 | 6.983852 | –0.324418 | 0.757114 |

| | | | | | |
|---|---|---|---|---|---|
| 23 | 6 | 0 | 6.989013 | –0.330724 | –0.732440 |
| 24 | 1 | 0 | 5.829113 | –0.178024 | –2.536265 |
| 25 | 1 | 0 | 5.811351 | –0.156624 | 2.551437 |
| 26 | 1 | 0 | 7.928522 | –0.455991 | 1.269967 |
| 27 | 1 | 0 | 7.937219 | –0.466643 | –1.237581 |
| 28 | 6 | 0 | –4.251966 | –2.275701 | 0.004781 |
| 29 | 6 | 0 | –3.373977 | 2.665054 | –0.002319 |
| 30 | 9 | 0 | –4.262885 | –2.879869 | 1.215933 |
| 31 | 9 | 0 | –5.517491 | –1.893418 | –0.259835 |
| 32 | 9 | 0 | –3.916863 | –3.221455 | –0.899260 |
| 33 | 9 | 0 | –3.888169 | 2.980694 | 1.210247 |
| 34 | 9 | 0 | –4.368582 | 2.836423 | –0.901695 |
| 35 | 9 | 0 | –2.418920 | 3.573363 | 0.278051 |

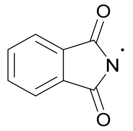

SCF Done: E(UB3LYP) = –512.535994605 A.U.

Sum of electronic and thermal Enthalpies = –512.426115 A.U.

| Center number | Atomic number | Atomic type | Coordinates (Angstroms) | | |
|---|---|---|---|---|---|
| | | | X | Y | Z |
| 1 | 7 | 0 | –0.260611 | –2.087863 | 0.000000 |
| 2 | 6 | 0 | –0.014265 | –1.279611 | 1.150523 |
| 3 | 8 | 0 | 0.141508 | –1.718136 | –2.264339 |
| 4 | 6 | 0 | –0.014265 | –1.279611 | –1.150523 |
| 5 | 8 | 0 | 0.141508 | –1.718136 | –2.264339 |
| 6 | 6 | 0 | –0.010030 | 1.327790 | –1.425467 |
| 7 | 6 | 0 | –0.012316 | 0.143196 | 0.698900 |
| 8 | 6 | 0 | –0.012316 | 0.143196 | 0.698900 |
| 9 | 6 | 0 | –0.010030 | 1.327790 | 1.425467 |
| 10 | 6 | 0 | –0.000120 | 2.519874 | 0.702726 |
| 11 | 6 | 0 | –0.000120 | 2.519874 | –0.702726 |
| 12 | 1 | 0 | –0.010152 | 1.318335 | –2.508647 |
| 13 | 1 | 0 | –0.010152 | 1.318335 | 2.508647 |
| 14 | 1 | 0 | 0.010610 | 3.466785 | 1.230114 |
| 15 | 1 | 0 | 0.010610 | 3.466785 | –1.230114 |

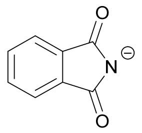


SCF Done: E(RB3LYP) = –512.683427930 A.U.
Sum of electronic and thermal Enthalpies = –512.572998 A.U.

| Center number | Atomic number | Atomic type | Coordinates (Angstroms) | | |
|---|---|---|---|---|---|
| | | | X | Y | Z |
| 1 | 6 | 0 | –0.000040 | 2.531187 | 0.699222 |
| 2 | 6 | 0 | –0.000040 | 1.325447 | 1.416329 |
| 3 | 6 | 0 | –0.000040 | 0.142907 | 0.694117 |
| 4 | 6 | 0 | –0.000040 | 0.142907 | –0.694117 |
| 5 | 6 | 0 | –0.000040 | 1.325447 | –1.416329 |
| 6 | 6 | 0 | –0.000040 | 2.531187 | –0.699222 |
| 7 | 1 | 0 | –0.000040 | 3.477897 | 1.231998 |
| 8 | 1 | 0 | –0.000039 | 1.311147 | 2.501446 |
| 9 | 1 | 0 | –0.000039 | 1.311147 | –2.501446 |
| 10 | 1 | 0 | –0.000040 | 3.477897 | –1.231998 |
| 11 | 6 | 0 | –0.000040 | –1.324702 | –1.113330 |
| 12 | 8 | 0 | 0.000102 | –1.679948 | –2.293406 |
| 13 | 6 | 0 | –0.000040 | 1.324702 | 1.113330 |
| 14 | 8 | 0 | 0.000102 | –1.679948 | 2.293406 |
| 15 | 7 | 0 | 0.000063 | –2.113857 | 0.000000 |

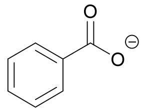


SCF Done: E(RB3LYP) = –420.396698283 A.U.
Sum of electronic and thermal Enthalpies = –420.287472 A.U.

| Center number | Atomic number | Atomic type | Coordinates (Angstroms) | | |
|---|---|---|---|---|---|
| | | | X | Y | Z |
| 1 | 6 | 0 | 1.833028 | 1.205596 | –0.000001 |
| 2 | 6 | 0 | 0.437804 | 1.201215 | 0.000009 |
| 3 | 6 | 0 | –0.279020 | –0.000042 | 0.000013 |
| 4 | 6 | 0 | 0.437919 | –1.201237 | 0.000006 |
| 5 | 6 | 0 | 1.833153 | –1.205519 | –0.000004 |
| 6 | 6 | 0 | 2.538243 | 0.000053 | –0.000008 |

| | | | | | |
|---|---|---|---|---|---|
| 7 | 1 | 0 | 2.374163 | 2.148358 | –0.000004 |
| 8 | 1 | 0 | –0.135447 | 2.121713 | 0.000013 |
| 9 | 1 | 0 | –0.135202 | –2.121801 | 0.000009 |
| 10 | 1 | 0 | 2.374306 | –2.148269 | –0.000009 |
| 11 | 1 | 0 | 3.624618 | 0.000157 | –0.000016 |
| 12 | 6 | 0 | –1.833938 | –0.000014 | 0.000022 |
| 13 | 8 | 0 | –2.369034 | –1.133462 | –0.000010 |
| 14 | 8 | 0 | –2.369162 | 1.133404 | –0.000016 |

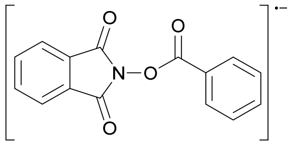

SCF Done: E(UB3LYP) = –932.947939500 A.U.
Sum of electronic and thermal Enthalpies = –932.725129 A.U.

| Center number | Atomic number | Atomic type | Coordinates (Angstroms) | | |
|---|---|---|---|---|---|
| | | | X | Y | Z |
| 1 | 6 | 0 | –4.640704 | 0.000138 | –1.506624 |
| 2 | 6 | 0 | –3.294158 | 0.000073 | –1.150556 |
| 3 | 6 | 0 | –2.929455 | –0.000045 | 0.200290 |
| 4 | 6 | 0 | –3.922470 | –0.000101 | 1.184820 |
| 5 | 6 | 0 | –5.266655 | –0.000036 | 0.825674 |
| 6 | 6 | 0 | –5.628300 | 0.000085 | –0.521943 |
| 7 | 1 | 0 | –4.919032 | 0.000228 | –2.554911 |
| 8 | 1 | 0 | –2.522677 | 0.000110 | –1.908519 |
| 9 | 1 | 0 | –3.617808 | –0.000194 | 2.224273 |
| 10 | 1 | 0 | –6.031819 | –0.000079 | 1.594478 |
| 11 | 1 | 0 | –6.676184 | 0.000136 | –0.803562 |
| 12 | 6 | 0 | –1.501082 | –0.000115 | 0.653299 |
| 13 | 8 | 0 | –1.150268 | –0.000274 | 1.800824 |
| 14 | 8 | 0 | –0.667736 | 0.000087 | –0.429111 |
| 15 | 7 | 0 | 0.682410 | 0.000049 | –0.129870 |
| 16 | 6 | 0 | 1.438048 | 1.208153 | –0.158492 |
| 17 | 8 | 0 | 0.942737 | 2.338966 | –0.226894 |
| 18 | 6 | 0 | 1.438016 | –1.208073 | –0.158691 |
| 19 | 8 | 0 | 0.942663 | –2.338862 | –0.227175 |

| | | | | | |
|---|---|---|---|---|---|
| 20 | 6 | 0 | 4.008173 | –1.421226 | –0.033241 |
| 21 | 6 | 0 | 2.794298 | –0.720150 | –0.088917 |
| 22 | 6 | 0 | 2.794320 | 0.720178 | –0.088840 |
| 23 | 6 | 0 | 4.008215 | 1.421211 | –0.033097 |
| 24 | 6 | 0 | 5.197571 | 0.712641 | 0.018239 |
| 25 | 6 | 0 | 5.197550 | –0.712696 | 0.018168 |
| 26 | 1 | 0 | 4.002822 | –2.506627 | –0.032729 |
| 27 | 1 | 0 | 4.002895 | 2.506612 | –0.032450 |
| 28 | 1 | 0 | 6.142803 | 1.245081 | 0.060482 |
| 29 | 1 | 0 | 6.142766 | –1.245169 | 0.060349 |

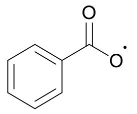

SCF Done: E(UB3LYP) = –420.269145039 A.U.
Sum of electronic and thermal Enthalpies = –420.159778 A.U.

| Center number | Atomic number | Atomic type | Coordinates (Angstroms) | | |
|---|---|---|---|---|---|
| | | | X | Y | Z |
| 1 | 6 | 0 | 1.837379 | 1.211198 | –0.000131 |
| 2 | 6 | 0 | 0.446865 | 1.214539 | 0.000022 |
| 3 | 6 | 0 | –0.247221 | –0.000090 | 0.000191 |
| 4 | 6 | 0 | 0.447040 | –1.214639 | 0.000182 |
| 5 | 6 | 0 | 1.837455 | –1.211278 | 0.000037 |
| 6 | 6 | 0 | 2.530853 | 0.000022 | –0.000121 |
| 7 | 1 | 0 | 2.380981 | 2.148586 | –0.000267 |
| 8 | 1 | 0 | –0.105829 | 2.146911 | 0.000009 |
| 9 | 1 | 0 | –0.105892 | –2.146897 | 0.000317 |
| 10 | 1 | 0 | 2.381133 | –2.148612 | 0.000061 |
| 11 | 1 | 0 | 3.615066 | 0.000239 | –0.000239 |
| 12 | 6 | 0 | –1.719439 | 0.000018 | 0.000386 |
| 13 | 8 | 0 | –2.435536 | –1.039408 | –0.000431 |
| 14 | 8 | 0 | –2.434846 | 1.039569 | 0.000021 |

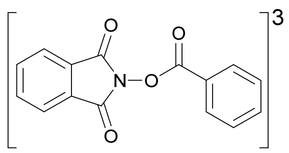

SCF Done: E(UB3LYP) = –932.788727182 A.U.
Sum of electronic and thermal Energies = –932.568131 A.U.

| Center number | Atomic number | Atomic type | Coordinates (Angstroms) | | |
|---|---|---|---|---|---|
| | | | X | Y | Z |
| 1 | 6 | 0 | –4.723575 | 0.000028 | –1.348159 |
| 2 | 6 | 0 | –3.353730 | –0.000029 | –1.106252 |
| 3 | 6 | 0 | –2.882581 | –0.000045 | 0.212535 |
| 4 | 6 | 0 | –3.787921 | –0.000002 | –1.281451 |
| 5 | 6 | 0 | –5.154810 | 0.000055 | 1.031663 |
| 6 | 6 | 0 | –5.623384 | 0.000070 | –0.282704 |
| 7 | 1 | 0 | –5.089056 | 0.000041 | –2.368326 |
| 8 | 1 | 0 | –2.653222 | –0.000062 | –1.930130 |
| 9 | 1 | 0 | –3.404772 | –0.000014 | 2.294242 |
| 10 | 1 | 0 | –5.854717 | 0.000087 | 1.859041 |
| 11 | 1 | 0 | –6.690219 | 0.000114 | –0.476252 |
| 12 | 6 | 0 | –1.442082 | –0.000108 | 0.545318 |
| 13 | 8 | 0 | –0.940357 | –1.628040 | 1.628040 |
| 14 | 8 | 0 | –0.664293 | –0.000045 | –0.638832 |
| 15 | 7 | 0 | 0.673522 | –0.000023 | –0.420605 |
| 16 | 6 | 0 | 1.407716 | 1.223915 | –0.305973 |
| 17 | 8 | 0 | 0.924562 | 2.336305 | –0.366359 |
| 18 | 6 | 0 | 1.407759 | –1.223935 | –0.305977 |
| 19 | 8 | 0 | 0.924641 | –2.336341 | –0.366359 |
| 20 | 6 | 0 | 3.954381 | –1.458866 | 0.066570 |
| 21 | 6 | 0 | 2.759447 | –0.750445 | –0.115874 |
| 22 | 6 | 0 | 2.759421 | 0.750471 | –0.115873 |
| 23 | 6 | 0 | 3.954329 | 1.458933 | 0.066581 |
| 24 | 6 | 0 | 5.113195 | 0.743785 | 0.239334 |
| 25 | 6 | 0 | 5.113222 | –0.743677 | 0.239327 |
| 26 | 1 | 0 | 3.948257 | –2.542187 | 0.069304 |
| 27 | 1 | 0 | 3.948167 | 2.542254 | 0.069324 |
| 28 | 1 | 0 | 6.058058 | 1.253567 | 0.381665 |
| 29 | 1 | 0 | 6.058103 | –1.253428 | 0.381653 |

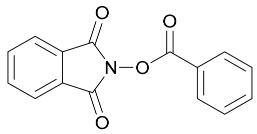


SCF Done: E(RB3LYP) = –932.899971218 A.U.

after Total Energy, E(TD-HF/TD-KS) = –932.781254851

Excited State 1: Triplet-A 3.2304 eV 383.80 nm f=0.0000 <S**2> = 2.000

| Center number | Atomic number | Atomic type | Coordinates (Angstroms) | | |
|---|---|---|---|---|---|
| | | | X | Y | Z |
| 1 | 6 | 0 | 4.781414 | –0.001016 | –1.301049 |
| 2 | 6 | 0 | 3.404735 | –0.000883 | –1.100992 |
| 3 | 6 | 0 | 2.892966 | 0.000159 | 0.202963 |
| 4 | 6 | 0 | 3.765837 | 0.001044 | 1.298497 |
| 5 | 6 | 0 | 5.139748 | 0.001007 | 1.090708 |
| 6 | 6 | 0 | 5.648378 | –0.000032 | –0.208721 |
| 7 | 1 | 0 | 5.177866 | –0.001916 | –2.309609 |
| 8 | 1 | 0 | 2.729890 | –0.001601 | –1.945999 |
| 9 | 1 | 0 | 3.351854 | 0.001776 | 2.299082 |
| 10 | 1 | 0 | 5.814158 | 0.001789 | 1.939014 |
| 11 | 1 | 0 | 6.720620 | –0.000128 | –0.369627 |
| 12 | 6 | 0 | 1.441721 | 0.000174 | 0.491456 |
| 13 | 8 | 0 | 0.913893 | 0.000550 | 1.564330 |
| 14 | 8 | 0 | 0.699079 | –0.000546 | –0.704806 |
| 15 | 7 | 0 | –0.653574 | –0.000336 | –0.498821 |
| 16 | 6 | 0 | –1.395450 | –1.187868 | –0.332911 |
| 17 | 8 | 0 | –0.956455 | –2.306339 | –0.380180 |
| 18 | 6 | 0 | –1.395252 | 1.187400 | –0.333775 |
| 19 | 8 | 0 | –0.956062 | 2.305771 | –0.381781 |
| 20 | 6 | 0 | –3.952927 | 1.420936 | 0.086984 |
| 21 | 6 | 0 | –2.787706 | 0.698533 | –0.107398 |
| 22 | 6 | 0 | –2.787801 | –0.698581 | –0.106772 |
| 23 | 6 | 0 | –3.953136 | –1.420617 | 0.088344 |
| 24 | 6 | 0 | –5.135137 | –0.698436 | 0.286252 |
| 25 | 6 | 0 | –5.135046 | 0.699124 | 0.285550 |
| 26 | 1 | 0 | –3.942139 | 2.504103 | 0.086799 |
| 27 | 1 | 0 | –3.942569 | –2.503788 | 0.089063 |
| 28 | 1 | 0 | –6.066271 | –1.230106 | 0.444450 |
| 29 | 1 | 0 | –6.066103 | 1.231072 | 0.443259 |

## N. Synthesis and Characterization of Aryl-Acylperoxide:

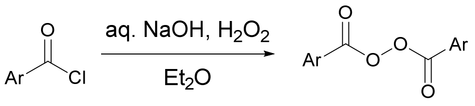


The aryl-acylperoxides were prepared by following the modified literature procedure.[16] In a 100 mL round bottom flask equipped with magnetic stir bar, a solution of the acid chloride (7.3 mmol) in $Et_2O$ (3.0 mL) was cooled to 0 °C. $H_2O_2$ (aq. 30% by wt., 0.5 mL) was added dropwise followed by the dropwise addition of an aqueous solution of NaOH (364.0 mg in 2.0 mL $H_2O$) over 20 minutes. The reaction was quenched by adding a saturated aqueous solution of $NaHCO_3$ (15 mL). The resulting solid was filtered and the aqueous phase was extracted with $Et_2O$ (2 × 20 mL). The filtered solid was mixed with the organic phase, dried over anhydrous $Na_2SO_4$ and concentrated under reduced pressure at low temperature to afford a white solid. Recrystallization from hexane yielded the aryl-acylperoxides as a white solid.

**4**: Following the general procedure described above with 2,3,4,5,6-pentafluorobenzoyl chloride, **4** was obtained as a white solid (502 mg, 1.18 mmol, 16% yield), which was stored at –40 °C. **$^{13}$C-NMR (150 MHz, CDCl$_3$)**: δ 154.9, 147.0-146.6 (m), 146.0-145.6 (m), 145.3-144.9 (m), 144.2-143.8 (m), 139.1-138.5 (m), 137.4-136.8 (m), 102.58 (t, $J$ = 17.2 Hz). **$^{19}$F-NMR (471 MHz, CDCl$_3$)**: δ –158.104, –143.278, –133.679.

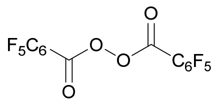

Following the general procedure described above with 3,5-bis-(trifluoromethyl)benzoyl chloride, the corresponding peroxide was obtained as a white solid (1.4 g, 2.72 mmol, 38% yield), which was stored at –40 °C. **$^{1}$H-NMR (400 MHz, CDCl$_3$)**: δ 8.53 (s, 2H), 8.21 (s, 1H). **$^{13}$C-NMR (100 MHz, CDCl$_3$)**: δ 160.4, 133.1 (q, $J$ = 69 Hz), 130.0, 128.1, 127.4, 122.5 (q, $J$ = 69 Hz), 123.9, 121.2. **$^{19}$F-NMR (471 MHz, CDCl$_3$)**: δ –62.97.

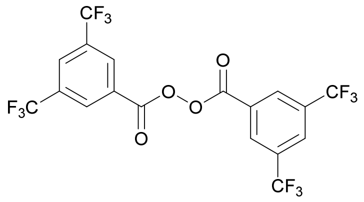

## O. Reaction Conditions Optimization for the Ketone-Catalyzed C-H Acyloxylation:

### ***Effect of Oxygenating Agent***

Considering our results for C-H imidation of arenes, we thought that the aryl-acylperoxides with electron-withdrawing groups would be suitable for the C-H acyloxylation of arenes. Among the several candidates examined in the reaction of benzene, pentafluorobenzoyl peroxide led to afford the acyloxylation product in the highest yield.

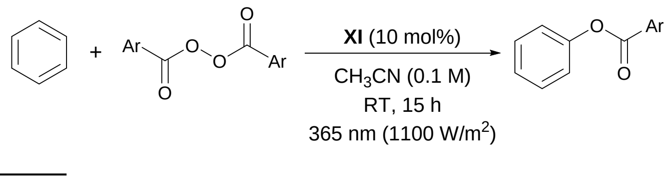


16. W.-Y. Yu, W. N. Sit, Z. Zhou and A. S. C. Chan, *Org. Lett.*, 2009, **11**, 3174.

| Entry | Ar | Yield (%) |
|---|---|---|
| 1 | $C_6H_5$ | <5 |
| 2 | 4-$CF_3C_6H_4$ | 6 |
| 3 | 3,5-$(CF_3)_2C_6H_3$ | 20 |
| 4 | $C_6F_5$ (**4**) | 25 |

(a) Reactions were carried out on a 0.2 mmol scale with oxygenating agent (1.0 equiv) and arenes (10.0 equiv). (b) NMR yields are indicated.

## ***Effect of Light Intensity***

During optimization of the reaction conditions, we observed that 365 nm light with high intensity caused the decomposition of **4**. Therefore, we reasoned that decreasing the light intensity might be beneficial for improving the efficiency. After carefully conducting the C-H acyloxylation with different intensity of 365 nm light, we concluded that 325 $W/m^2$ was an optimum intensity.

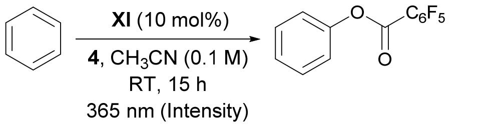


| Intensity ($W/m^2$): | 1100 | 512 | 325 | 200 | 100 |
|---|---|---|---|---|---|
| Yield (%): | 25 | 36 | 43 | 34 | 34 |

(a) Reactions were carried out on a 0.2 mmol scale with **4** (1.0 equiv) and arenes (10.0 equiv). (b) NMR yields are indicated.

## ***Catalyst Optimization***

A brief catalyst screening revealed that **X** was optimal and the product was obtained in 63% yield.

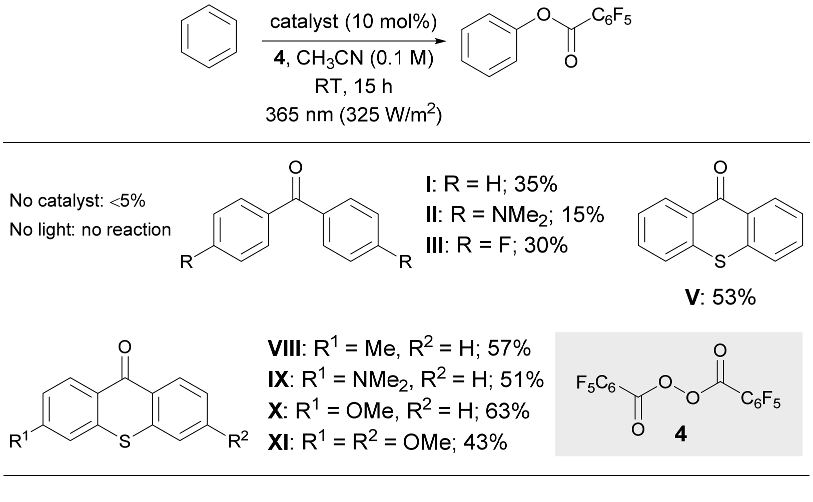


(a) Reactions were carried out on a 0.2 mmol scale with **4** (1.0 equiv) and arenes (10.0 equiv). (b) NMR yields are indicated.

## ***Effect of Solvent***

The reaction yield greatly depended upon the solvents and the best result was obtained by using a 1:1 mixture of toluene and 1,2-dichloroethane.

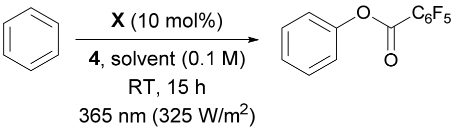


| Solvent: | $CH_3CN$ | $CH_2Cl_2$ | THF | DCE | $CH_3CN$/DCE (1:1) |
|---|---|---|---|---|---|
| Yield (%): | 63 | 51 | <5 | 57 | 80 (66% isolated yield) |

(a) Reactions were carried out on a 0.1 mmol scale with **4** (1.0 equiv) and arenes (10.0 equiv). (b) NMR yields are indicated.

## P. Photoexcited Ketone-Catalyzed C-H Acyloxylation of Arenes:

### General procedure for the ketone-catalyzed C-H acyloxylation of arenes:

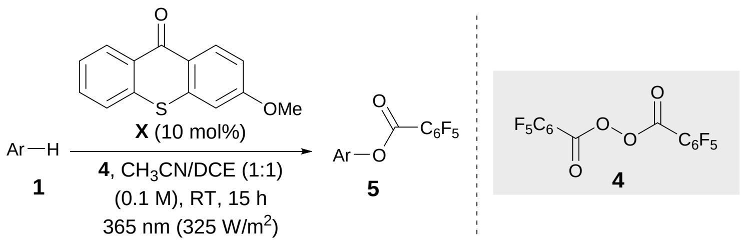


In a flame and vacuum dried reaction tube, **4** (84.4 mg, 0.2 mmol, 1.0 equiv) and **X** (5.0 mg, 0.02 mmol, 0.1 equiv) were taken. The reaction flask was degassed in *vacuo* and backfilled with argon. Then, $CH_3CN$/DCE (2.0 mL, v/v = 1:1) and arene (2.0 mmol, 10.0 equiv) were added. The resulting solution was stirred at 25 °C under 365 nm irradiation (325 W/m$^2$) for 15 h. The reaction was quenched by adding a saturated aqueous solution of $NaHCO_3$ (5 mL). The aqueous phase was extracted with $CH_2Cl_2$ (3 × 10 mL). The combined organic phase was dried over anhydrous $Na_2SO_4$ and concentrated under reduced pressure. Purification of the residue by column chromatography over silica gel afforded **5**.

**5a**: Following the general procedure described above with benzene (0.18 mL, 2.0 mmol, 10.0 equiv) as

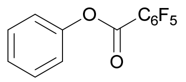


the arene source, **5a** was obtained as a white solid (38.0 mg, 0.13 mmol, 66% yield). **FT-IR (thin film)**: 1751, 1552, 1489, 1234. **$^1$H-NMR (600 MHz, $CDCl_3$)**: δ 7.47-7.41 (m, 2H), 7.79-7.76 (m, 6.5H), 7.33-7.28 (m, 1H), 7.26-7.21 (m, 2H). **$^{13}$C-NMR (150 MHz, $CDCl_3$)**: δ 157.6, 150.1, 146.6, 144.8, 144.6, 142.9, 138.7, 137.0, 129.8, 126.8, 121.3, 107.8. **$^{19}$F-NMR (471 MHz, $CDCl_3$)**: δ –159.78, –147.16, –137.13. **HRMS (APCI-MS)**: Calcd. for $C_{13}H_5F_5O_2H$ ([M + H]$^+$): 289.0282, Found: 289.0282.

**5b**: Following the general procedure described above with naphthalene (256.3 mg, 2.0 mmol, 10.0 equiv) as the arene source, **5b** was obtained as a white solid (56 mg, 0.165 mmol, 82% yield, only *ortho*). The isomer ratios were determined by hydrolysis of the product to the corresponding phenol

derivatives and comparing the crude $^{1}$H-NMR with literature data. **FT-IR (thin film)**: 1745, 1498, 1325. **$^{1}$H-NMR (400 MHz, CDCl$_3$)**: δ 8.02-7.96 (m, 1H), 7.95-7.90 (m, 1H), 7.83 (d, *J* = 8.4 Hz, 1H), 7.59-7.48 (m, 3H), 7.43 (dd, *J* = 7.6, 1.0 Hz, 1H). **$^{13}$C-NMR (150 MHz, CDCl$_3$)**: δ 157.6, 146.7, 146.0, 145.0, 144.7, 142.8, 138.8, 137.2, 134.8, 128.2, 127.08, 127.03, 126.9, 126.2, 125.4, 120.9, 118.0, 107.8 (td, *J* = 16.2, 3 Hz). **$^{19}$F-NMR (471 MHz, CDCl$_3$)**: δ –159.51 (t, *J* = 29 Hz), –146.94 (t, *J* = 29 Hz), –136.79 (d, *J* = 29 Hz). **HRMS (APCI-MS)**: Calcd. for $C_{17}H_7F_5O_2H$ ([M + H]$^+$): 339.0437, Found: 339.0439.

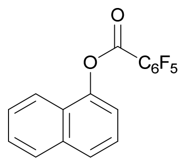


**5c**: Following the general procedure described above with toluene (0.21 mL, 2.0 mmol, 10.0 equiv) as the arene source, **5c** was obtained as a white solid (43 mg, 0.14 mmol, 71% yield, *o*/*m*/*p* = 4.5:1:1.8). The isomer ratios were determined after hydrolysis of the product to the corresponding phenol derivatives and comparing the crude $^{1}$H-NMR with literature data. The isolated product was an inseparable mixture of isomers. All peaks observed in the $^{1}$H-NMR and $^{13}$C-NMR were assigned. **FT-IR (thin film)**: 1757, 1524, 1500, 1329. **$^{1}$H-NMR (600 MHz, CDCl$_3$)**: δ 7.34-7.18 (m, 7.3H), 7.16-7.07 (m, 3H), 7.05-7.00 (m, 0.7H), 2.39 (s, 1H), 2.37 (s, 1.8H), 2.26 (s, 4.5H). **$^{13}$C-NMR (150 MHz, CDCl$_3$)**: δ 148.8, 136.6, 131.6, 130.3, 129.9, 129.5, 127.6, 127.3, 127.0, 121.8, 121.6, 120.9, 118.2, 21.4, 21.0, 16.3. **$^{19}$F-NMR (471 MHz, CDCl$_3$)**: δ –159.78, –147.37, –137.22. **HRMS (APCI-MS)**: Calcd. for $C_{14}H_7F_5O_2H$ ([M + H]$^+$): 303.0439, Found: 303.0439.

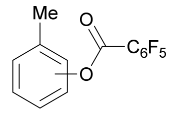


**5d**: Following the general procedure described above with chlorobenzene (0.20 mL, 2.0 mmol, 10.0 equiv) as the arene source, **5d** was obtained as a white solid (27 mg, 0.083 mmol, 42% yield, *o*/*m*/*p* = 2.4:1:3.3). The isomer ratios were determined after hydrolysis of the product to the corresponding phenol derivatives and comparing the crude $^{1}$H-NMR with literature data. The isolated product was an inseparable mixture of isomers. All peaks observed in the $^{1}$H-NMR and $^{13}$C-NMR were assigned. **FT-IR (thin film)**: 1753, 1525, 1330. **$^{1}$H-NMR (600 MHz, CDCl$_3$)**: δ 7.53-7.36 (m, 2H), 7.36-7.22 (m, 2H), 7.22-7.14 (m, 1H). **$^{13}$C-NMR (150 MHz, CDCl$_3$)**: δ 148.5, 146.3, 132.3, 130.7, 130.5, 129.9, 129.8, 128.1, 128.0, 127.2, 126.8, 126.7, 123.5, 122.7, 122.1, 121.3, 119.7. **$^{19}$F-NMR (471 MHz, CDCl$_3$)**: δ –159.66, –147.19, –146.49, –137.19, –136.88, –136.06. **HRMS (APCI-MS)**: Calcd. for $C_{13}H_4ClF_5O_2H$ ([M + H]$^+$): 322.9893, Found: 322.9893.

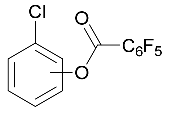


**General procedure for the hydrolysis of 5:** In a 10 mL round bottom flask, a solution of **5** (0.15 mmol) in EtOH (0.3 mL) was taken and a 2N aqueous solution of NaOH (0.3 mL) was added. The resulting mixture was stirred at 25 °C for 15 h. The reaction mixture was cooled to 0 °C and acidified with a 1N aqueous solution of HCl. The aqueous phase was extracted with EtOAc (× 2). The organic phase was dried over anhydrous $Na_2SO_4$ and concentrated under reduced pressure. Purification of the residue by column chromatography over silica gel afforded corresponding phenol derivatives.

## Q. Copies of NMR Spectra:

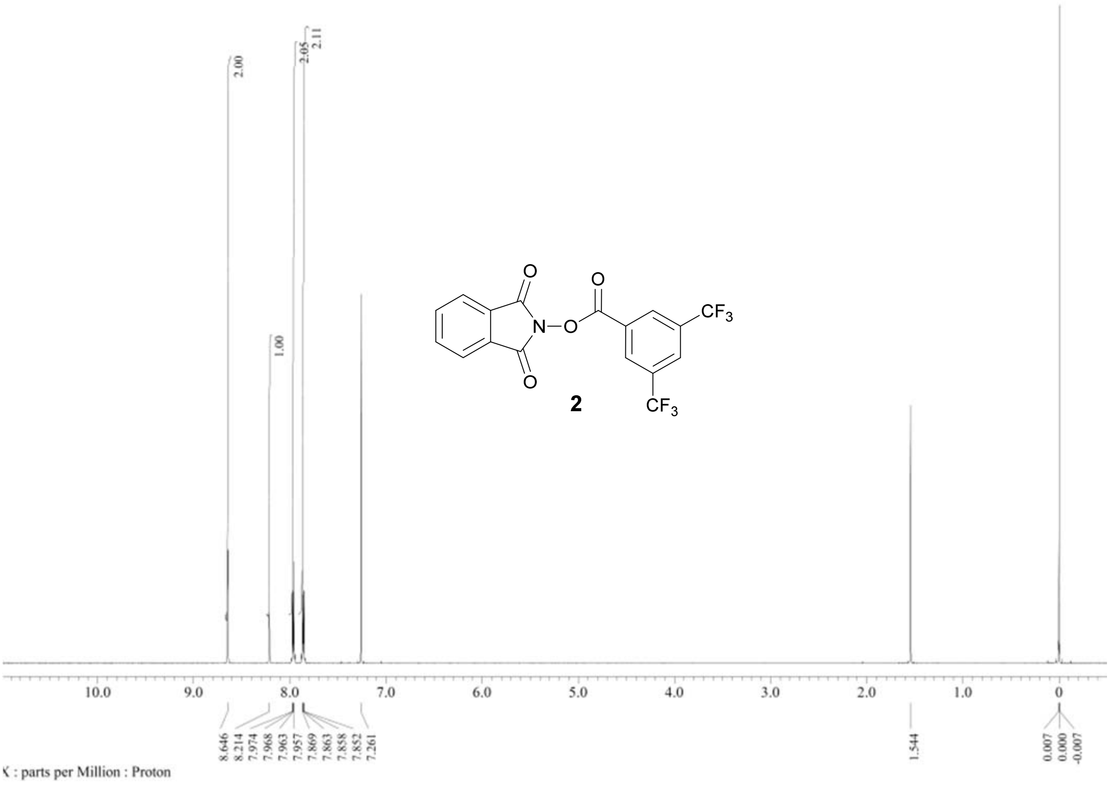


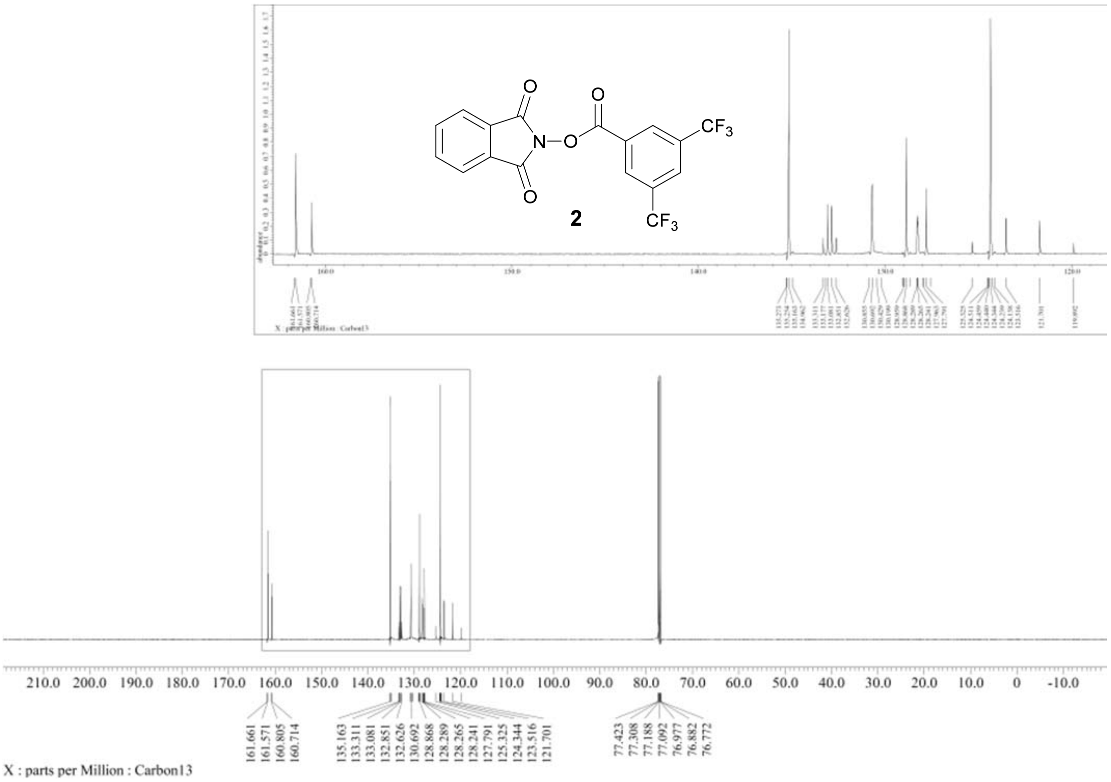

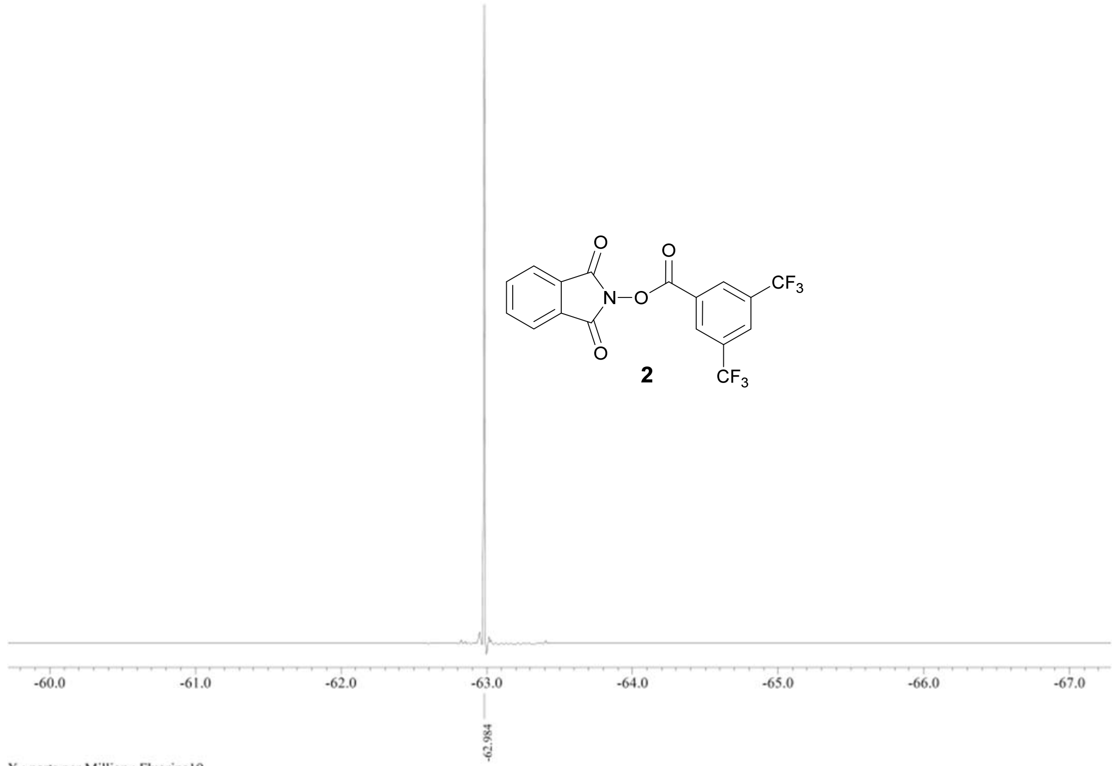
2
-62.984
X : parts per Million : Fluorine19

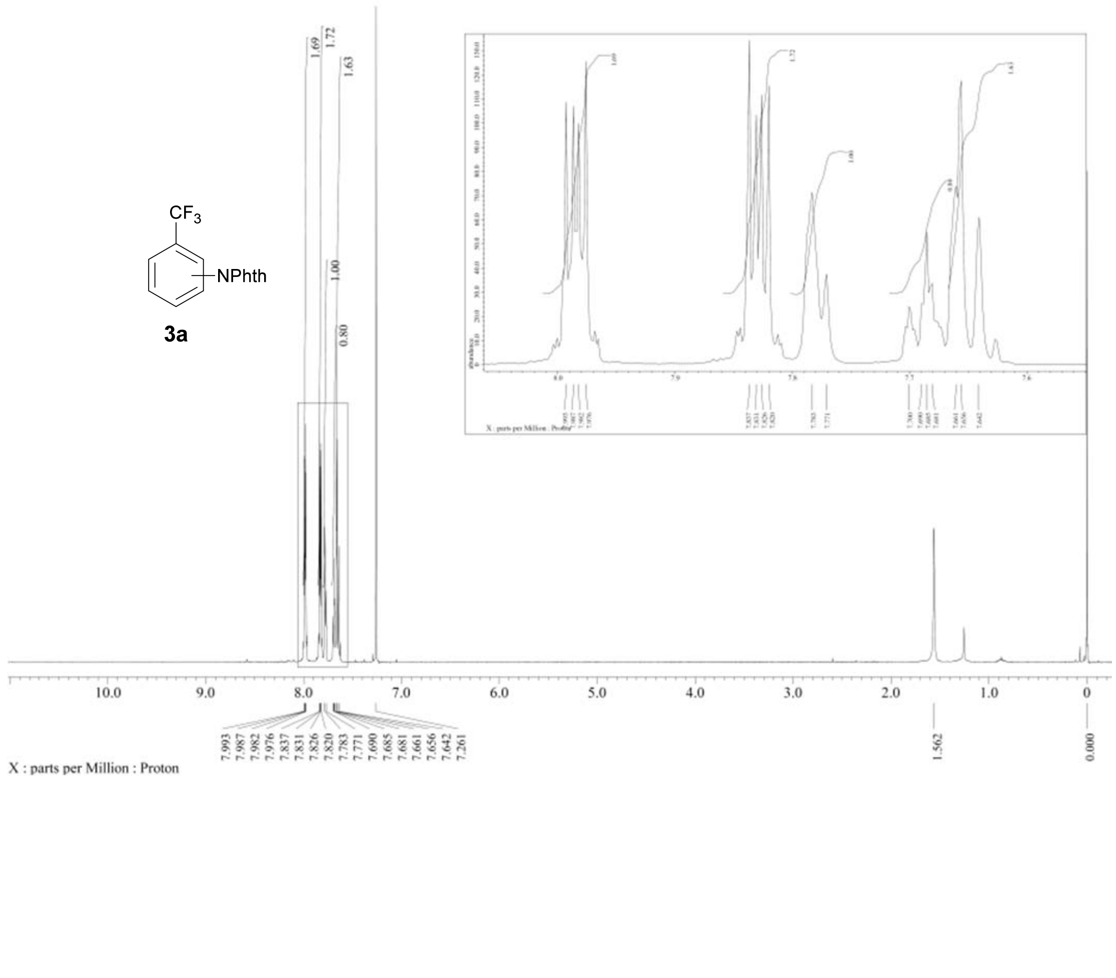
3a
NPhth
1.69
1.72
1.63
1.00
0.80
7.993
7.987
7.982
7.976
7.837
7.831
7.826
7.820
7.783
7.771
7.690
7.685
7.681
7.661
7.656
7.642
7.261
1.562
0.000
X : parts per Million : Proton

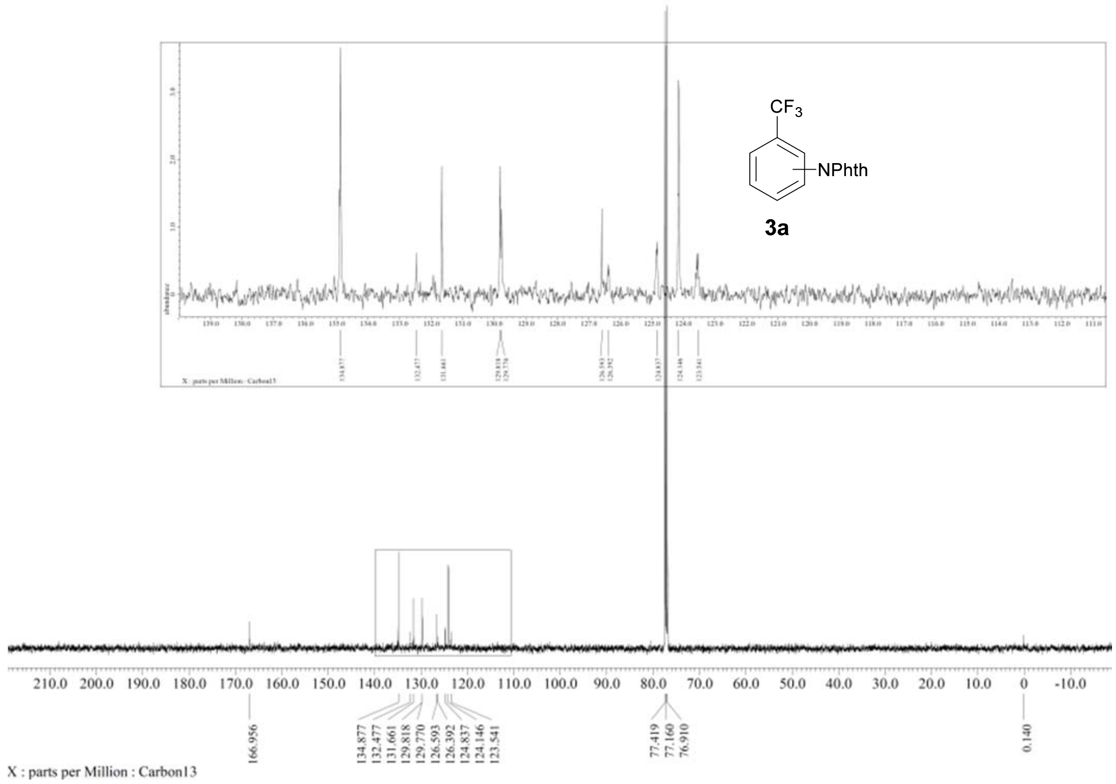
CF3
NPhth
3a
X : parts per Million : Carbon13
166.956
134.877
132.477
131.661
129.818
129.770
126.593
126.392
124.837
124.146
123.541
77.419
77.160
76.910
0.140

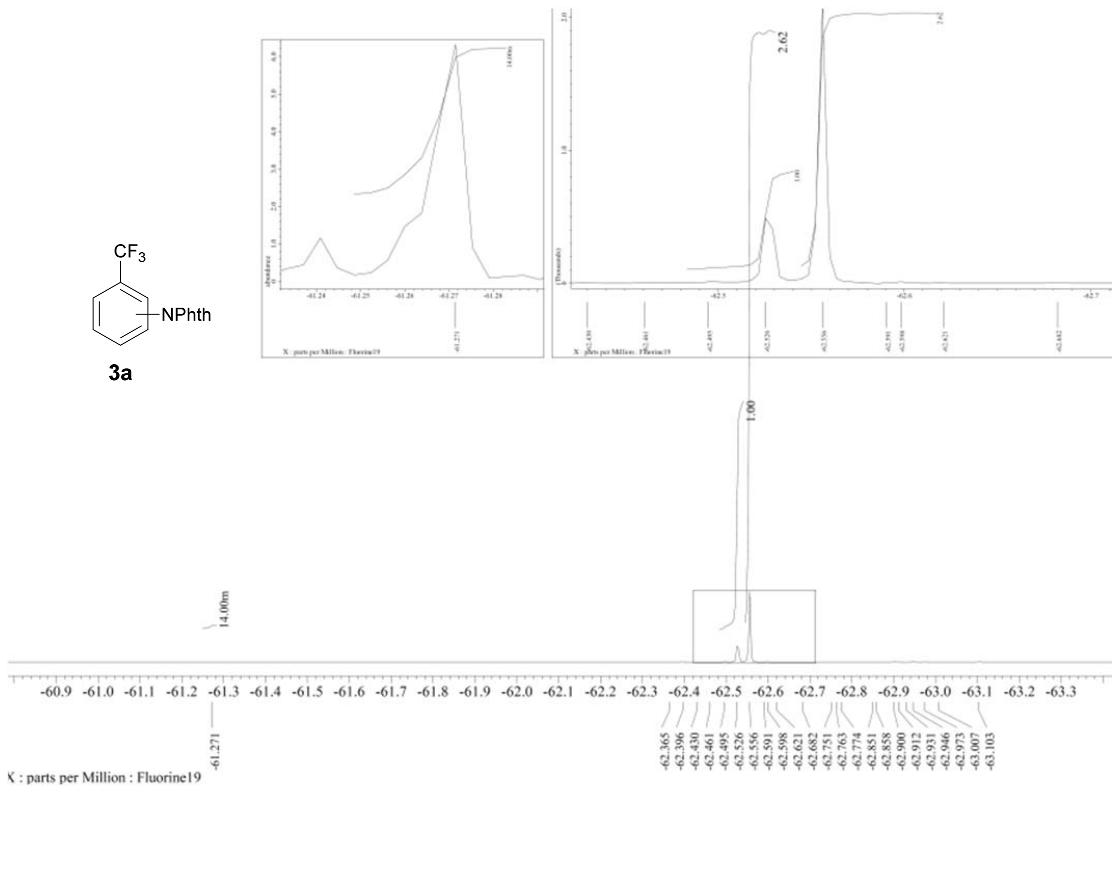
CF3
NPhth
3a
2.62
1.00
14.00m
X : parts per Million : Fluorine19
-61.271

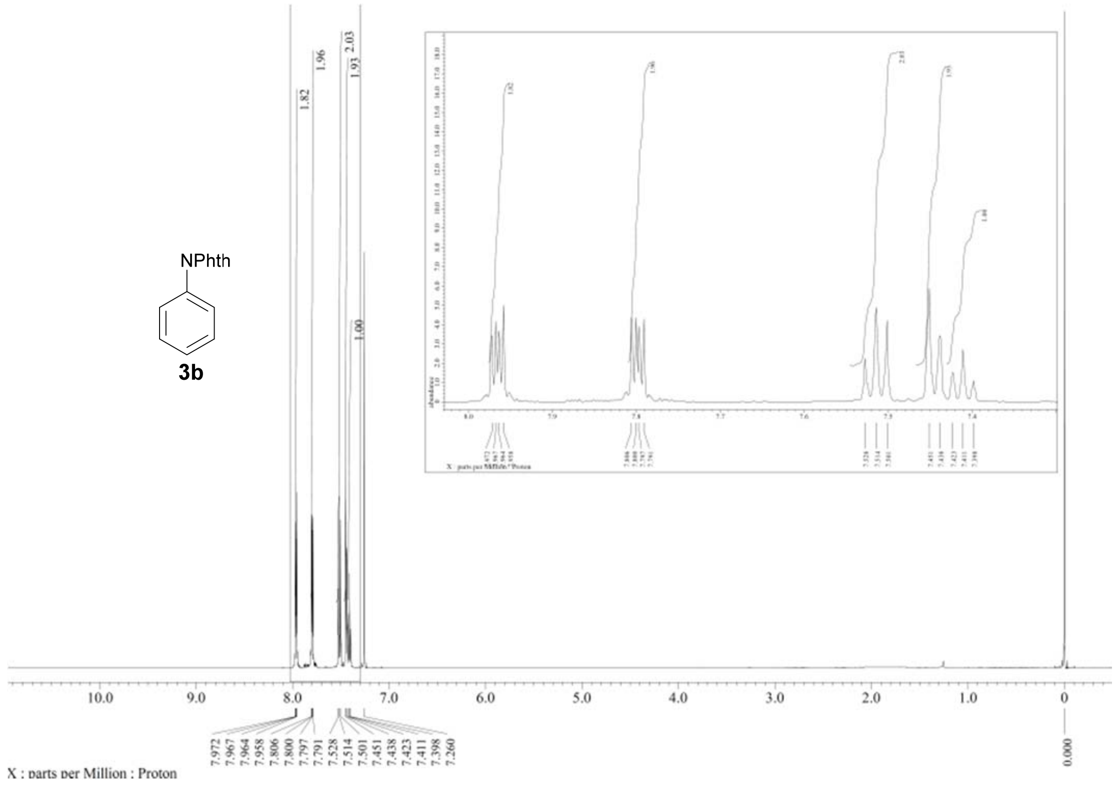
NPhth
3b
1.82
1.96
2.03
1.93
1.00
10.0
9.0
8.0
7.0
6.0
5.0
4.0
3.0
2.0
1.0
0
7.972
7.967
7.964
7.958
7.806
7.800
7.797
7.791
7.528
7.514
7.501
7.451
7.438
7.423
7.411
7.398
7.260
0.000
X : parts per Million : Proton

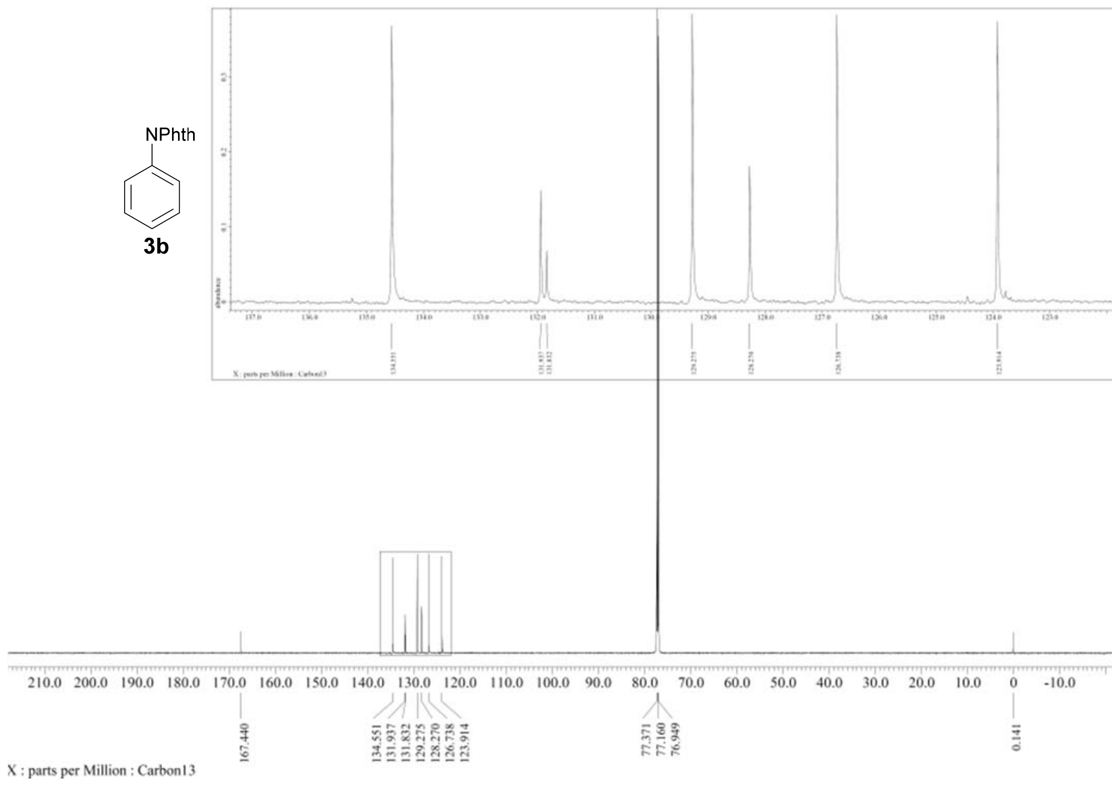
NPhth
3b
210.0 200.0 190.0 180.0 170.0 160.0 150.0 140.0 130.0 120.0 110.0 100.0 90.0 80.0 70.0 60.0 50.0 40.0 30.0 20.0 10.0 0 -10.0
167.440
134.551
131.937
131.832
129.275
128.270
126.738
123.914
77.371
77.160
76.949
0.141
X : parts per Million : Carbon13

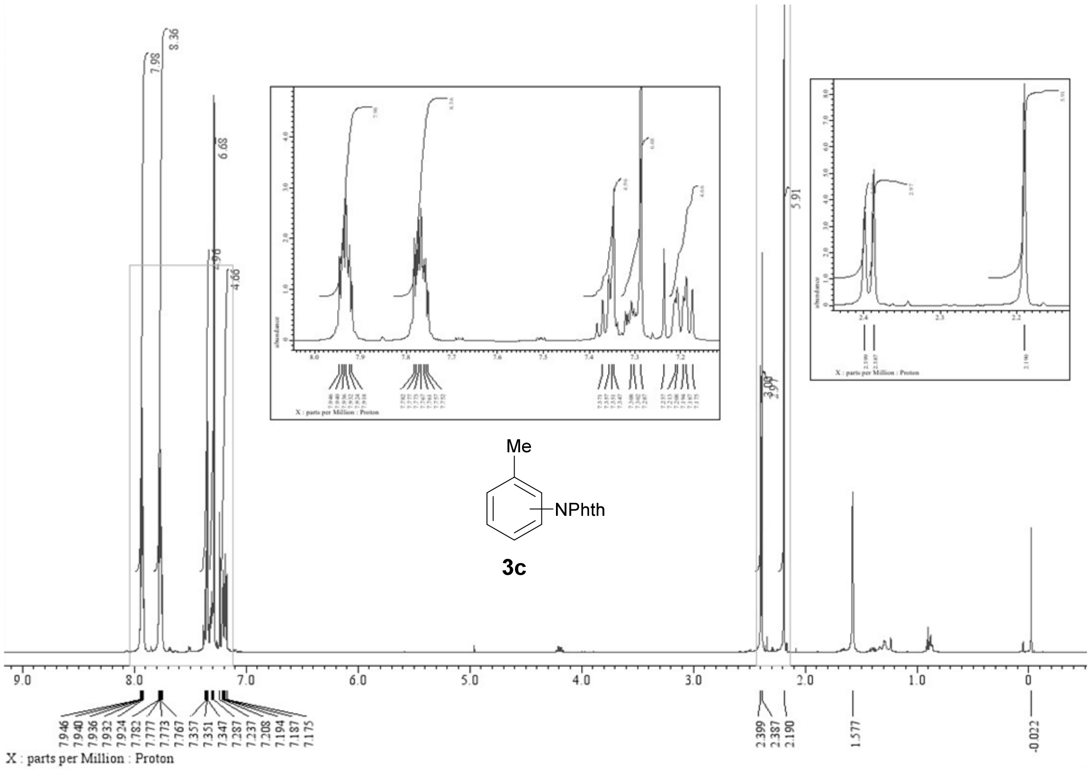
Me
NPhth
3c
7.946
7.940
7.936
7.932
7.924
7.782
7.777
7.773
7.767
7.357
7.351
7.347
7.287
7.237
7.208
7.194
7.187
7.175
2.399
2.387
2.190
1.577
-0.022
X : parts per Million : Proton

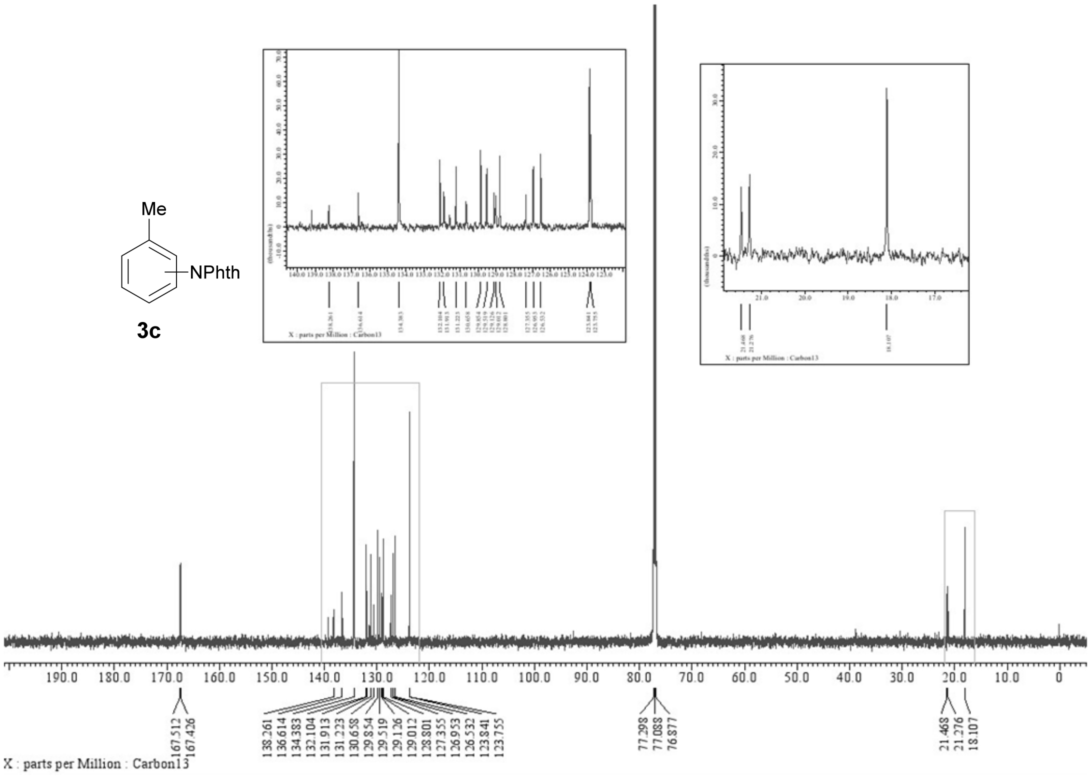
Me
NPhth
3c
167.512
167.426
138.261
136.614
134.383
132.104
131.913
131.223
130.658
129.854
129.519
129.126
129.012
128.801
127.355
126.953
126.532
123.841
123.755
77.298
77.083
76.877
21.468
21.276
18.107
X : parts per Million : Carbon13

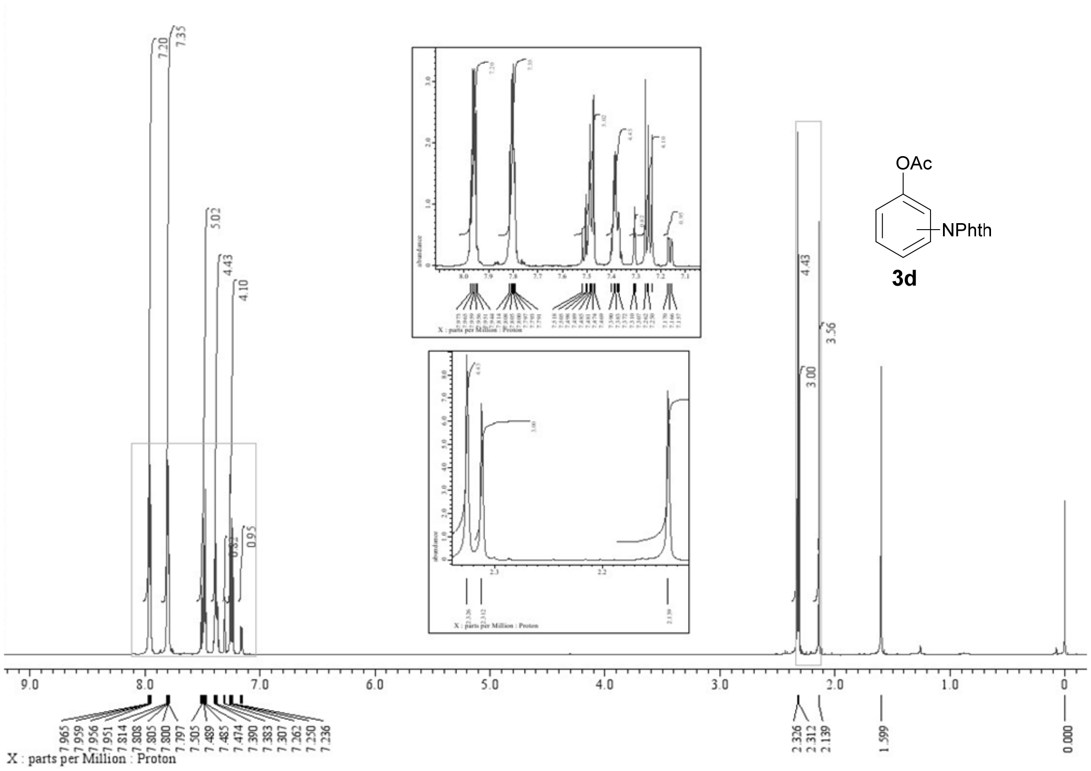
OAc
NPhth
3d
7.965
7.959
7.956
7.951
7.814
7.808
7.805
7.800
7.797
7.505
7.489
7.485
7.474
7.390
7.383
7.307
7.262
7.250
7.236
2.326
2.312
2.139
1.599
0.000
X : parts per Million : Proton

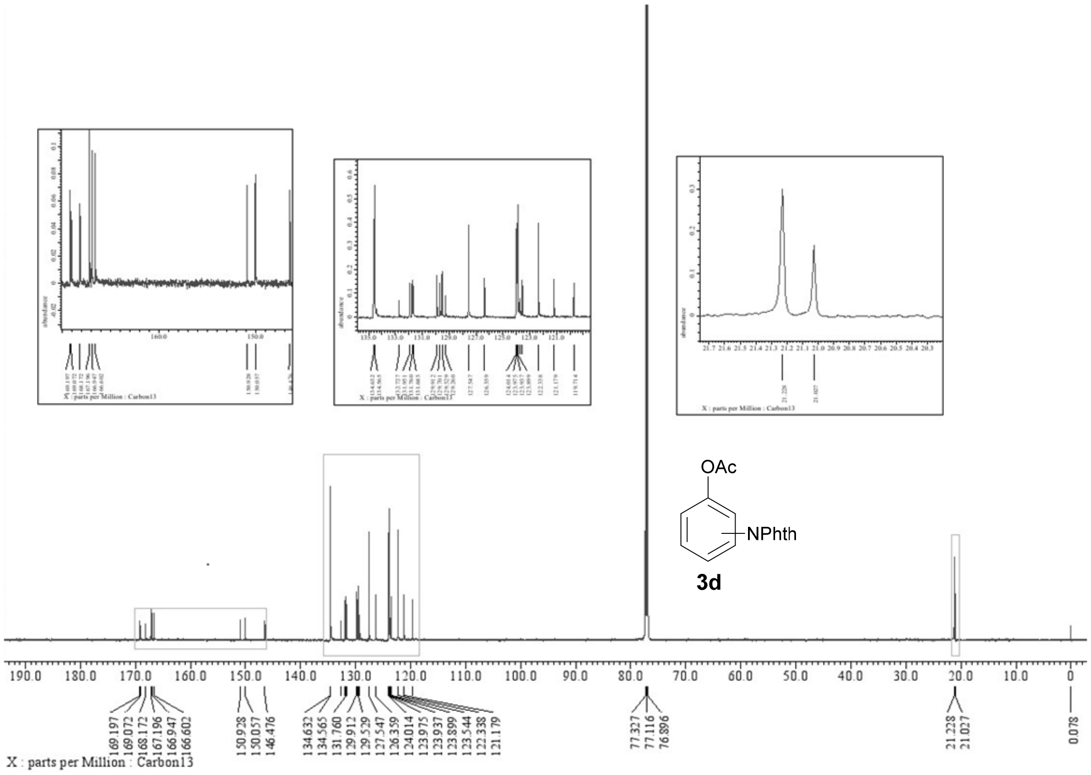
OAc
NPhth
3d
169.197
169.072
168.172
167.196
166.947
166.602
150.928
150.057
146.476
134.632
134.565
131.760
129.912
129.529
127.547
126.359
124.014
123.975
123.937
123.899
123.544
122.338
121.179
77.327
77.116
76.896
21.228
21.027
0.078
X : parts per Million : Carbon13

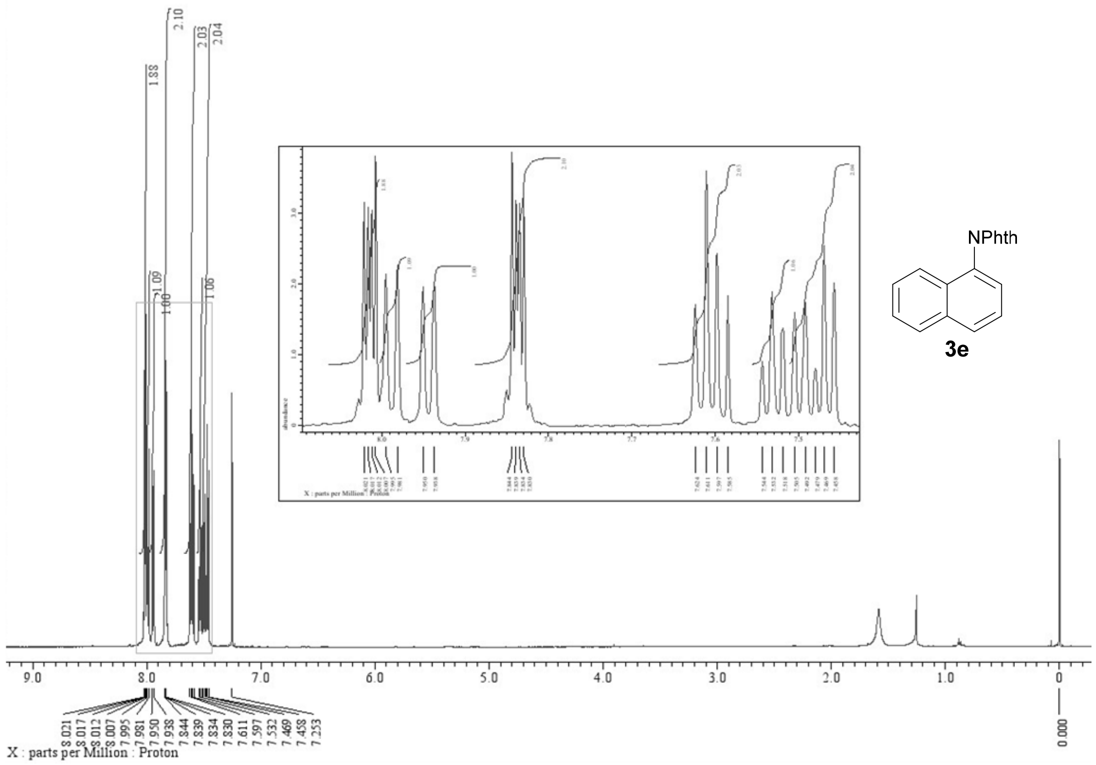

NPhth
3e
8.021
8.017
8.012
8.007
7.995
7.981
7.950
7.938
7.844
7.839
7.834
7.830
7.611
7.597
7.532
7.469
7.458
7.253
0.000
X : parts per Million : Proton


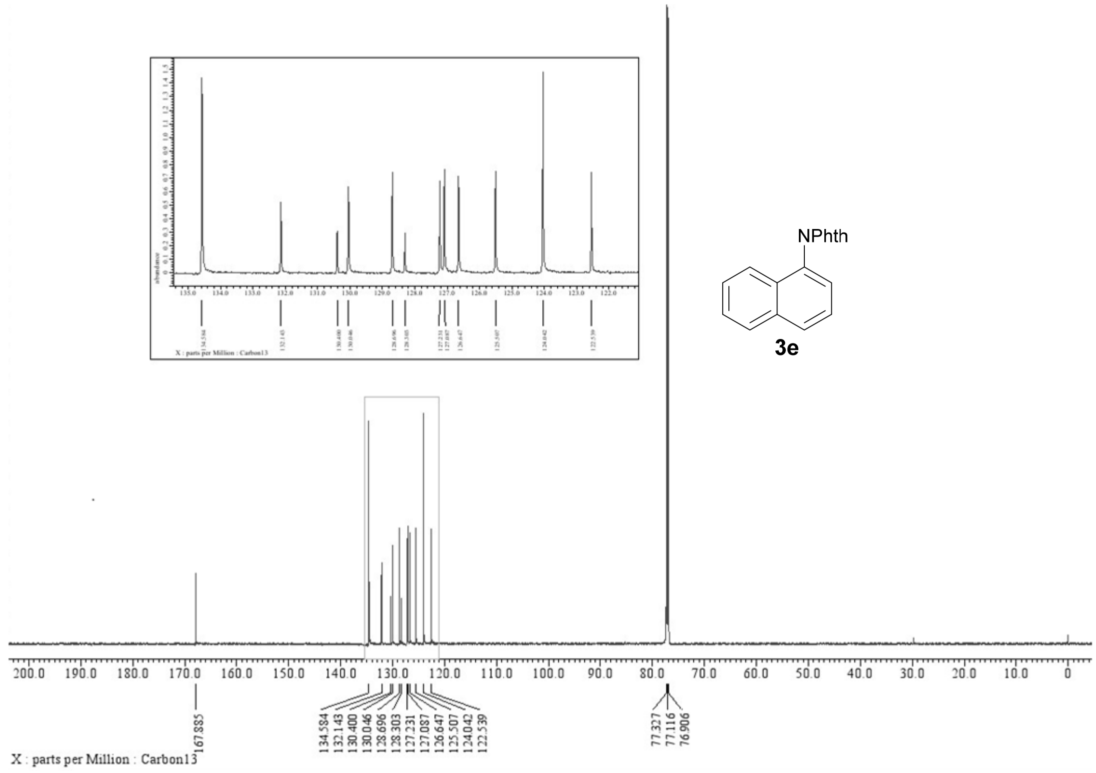

NPhth
3e
167.885
134.584
132.143
130.400
130.046
128.696
128.303
127.231
127.087
126.647
125.507
124.042
122.539
77.327
77.116
76.906
X : parts per Million : Carbon13

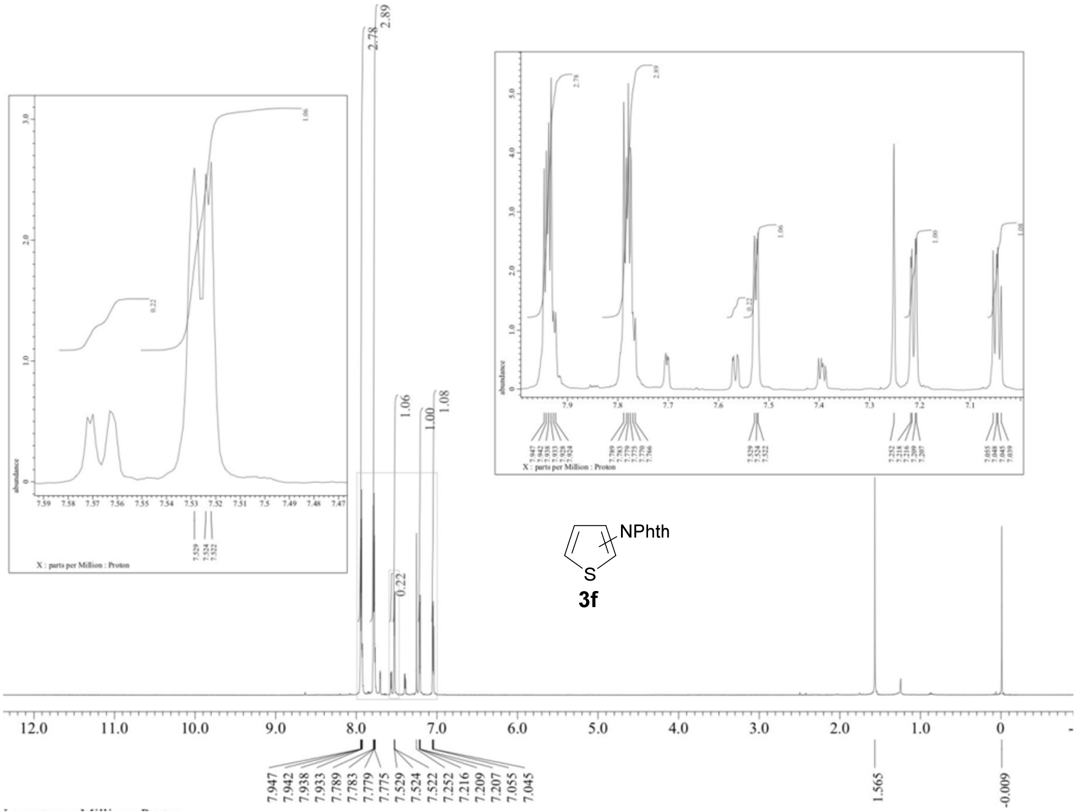
NPhth
S
3f
2.78
2.89
1.06
0.22
1.00
1.08
12.0 11.0 10.0 9.0 8.0 7.0 6.0 5.0 4.0 3.0 2.0 1.0 0
7.947 7.942 7.938 7.933 7.789 7.783 7.779 7.775 7.529 7.524 7.522 7.252 7.216 7.209 7.207 7.055 7.045
1.565
-0.009
X : parts per Million : Proton

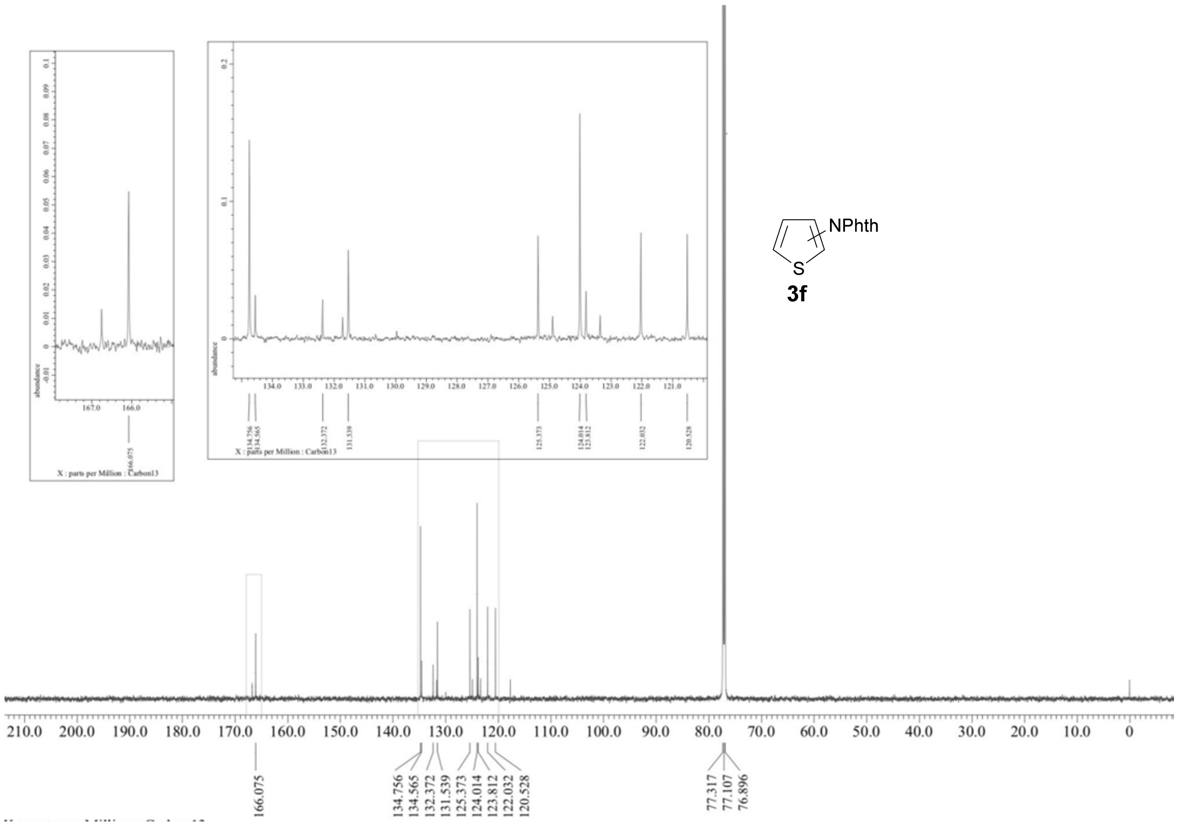
NPhth
S
3f
210.0 200.0 190.0 180.0 170.0 160.0 150.0 140.0 130.0 120.0 110.0 100.0 90.0 80.0 70.0 60.0 50.0 40.0 30.0 20.0 10.0 0
166.075
134.756 134.565 132.372 131.539 125.373 124.014 123.812 122.032 120.528
77.317 77.107 76.896
X : parts per Million : Carbon13

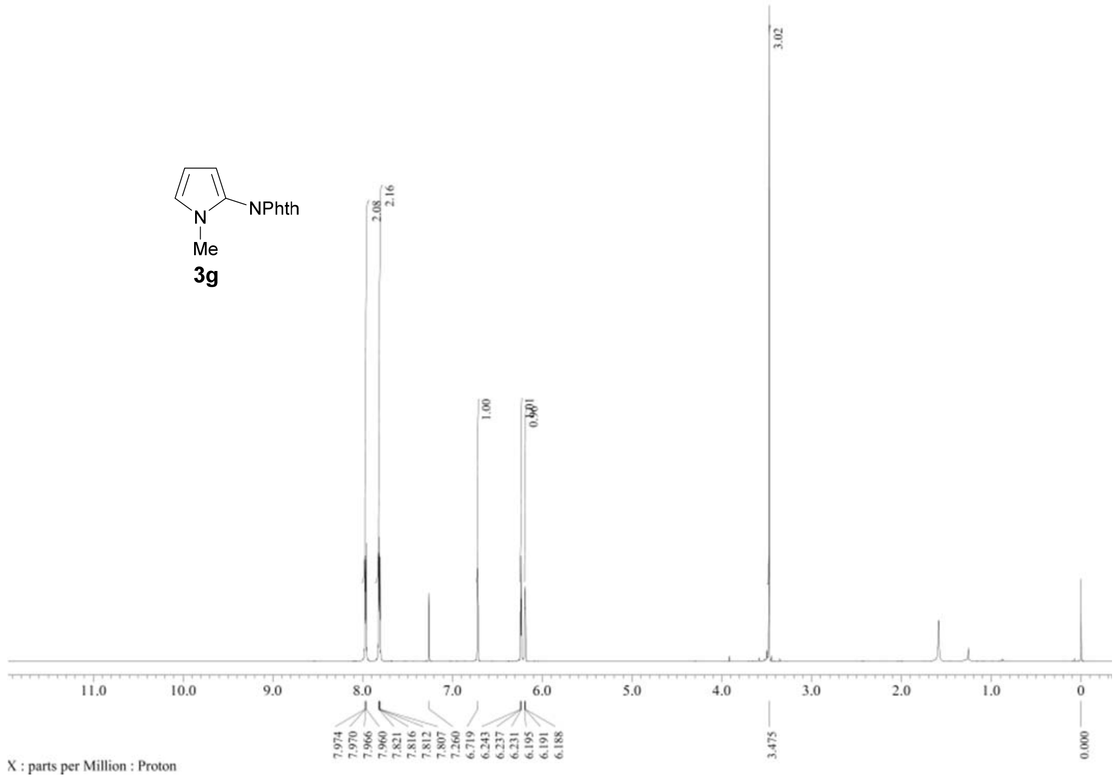
NPhth
N
Me
3g
2.08
2.16
1.00
1.01
0.96
3.02
11.0 10.0 9.0 8.0 7.0 6.0 5.0 4.0 3.0 2.0 1.0 0
7.974
7.970
7.966
7.960
7.821
7.816
7.812
7.807
7.260
6.719
6.243
6.237
6.231
6.195
6.191
6.188
3.475
0.000
X : parts per Million : Proton

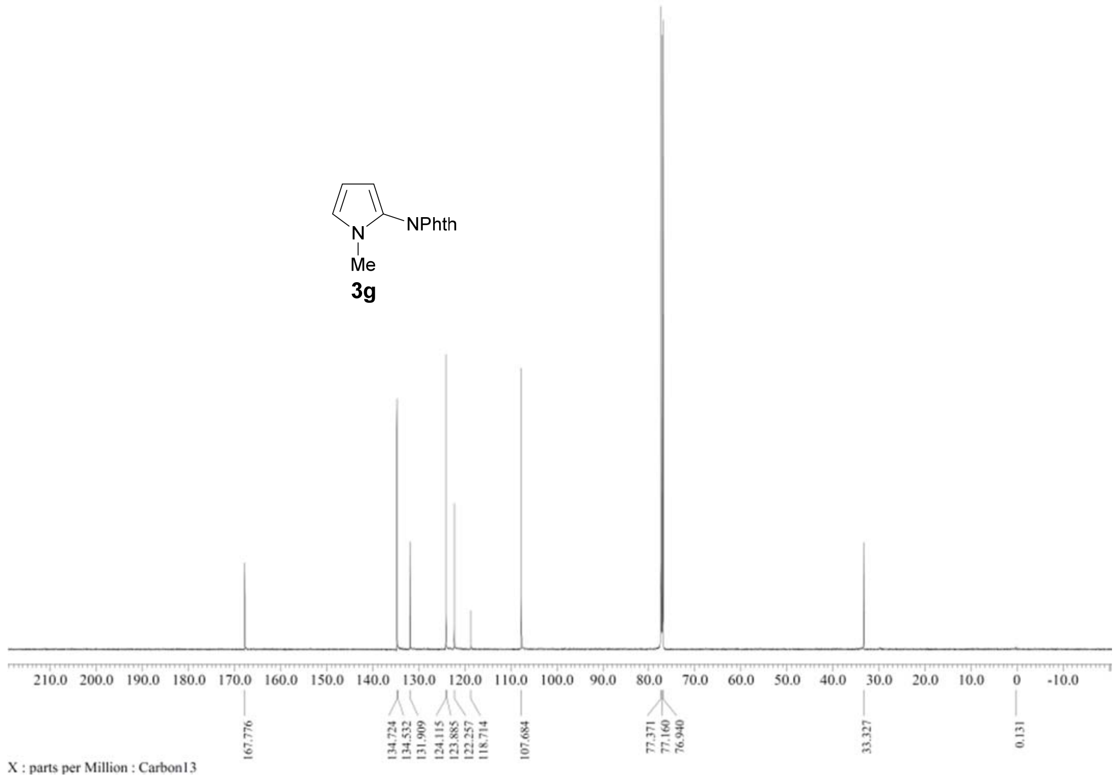
NPhth
N
Me
3g
210.0 200.0 190.0 180.0 170.0 160.0 150.0 140.0 130.0 120.0 110.0 100.0 90.0 80.0 70.0 60.0 50.0 40.0 30.0 20.0 10.0 0 -10.0
167.776
134.724
134.532
131.909
124.115
123.885
122.257
118.714
107.684
77.371
77.160
76.940
33.327
0.131
X : parts per Million : Carbon13

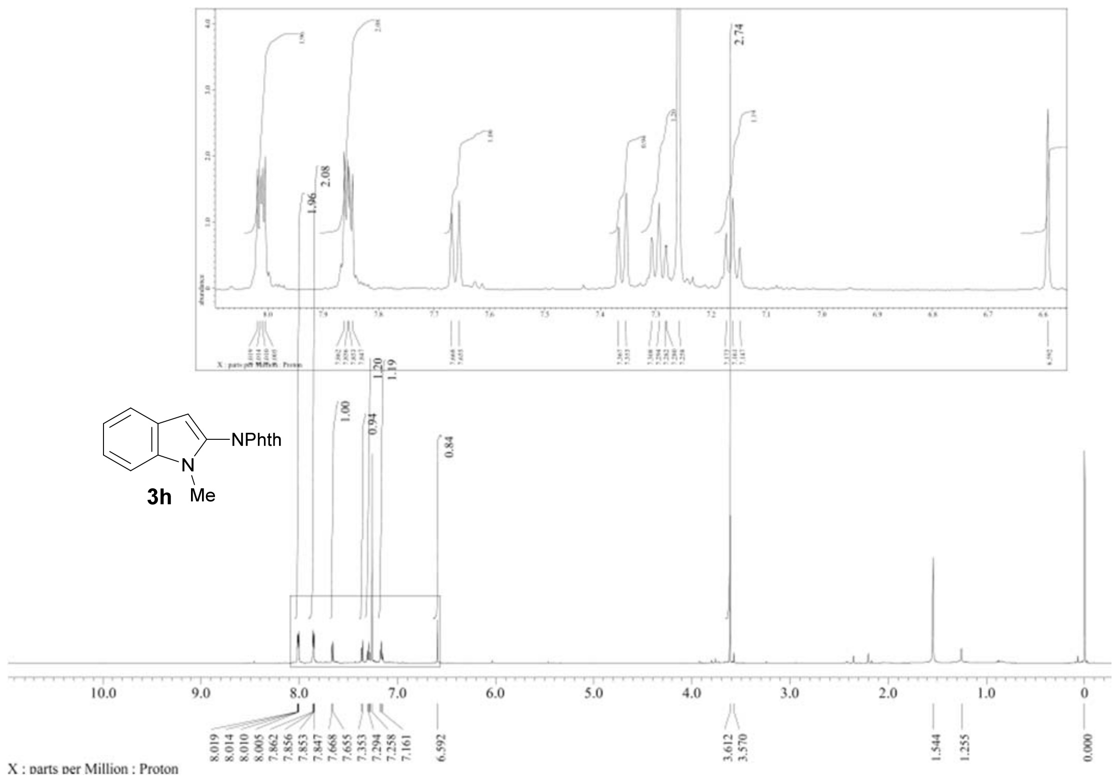
NPhth
N
3h
Me
X : parts per Million : Proton

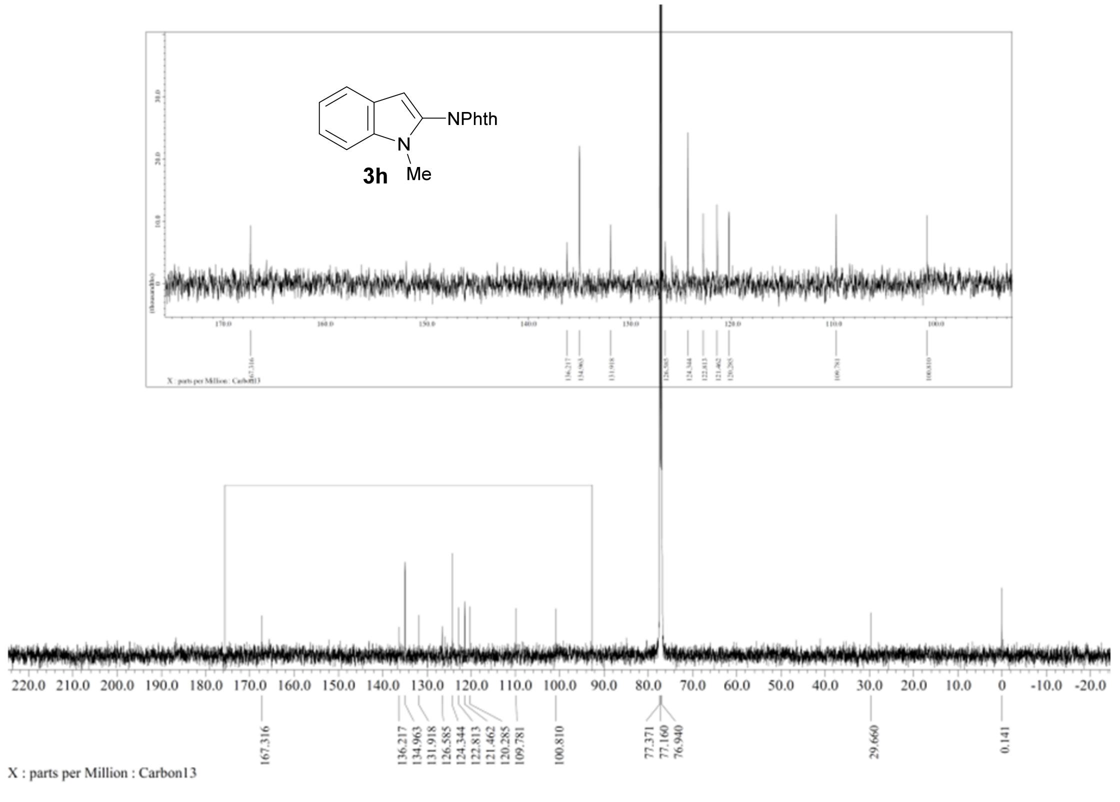
NPhth
N
3h
Me
X : parts per Million : Carbon13

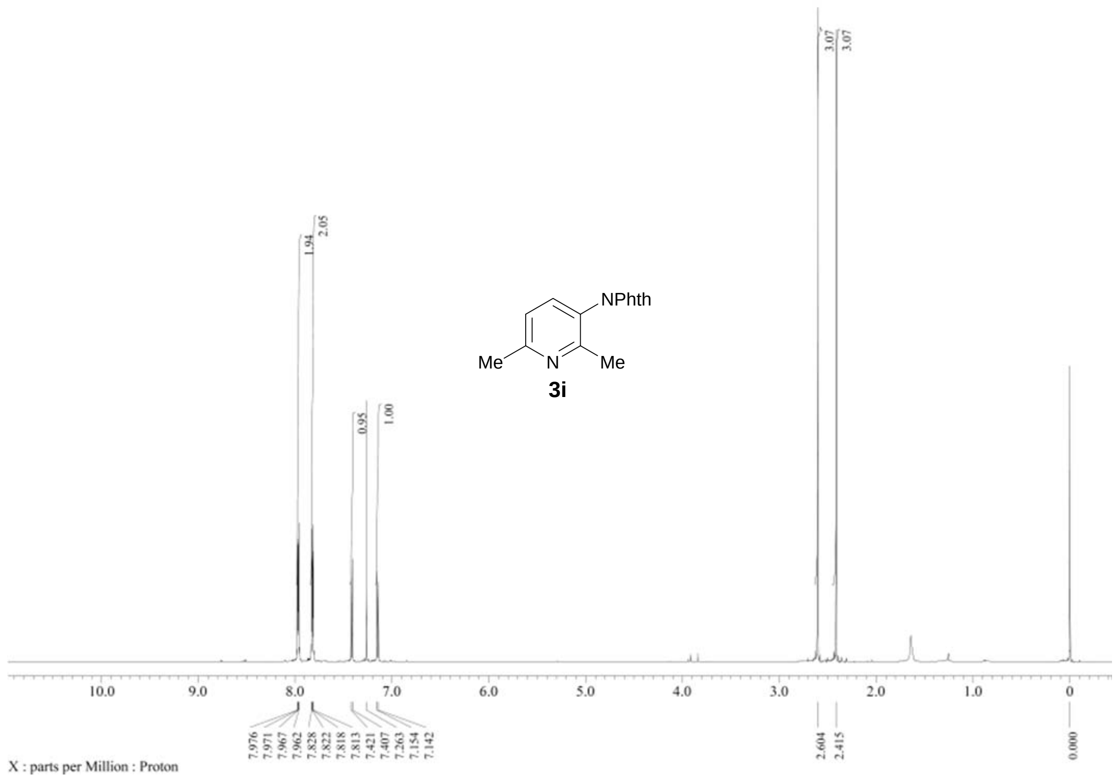
NPhth
Me
N
Me
3i
1.94
2.05
0.95
1.00
3.07
3.07
10.0
9.0
8.0
7.0
6.0
5.0
4.0
3.0
2.0
1.0
0
7.976
7.971
7.967
7.962
7.828
7.822
7.818
7.813
7.421
7.407
7.263
7.154
7.142
2.604
2.415
0.000
X : parts per Million : Proton

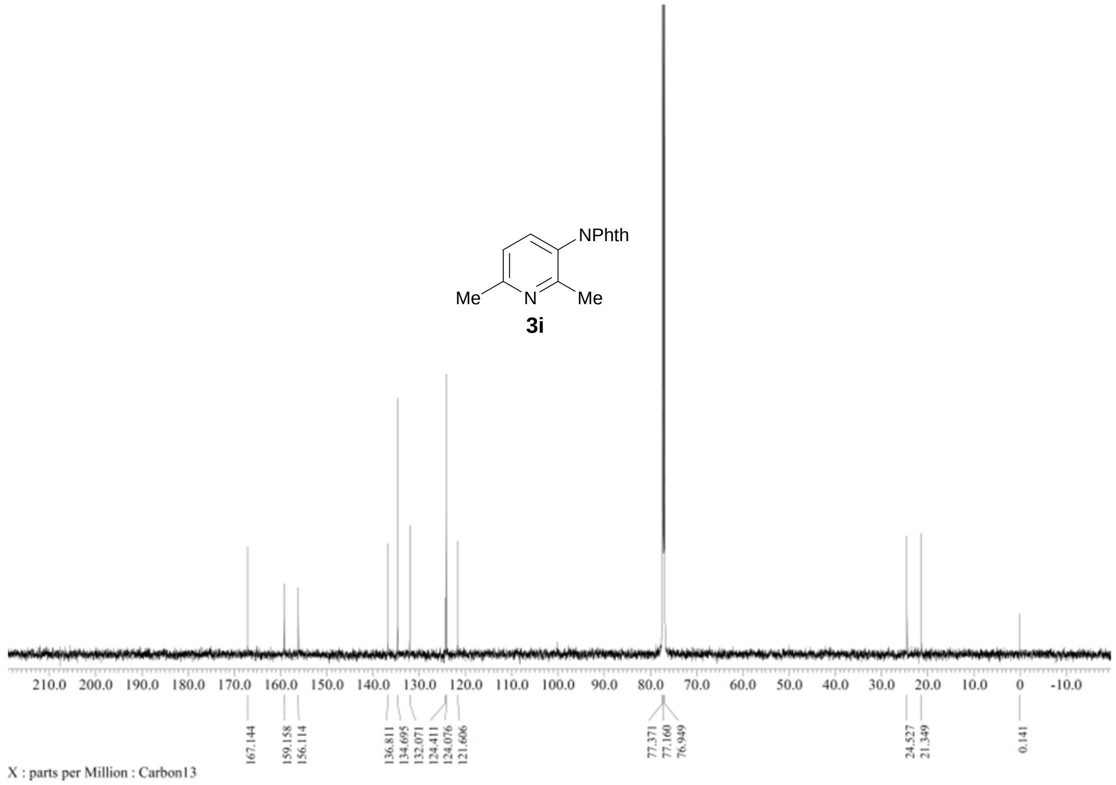
NPhth
Me
N
Me
3i
210.0
200.0
190.0
180.0
170.0
160.0
150.0
140.0
130.0
120.0
110.0
100.0
90.0
80.0
70.0
60.0
50.0
40.0
30.0
20.0
10.0
0
-10.0
167.144
159.158
156.114
136.811
134.695
132.071
124.411
124.076
121.606
77.371
77.160
76.949
24.527
21.349
0.141
X : parts per Million : Carbon13

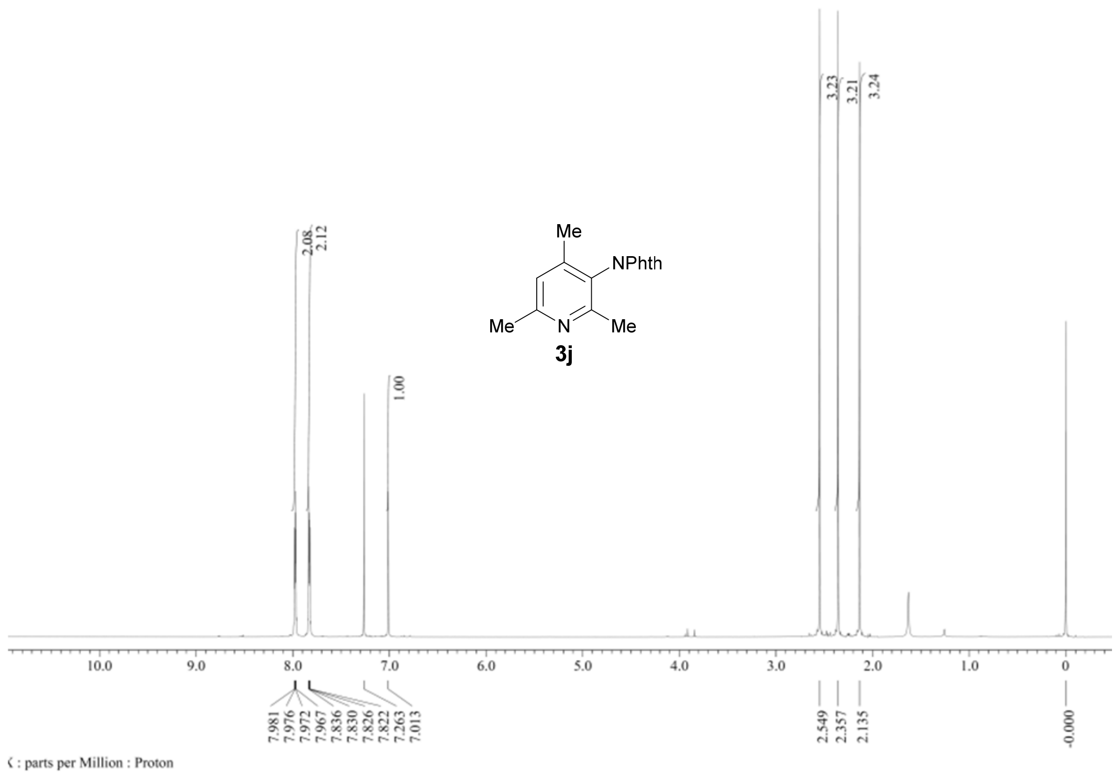
Me
NPhth
Me
N
Me
3j
2.08
2.12
1.00
3.23
3.21
3.24
10.0
9.0
8.0
7.0
6.0
5.0
4.0
3.0
2.0
1.0
0
7.981
7.976
7.972
7.967
7.836
7.830
7.826
7.822
7.263
7.013
2.549
2.357
2.135
-0.000
: parts per Million : Proton

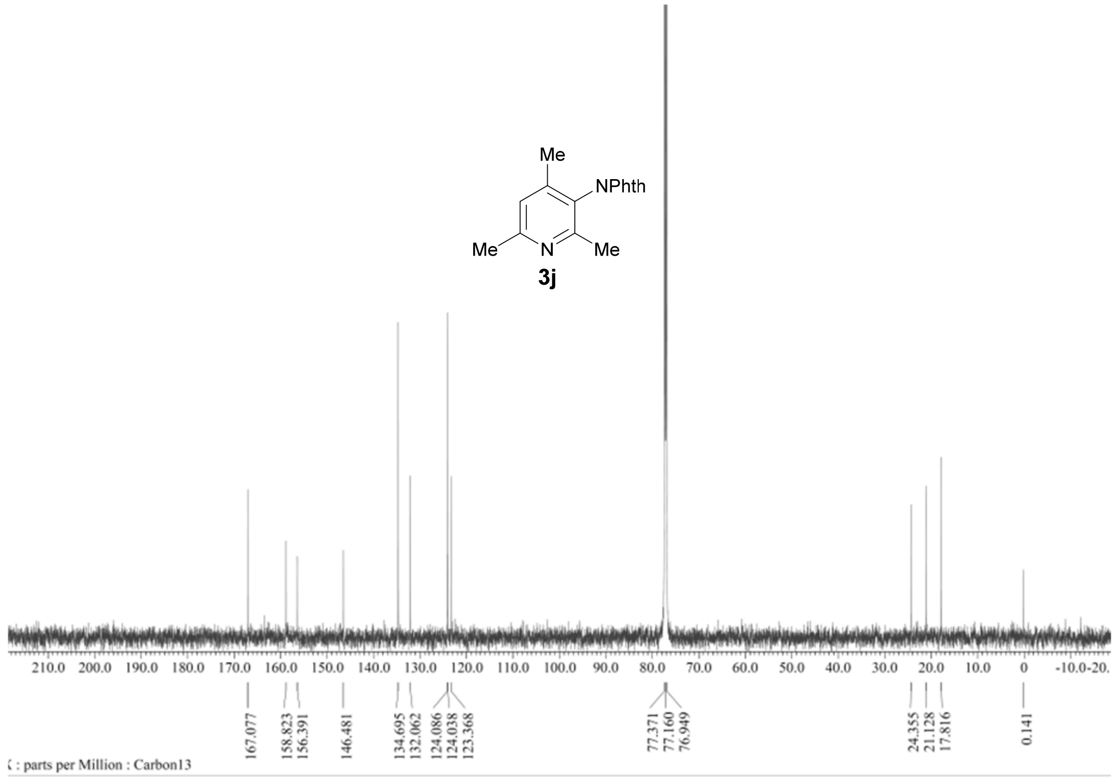
Me
NPhth
Me
N
Me
3j
210.0 200.0 190.0 180.0 170.0 160.0 150.0 140.0 130.0 120.0 110.0 100.0 90.0 80.0 70.0 60.0 50.0 40.0 30.0 20.0 10.0 0 -10.0-20.
167.077
158.823
156.391
146.481
134.695
132.062
124.086
124.038
123.368
77.371
77.160
76.949
24.355
21.128
17.816
0.141
: parts per Million : Carbon13

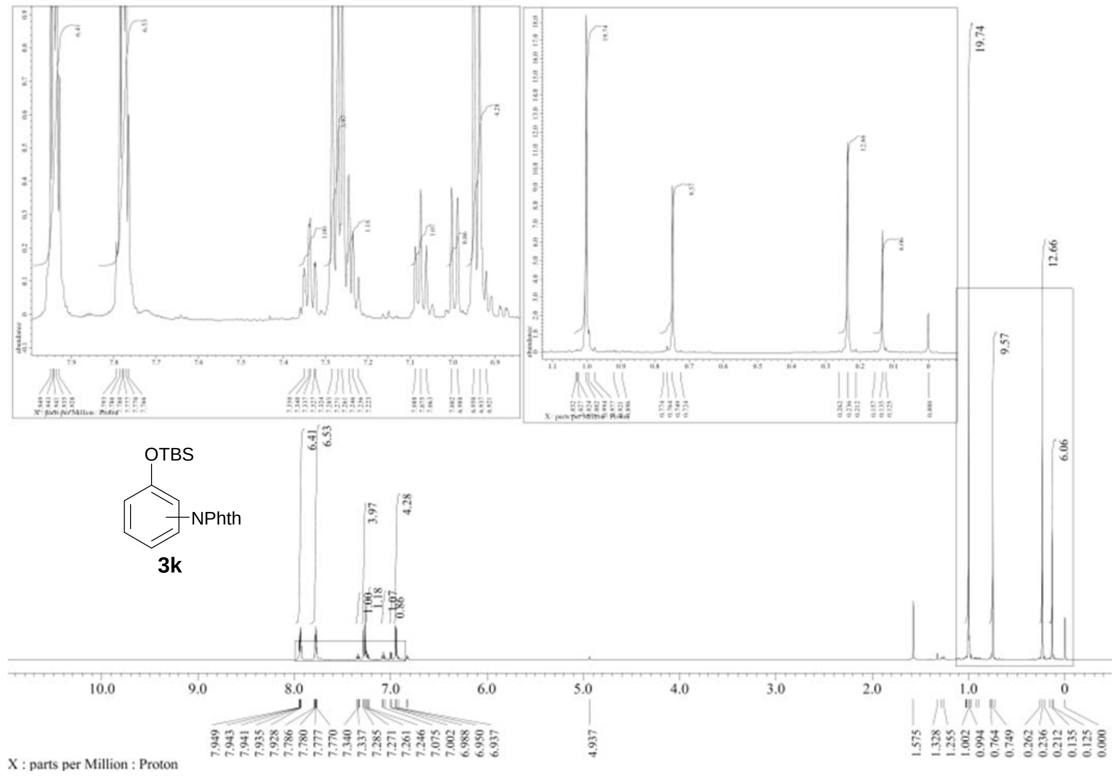
OTBS
NPhth
3k
X : parts per Million : Proton

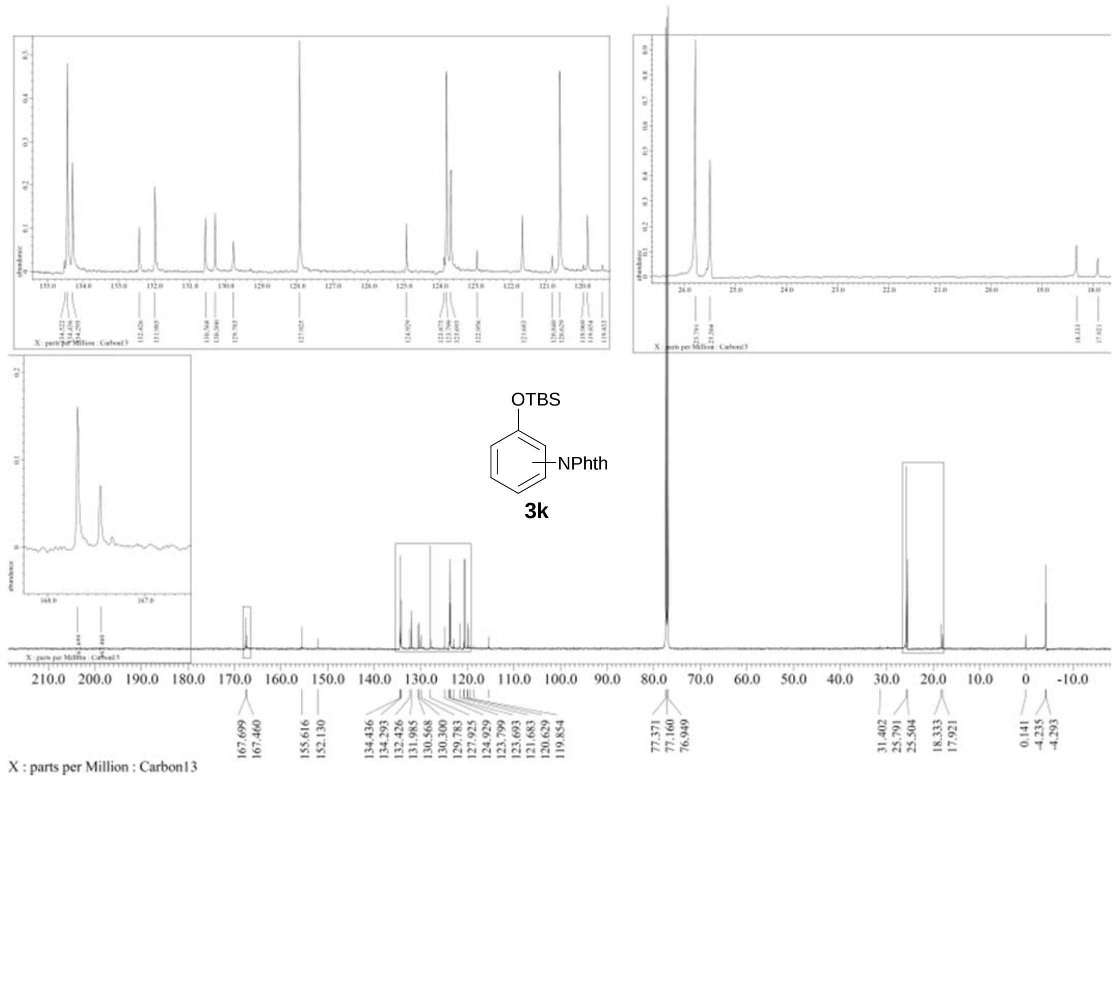
OTBS
NPhth
3k
X : parts per Million : Carbon13

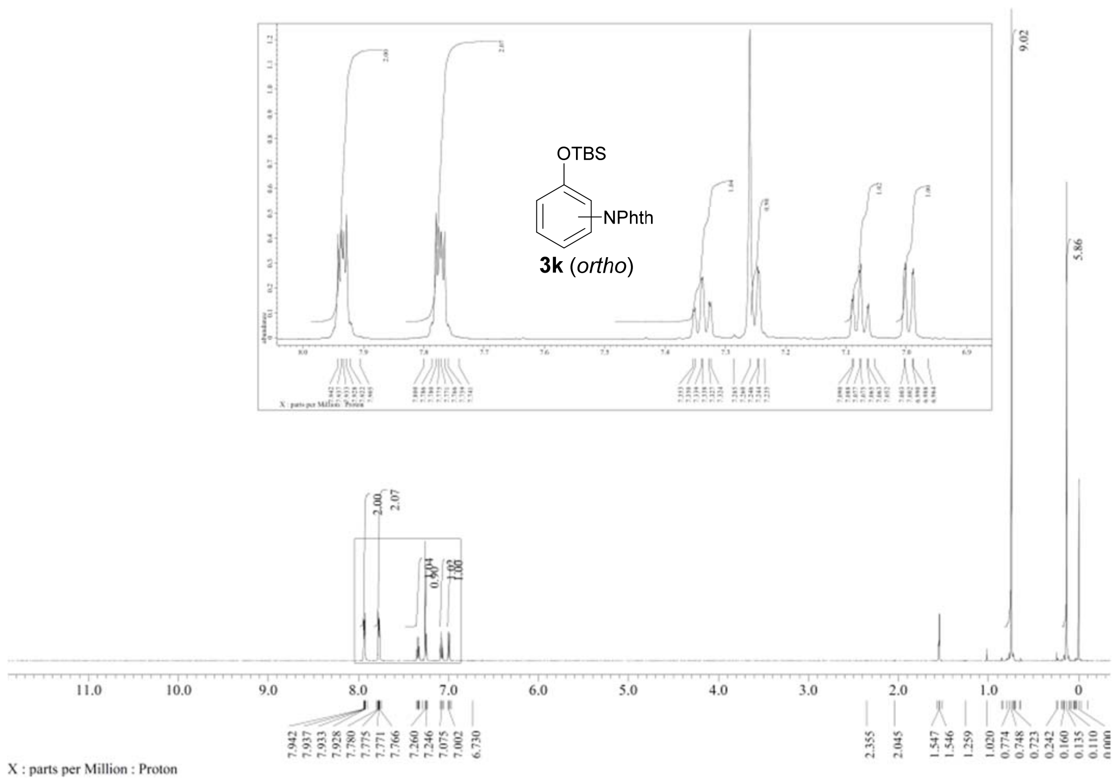
OTBS
NPhth
3k (ortho)
X : parts per Million : Proton

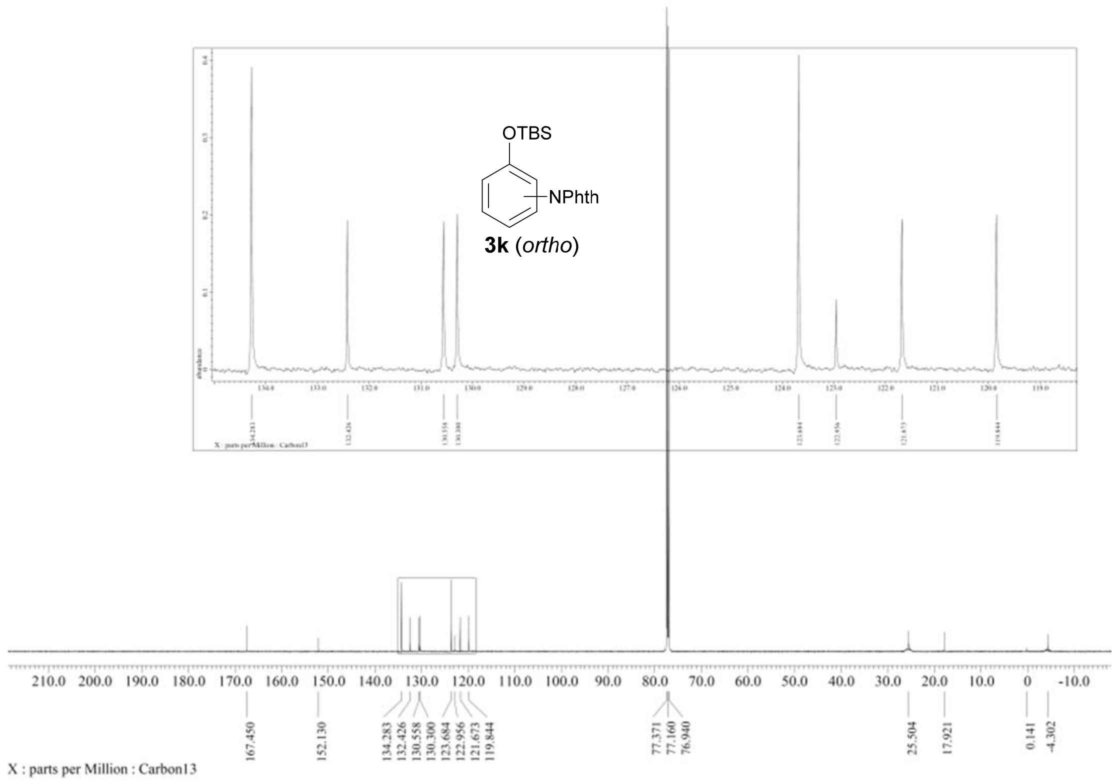
OTBS
NPhth
3k (ortho)
X : parts per Million : Carbon13

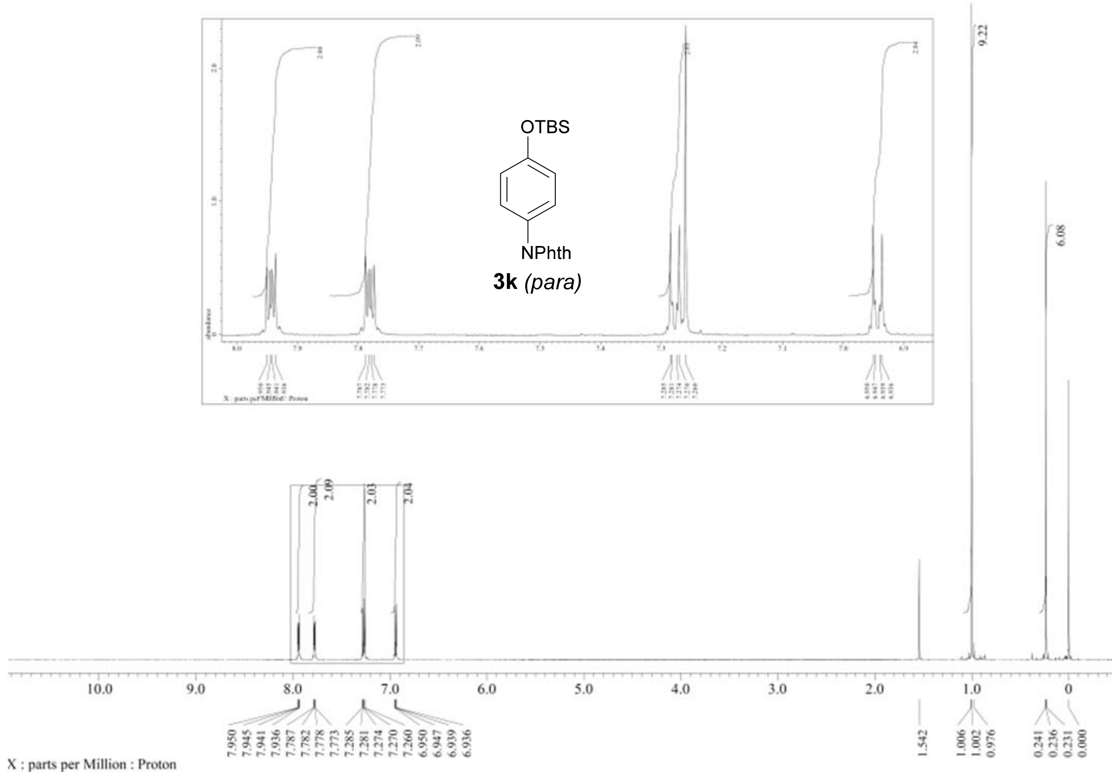
OTBS
NPhth
3k (para)
9.22
6.08
2.00
2.09
2.03
2.04
10.0
9.0
8.0
7.0
6.0
5.0
4.0
3.0
2.0
1.0
0
7.950
7.945
7.941
7.936
7.787
7.782
7.778
7.773
7.285
7.281
7.274
7.270
7.260
6.950
6.947
6.939
6.936
1.542
1.006
1.002
0.976
0.241
0.236
0.231
0.000
X : parts per Million : Proton

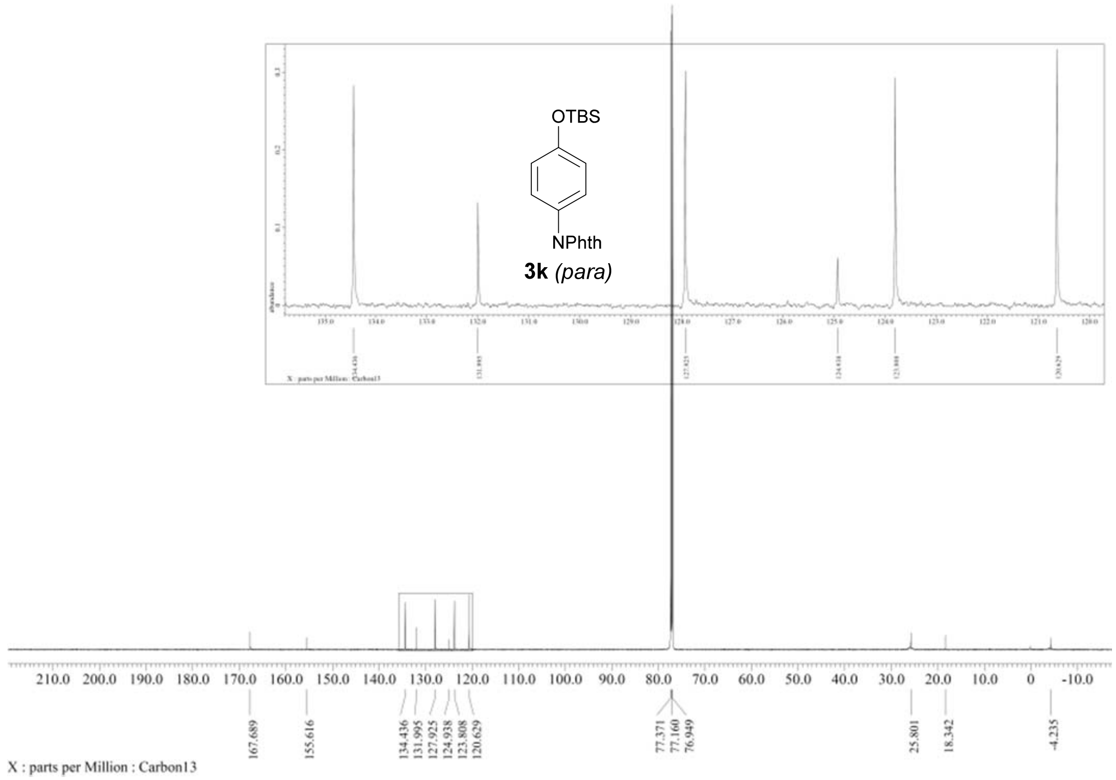
OTBS
NPhth
3k (para)
210.0
200.0
190.0
180.0
170.0
160.0
150.0
140.0
130.0
120.0
110.0
100.0
90.0
80.0
70.0
60.0
50.0
40.0
30.0
20.0
10.0
0
-10.0
167.689
155.616
134.436
131.995
127.925
124.938
123.808
120.629
77.371
77.160
76.949
25.801
18.342
-4.235
X : parts per Million : Carbon13

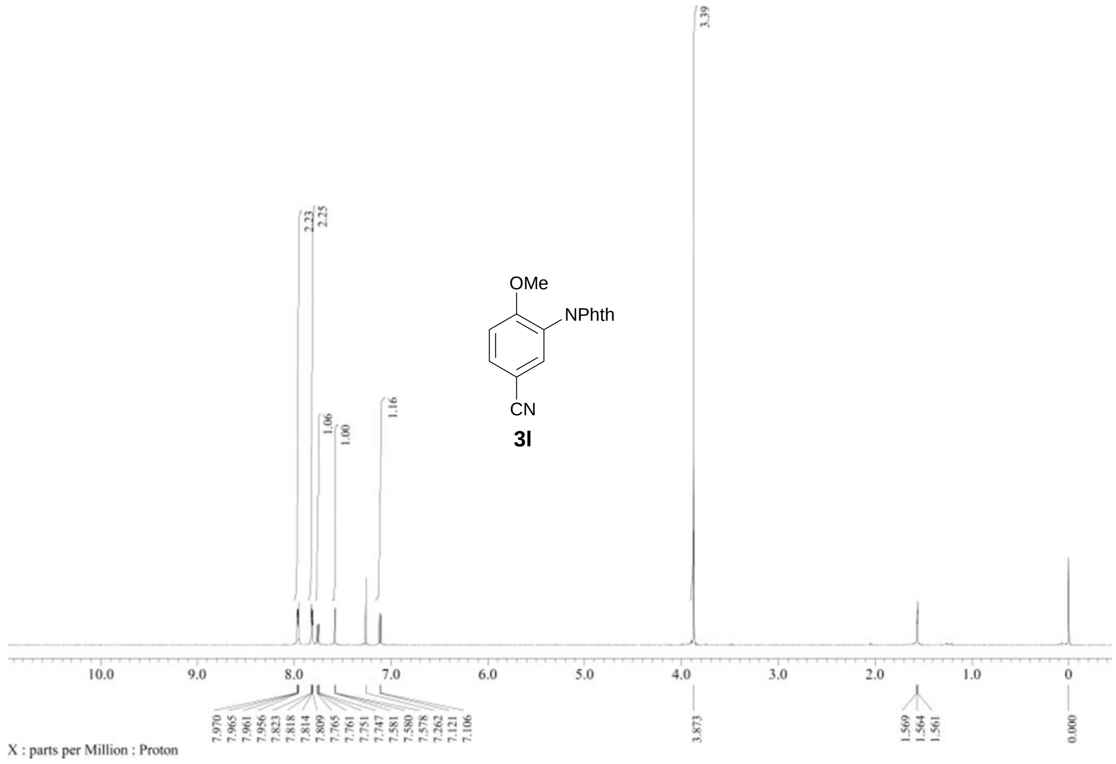

OMe
NPhth
CN
3l
2.23
2.25
1.06
1.00
1.16
3.39
10.0
9.0
8.0
7.0
6.0
5.0
4.0
3.0
2.0
1.0
0
7.970
7.965
7.961
7.956
7.823
7.818
7.814
7.809
7.765
7.761
7.751
7.747
7.581
7.580
7.578
7.262
7.121
7.106
3.873
1.569
1.564
1.561
0.000
X : parts per Million : Proton


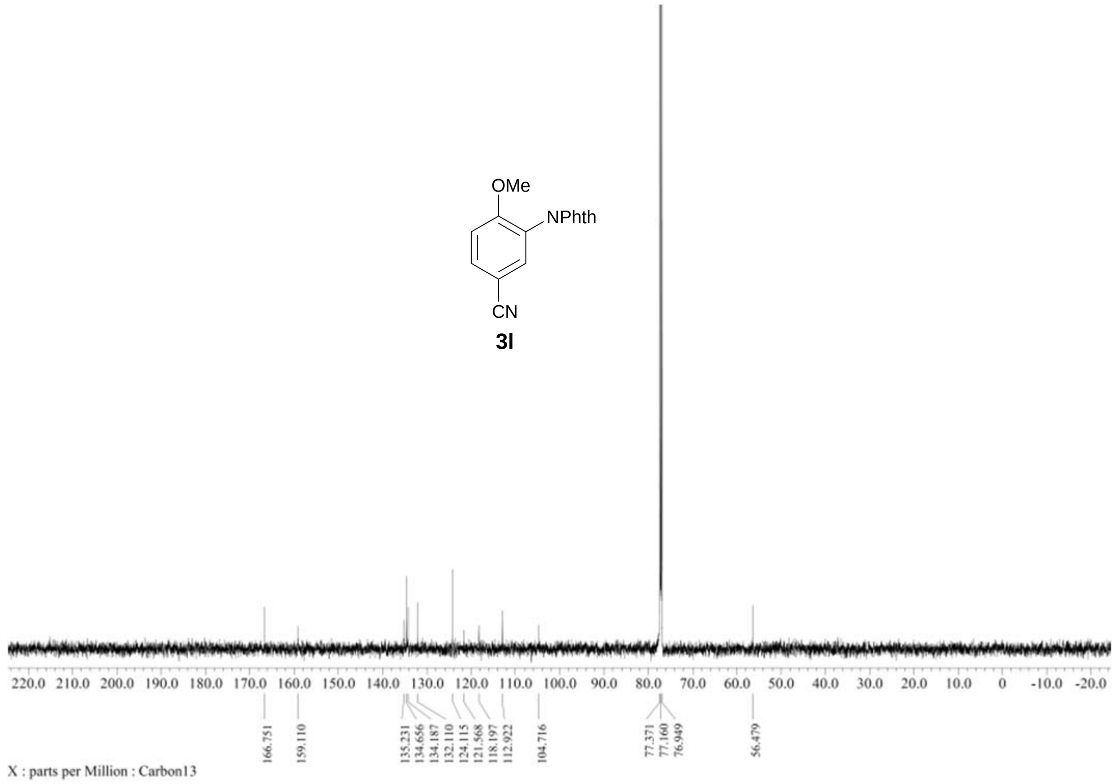

OMe
NPhth
CN
3l
220.0
210.0
200.0
190.0
180.0
170.0
160.0
150.0
140.0
130.0
120.0
110.0
100.0
90.0
80.0
70.0
60.0
50.0
40.0
30.0
20.0
10.0
0
-10.0
-20.0
166.751
159.110
135.231
134.656
134.187
132.110
124.115
121.568
118.197
112.922
104.716
77.371
77.160
76.949
56.479
X : parts per Million : Carbon13

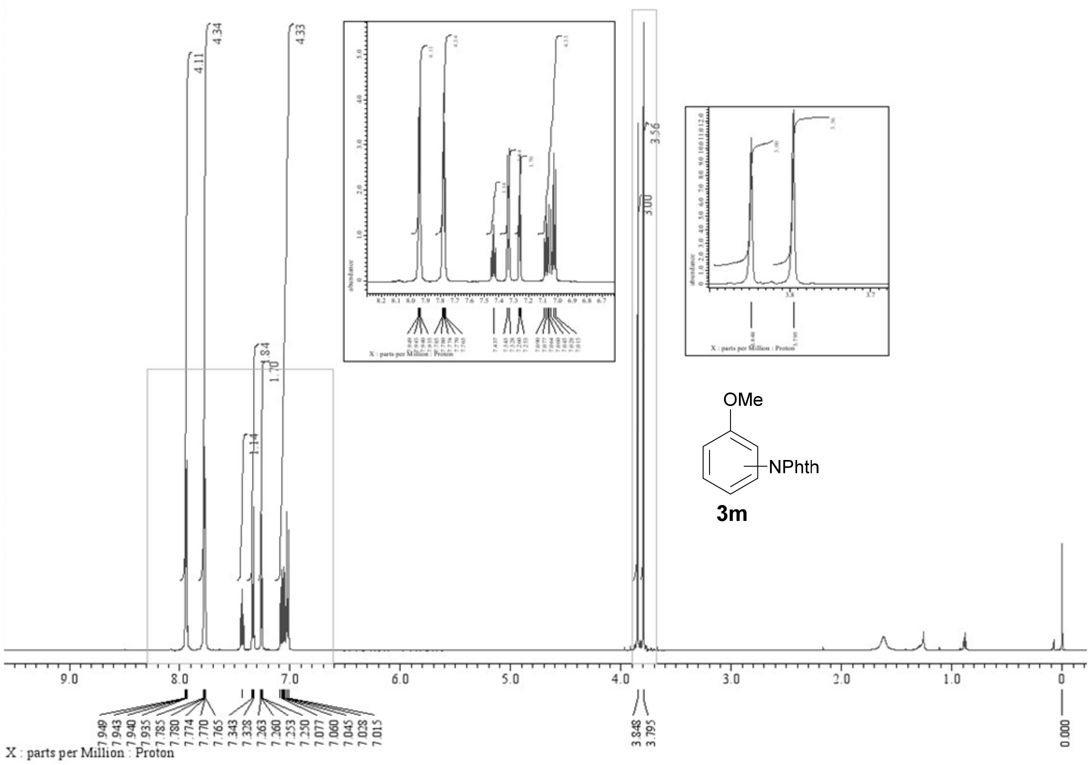
X : parts per Million : Proton
OMe
NPhth

3m

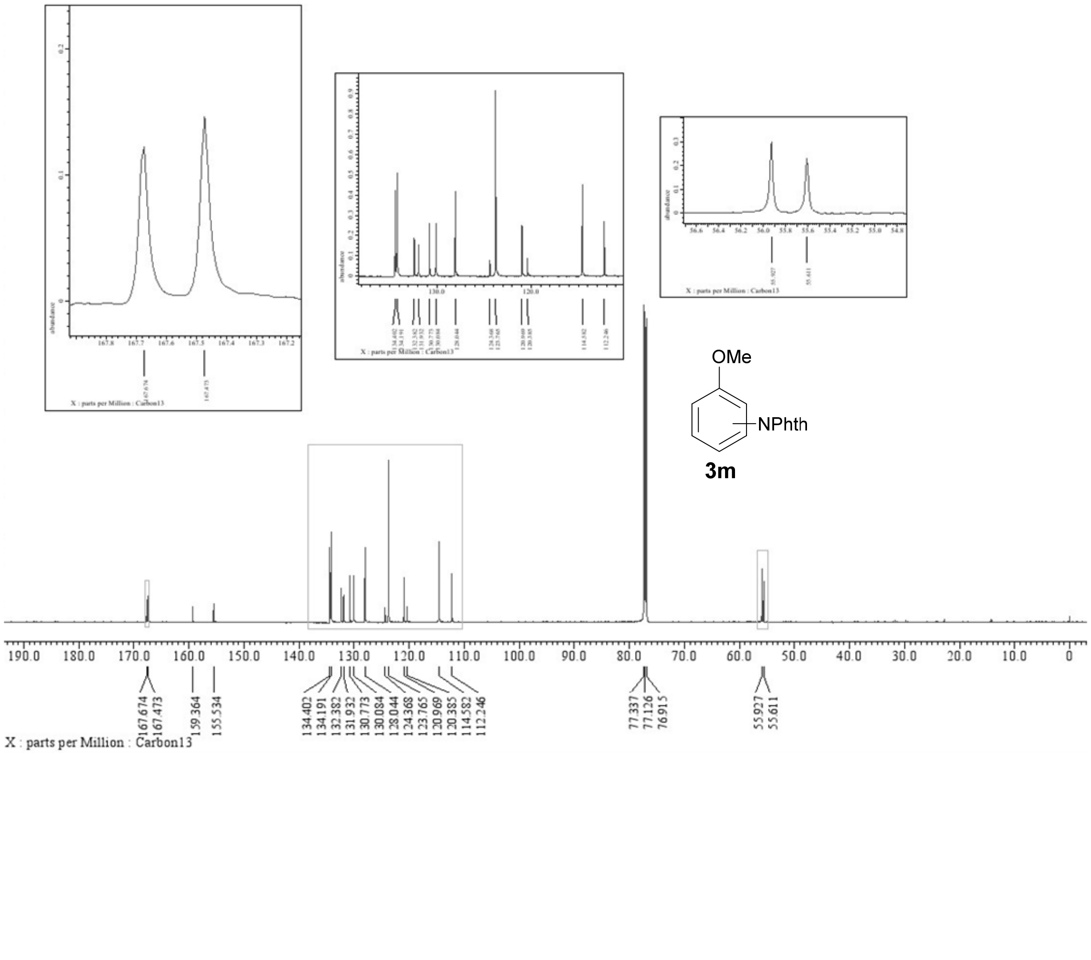
X : parts per Million : Carbon13
OMe
NPhth

3m

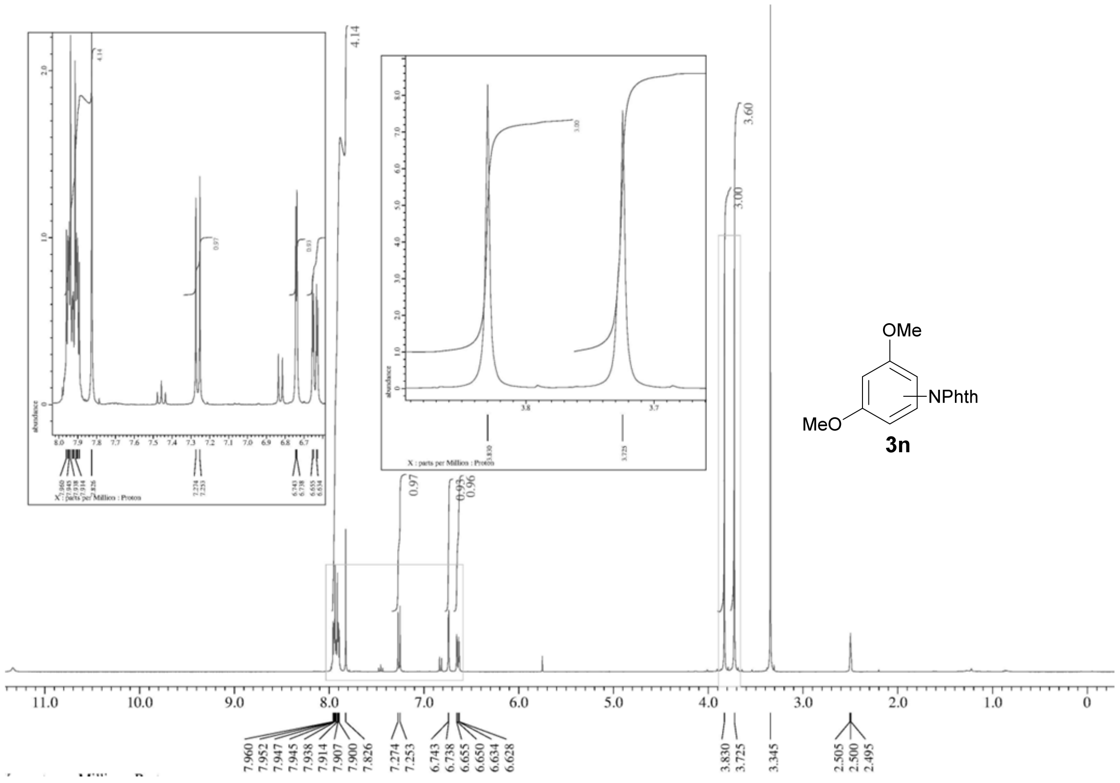
OMe
NPhth
MeO
3n
7.960 7.952 7.947 7.945 7.938 7.914 7.907 7.900 7.826 7.274 7.253 6.743 6.738 6.655 6.650 6.634 6.628
3.830 3.725 3.345 2.505 2.500 2.495
4.14 0.97 0.93 0.96 3.00 3.60
X : parts per Million : Proton

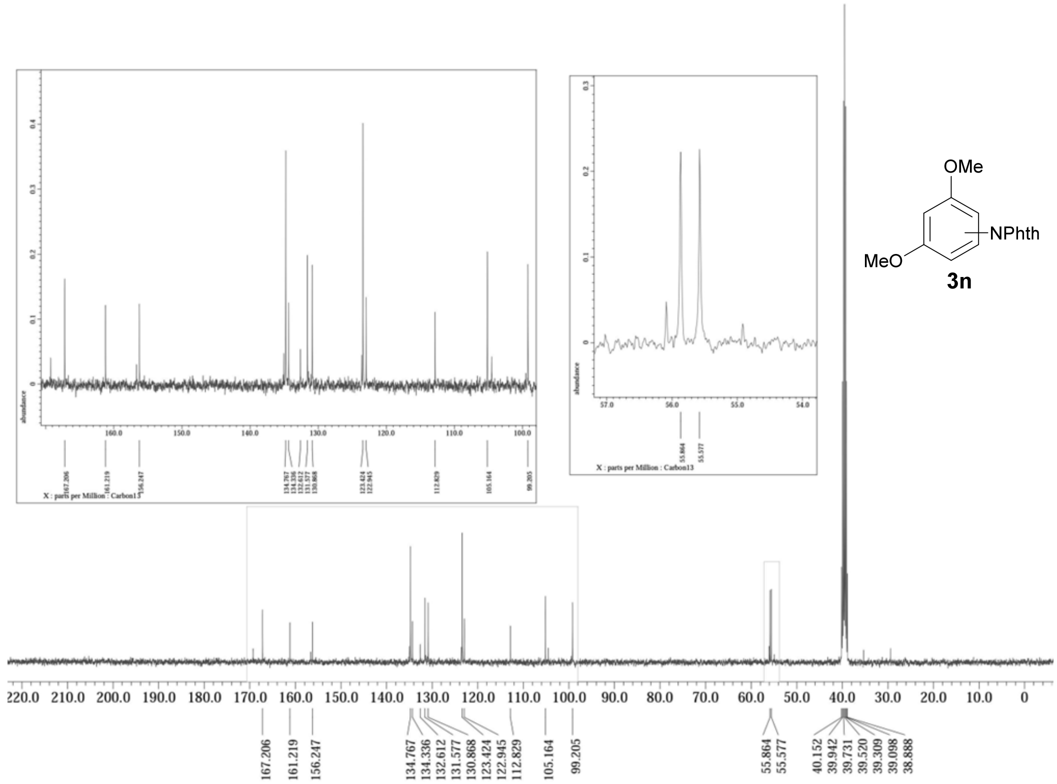
OMe
NPhth
MeO
3n
167.206 161.219 156.247 134.767 134.336 132.612 131.577 130.868 123.424 122.945 112.829 105.164 99.205
55.864 55.577 40.152 39.942 39.731 39.520 39.309 39.098 38.888
X : parts per Million : Carbon13

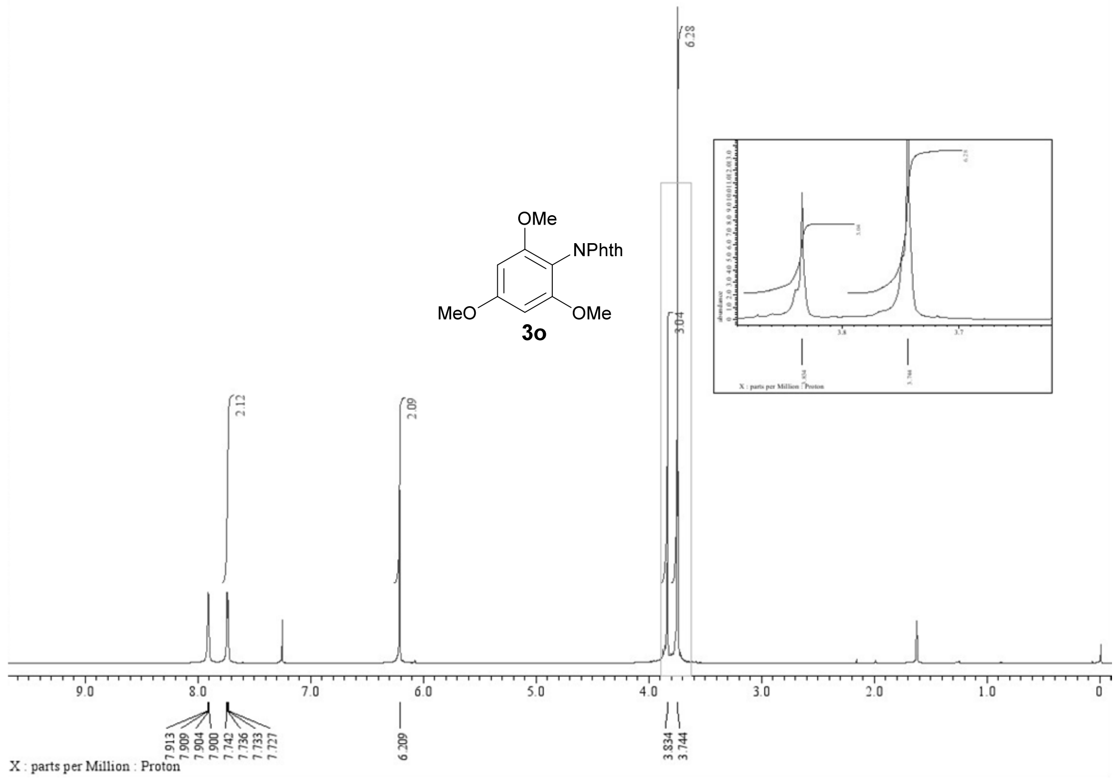
OMe
NPhth
MeO
OMe
3o
2.12
2.09
3.04
6.28
9.0
8.0
7.0
6.0
5.0
4.0
3.0
2.0
1.0
0
7.913
7.909
7.904
7.900
7.742
7.736
7.733
7.727
6.209
3.834
3.744
X : parts per Million : Proton

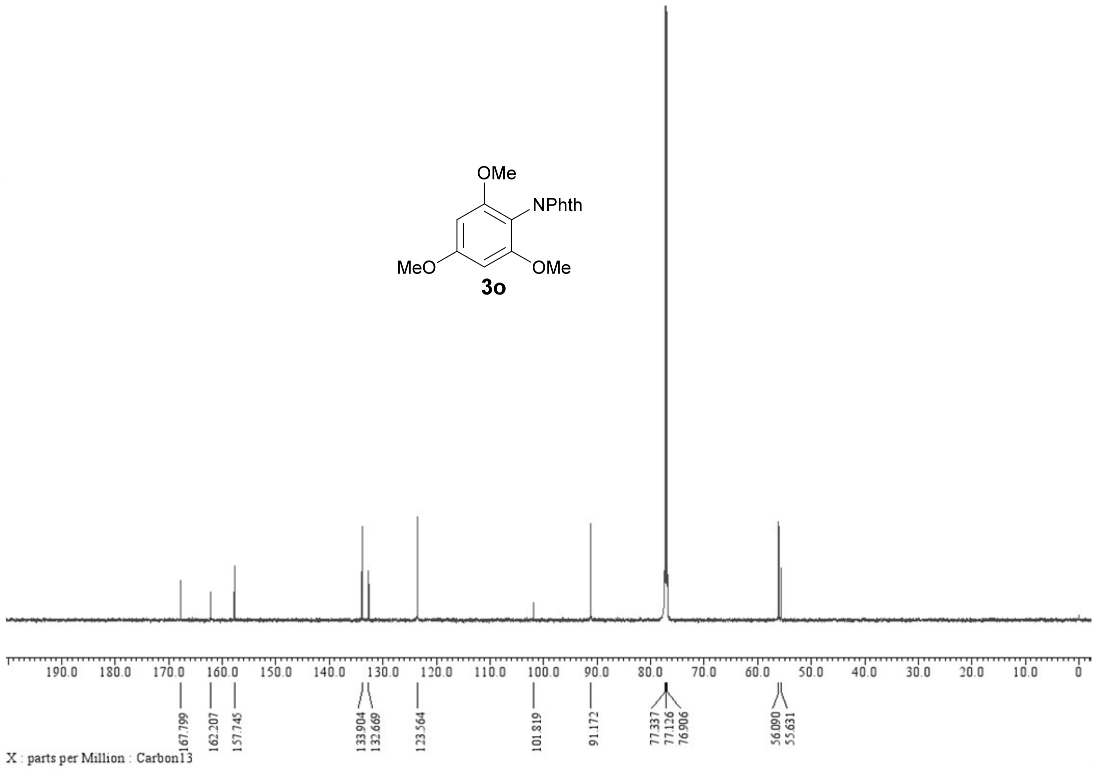
OMe
NPhth
MeO
OMe
3o
190.0
180.0
170.0
160.0
150.0
140.0
130.0
120.0
110.0
100.0
90.0
80.0
70.0
60.0
50.0
40.0
30.0
20.0
10.0
0
167.799
162.207
157.745
133.904
132.669
123.564
101.819
91.172
77.337
77.126
76.906
56.090
55.631
X : parts per Million : Carbon13

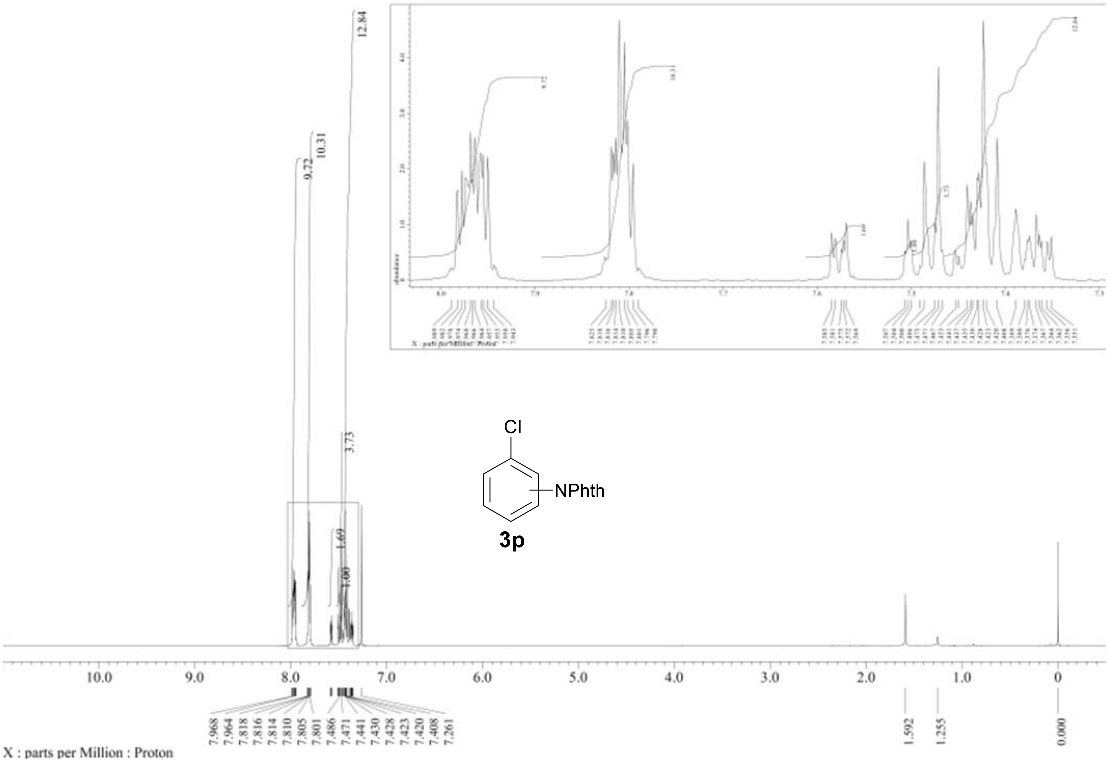
12.84
9.72
10.31
3.73
1.69
1.00
Cl
NPhth
3p
10.0 9.0 8.0 7.0 6.0 5.0 4.0 3.0 2.0 1.0 0
7.968 7.964 7.818 7.816 7.814 7.810 7.805 7.801 7.486 7.471 7.441 7.430 7.428 7.423 7.420 7.408 7.261
1.592
1.255
0.000
X : parts per Million : Proton

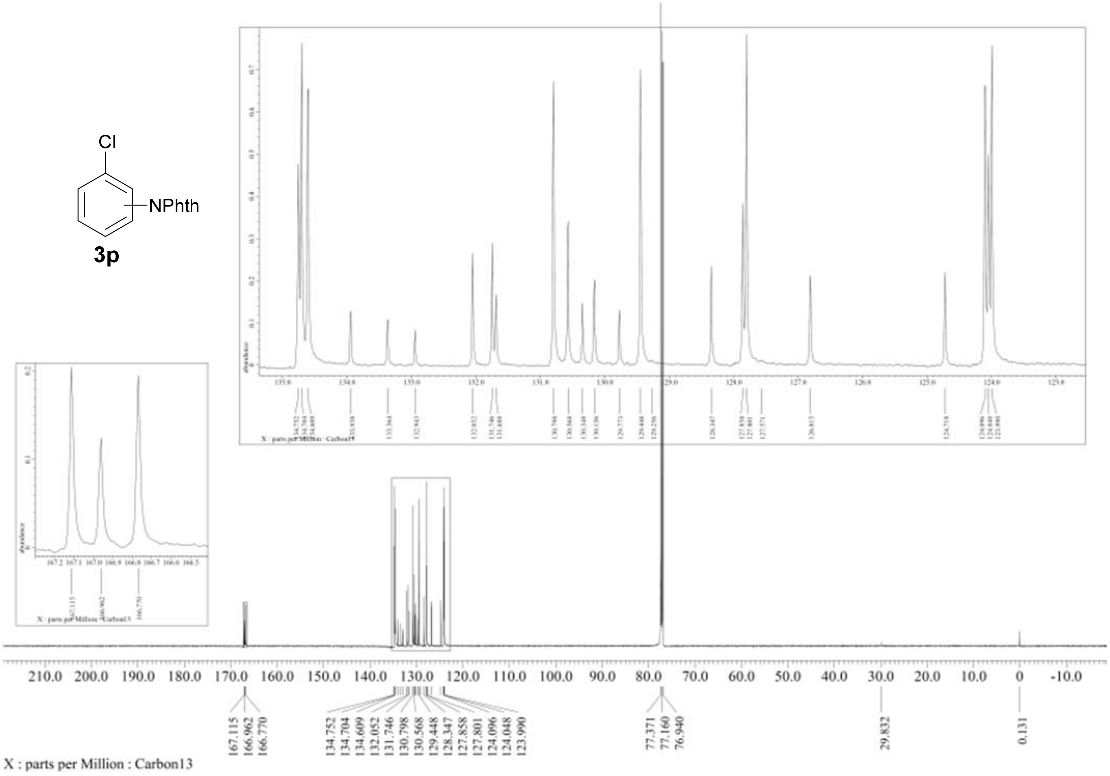
Cl
NPhth
3p
210.0 200.0 190.0 180.0 170.0 160.0 150.0 140.0 130.0 120.0 110.0 100.0 90.0 80.0 70.0 60.0 50.0 40.0 30.0 20.0 10.0 0 -10.0
167.115 166.962 166.770
134.752 134.704 134.609 132.052 131.746 130.798 130.568 129.448 128.347 127.858 127.801 124.096 124.048 123.990
77.371 77.160 76.940
29.832
0.131
X : parts per Million : Carbon13

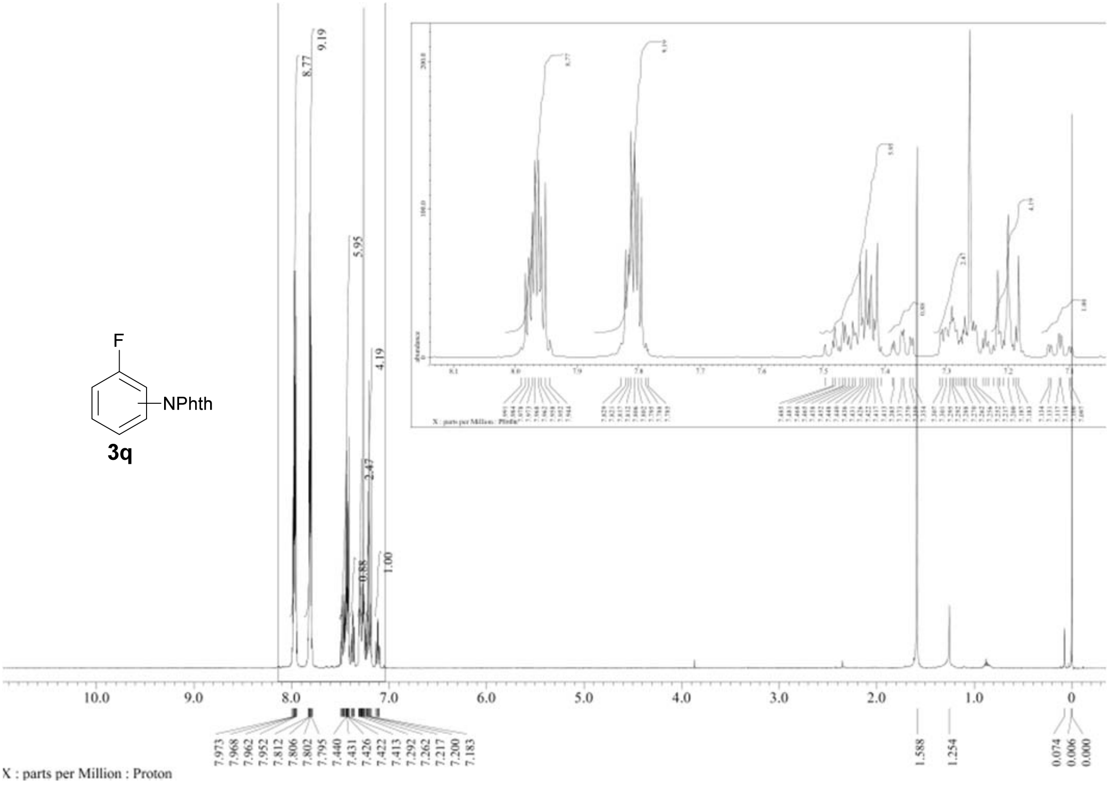
F
NPhth
3q
8.77
9.19
5.95
4.19
2.47
0.88
1.00
10.0
9.0
8.0
7.0
6.0
5.0
4.0
3.0
2.0
1.0
0
7.973
7.968
7.962
7.952
7.812
7.806
7.802
7.795
7.440
7.431
7.426
7.422
7.413
7.292
7.262
7.217
7.200
7.183
1.588
1.254
0.074
0.006
0.000
X : parts per Million : Proton

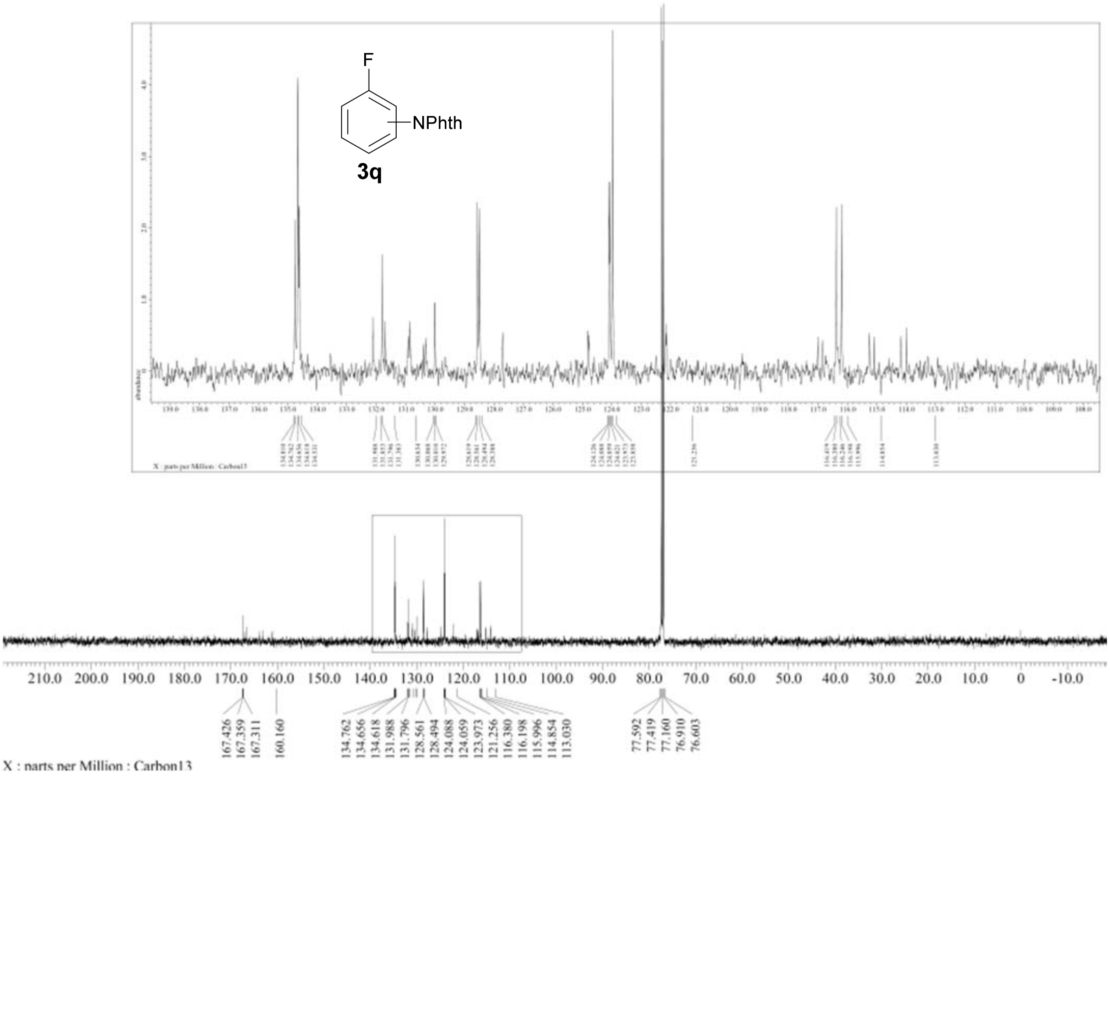
F
NPhth
3q
210.0
200.0
190.0
180.0
170.0
160.0
150.0
140.0
130.0
120.0
110.0
100.0
90.0
80.0
70.0
60.0
50.0
40.0
30.0
20.0
10.0
0
-10.0
167.426
167.359
167.311
160.160
134.762
134.656
134.618
131.988
131.796
128.561
128.494
124.088
124.059
123.973
121.256
116.380
116.198
115.996
114.854
113.030
77.592
77.419
77.160
76.910
76.603
X : parts per Million : Carbon13

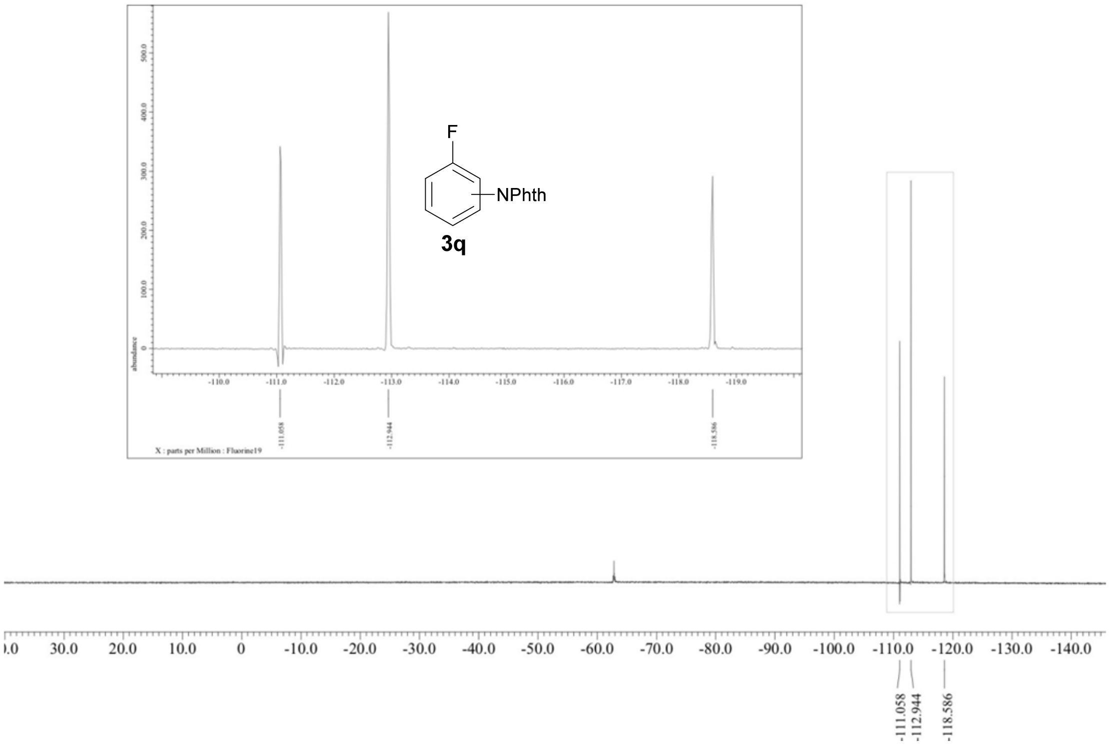
F
NPhth
3q
X : parts per Million : Fluorine19
-111.058
-112.944
-118.586

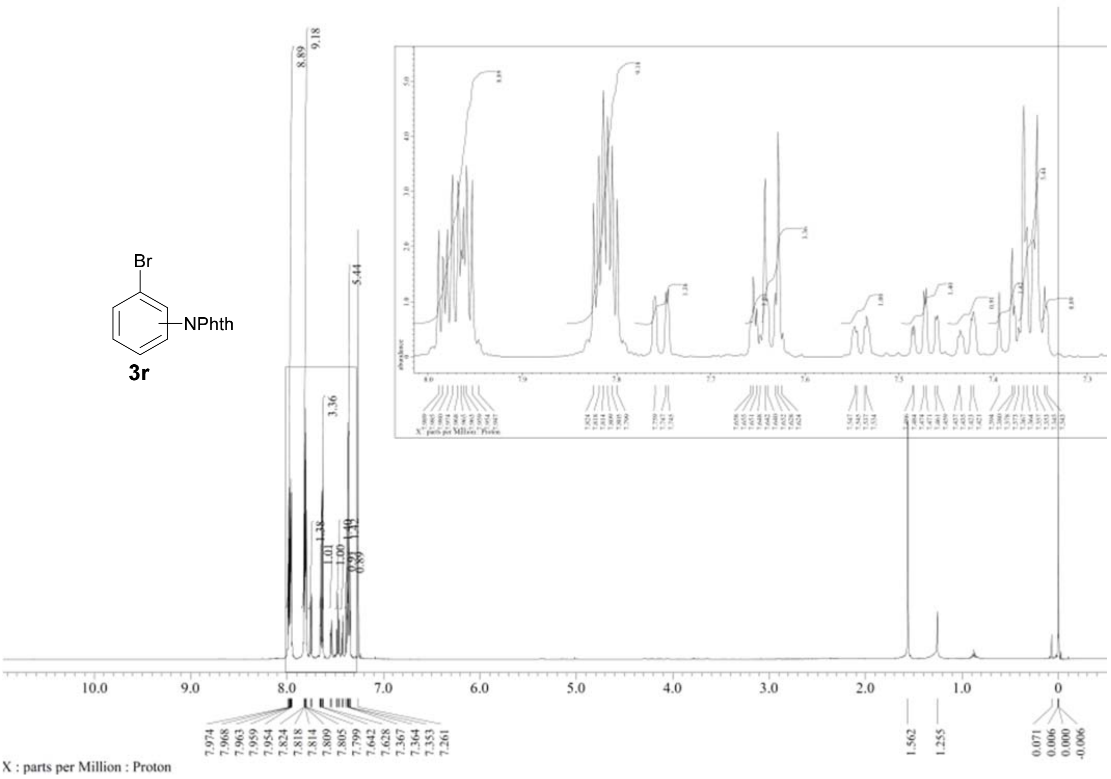
Br
NPhth
3r
8.89
9.18
3.36
5.44
1.38
1.01
1.00
1.40
1.42
0.89
7.974
7.968
7.963
7.959
7.954
7.824
7.818
7.814
7.809
7.805
7.799
7.642
7.628
7.367
7.364
7.353
7.261
1.562
1.255
0.071
0.006
0.000
-0.006
X : parts per Million : Proton

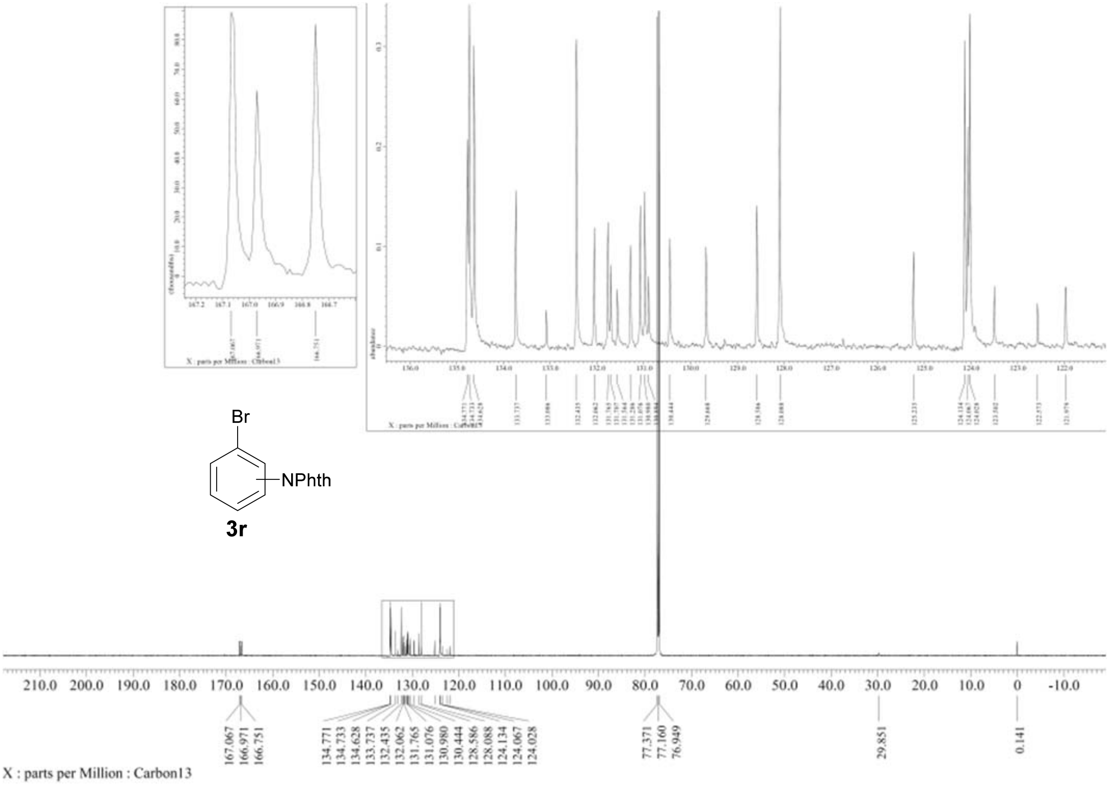
Br
NPhth
3r
167.067
166.971
166.751
134.771
134.733
134.628
133.737
132.435
132.062
131.765
131.076
130.980
130.444
128.586
128.088
124.134
124.067
124.028
77.371
77.160
76.949
29.851
0.141
X : parts per Million : Carbon13

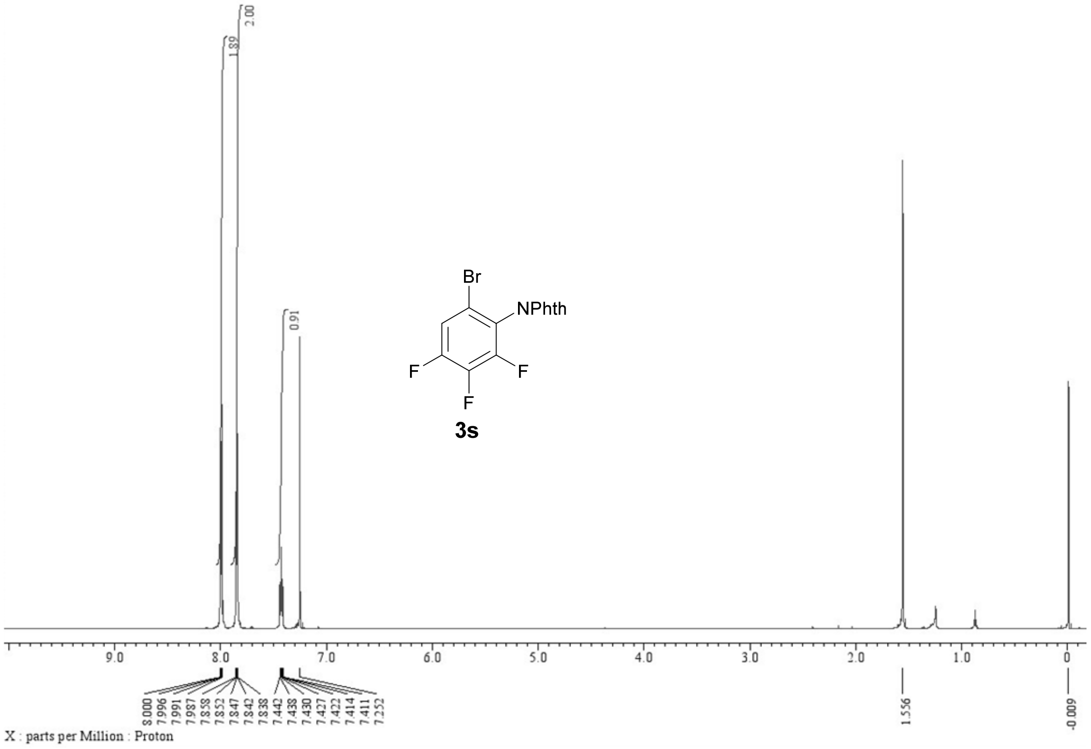
Br
NPhth
F
F
F
3s
1.89
2.00
0.91
8.000
7.996
7.991
7.987
7.858
7.852
7.847
7.842
7.838
7.442
7.438
7.430
7.427
7.422
7.414
7.411
7.252
1.556
-0.009
X : parts per Million : Proton

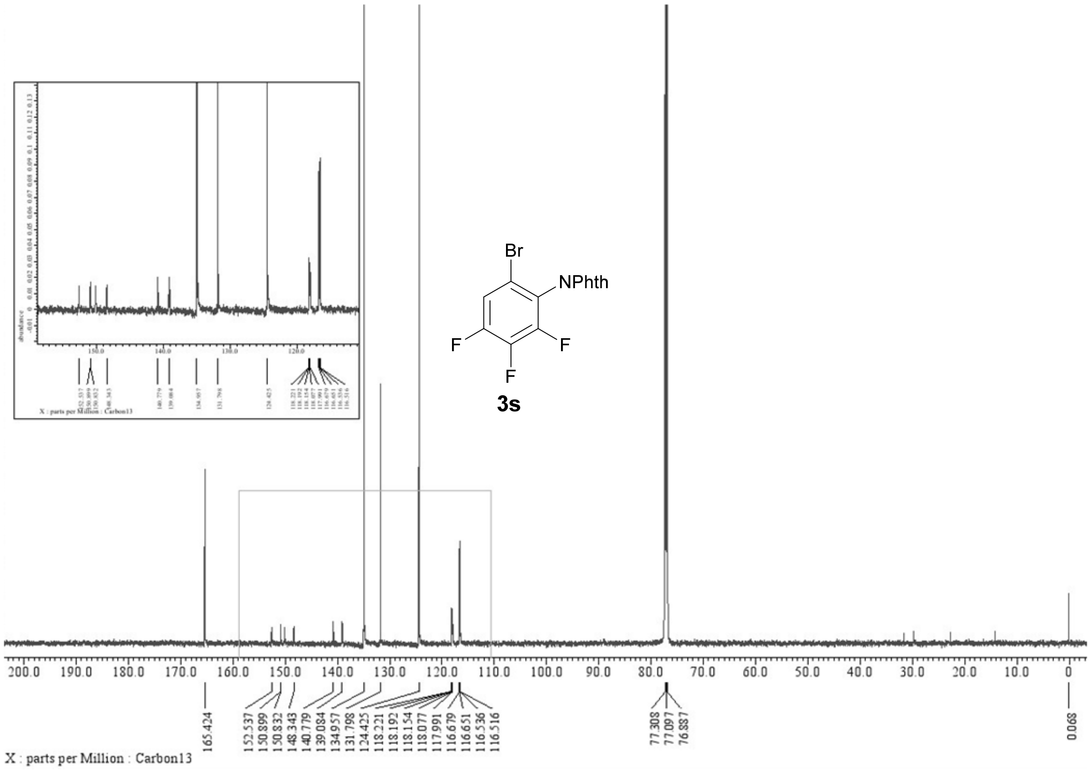
Br
NPhth
F
F
F
3s
165.424
152.537
150.899
150.832
148.343
140.779
139.084
134.957
131.798
124.425
118.221
118.192
118.154
118.077
117.991
116.679
116.651
116.536
116.516
77.308
77.097
76.887
0.068
X : parts per Million : Carbon13

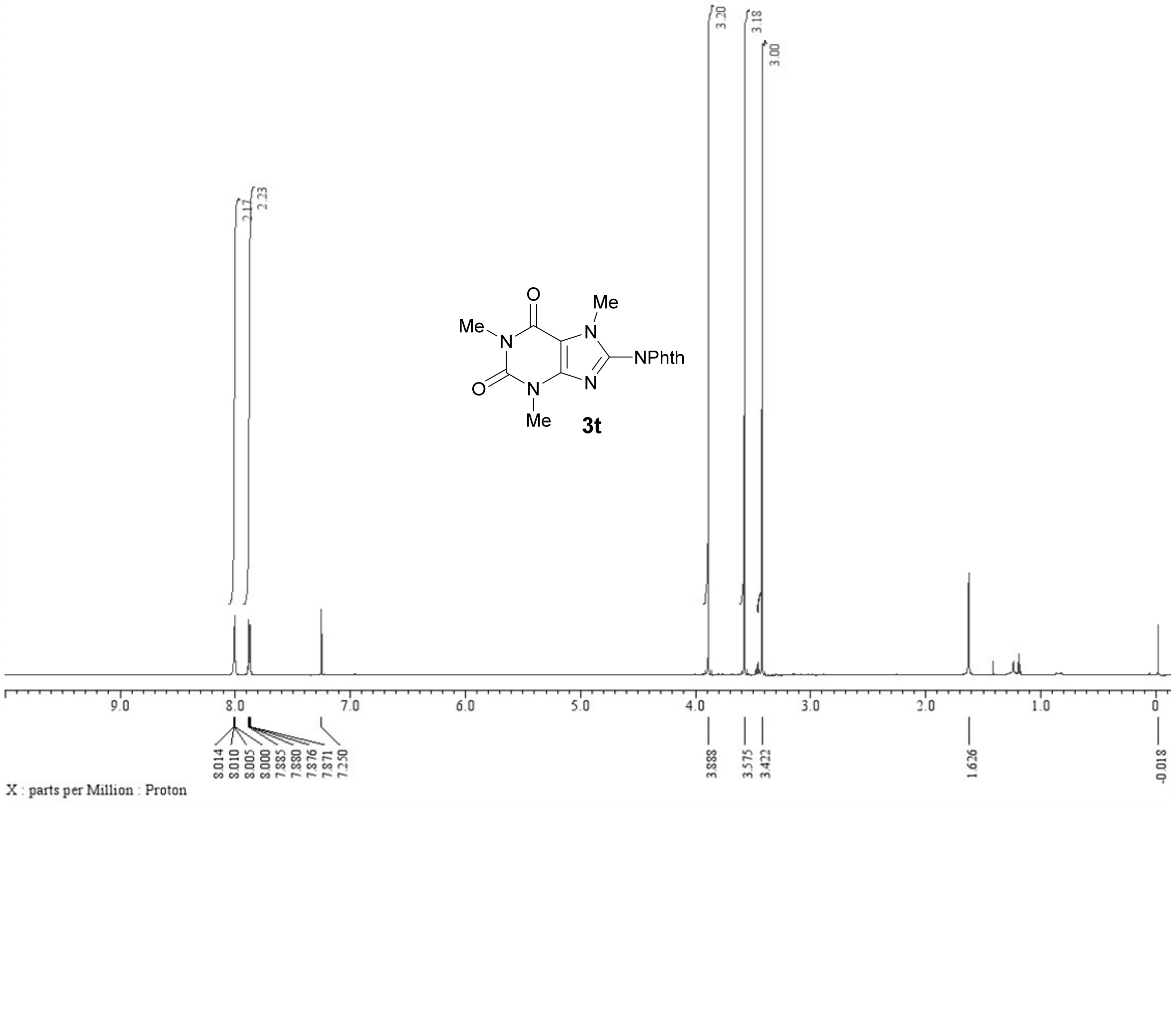
O
Me
Me
N
N
NPhth
O
N
N
Me
3t
3.20
3.18
3.00
2.17
2.23
8.014
8.010
8.005
8.000
7.885
7.880
7.876
7.871
7.250
3.888
3.575
3.422
1.626
-0.018
X : parts per Million : Proton

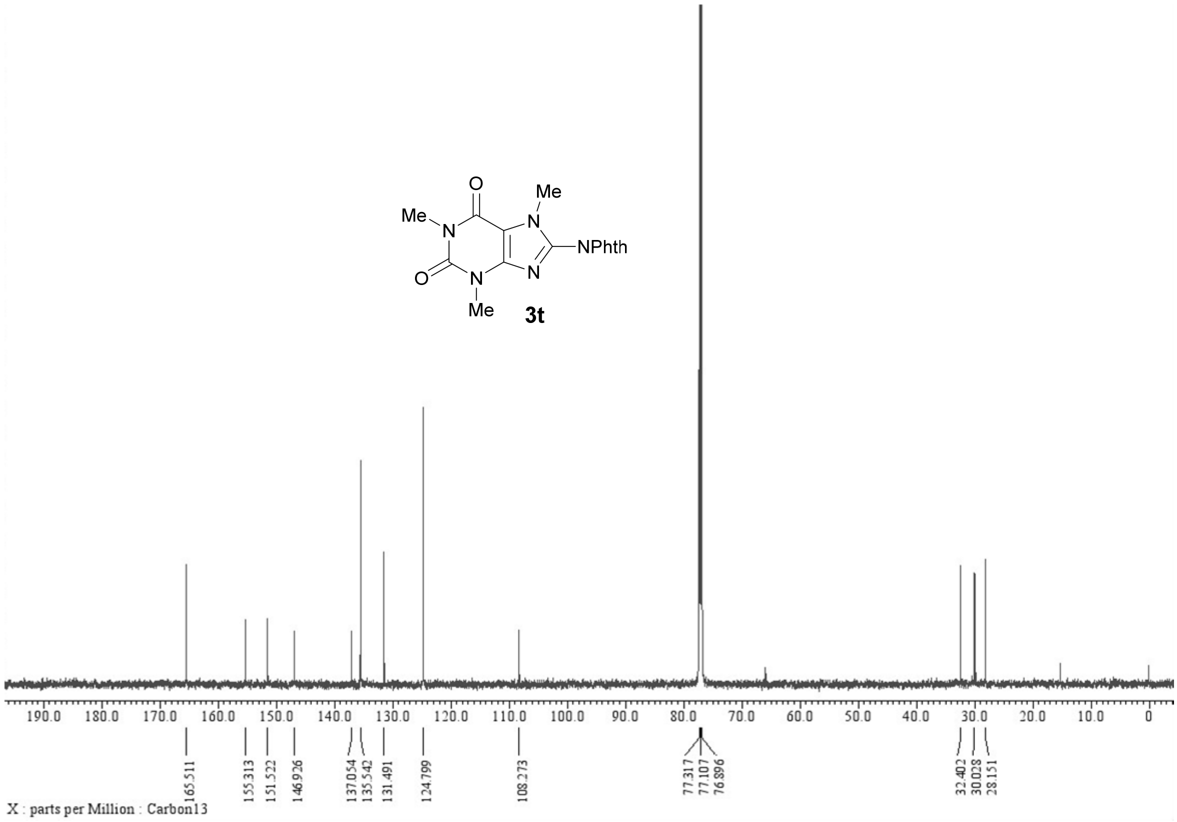

Me
Me
NPhth
Me
3t
X : parts per Million : Carbon13
165.511
155.313
151.522
146.926
137.054
135.542
131.491
124.799
108.273
77.317
77.107
76.896
32.402
30.028
28.151


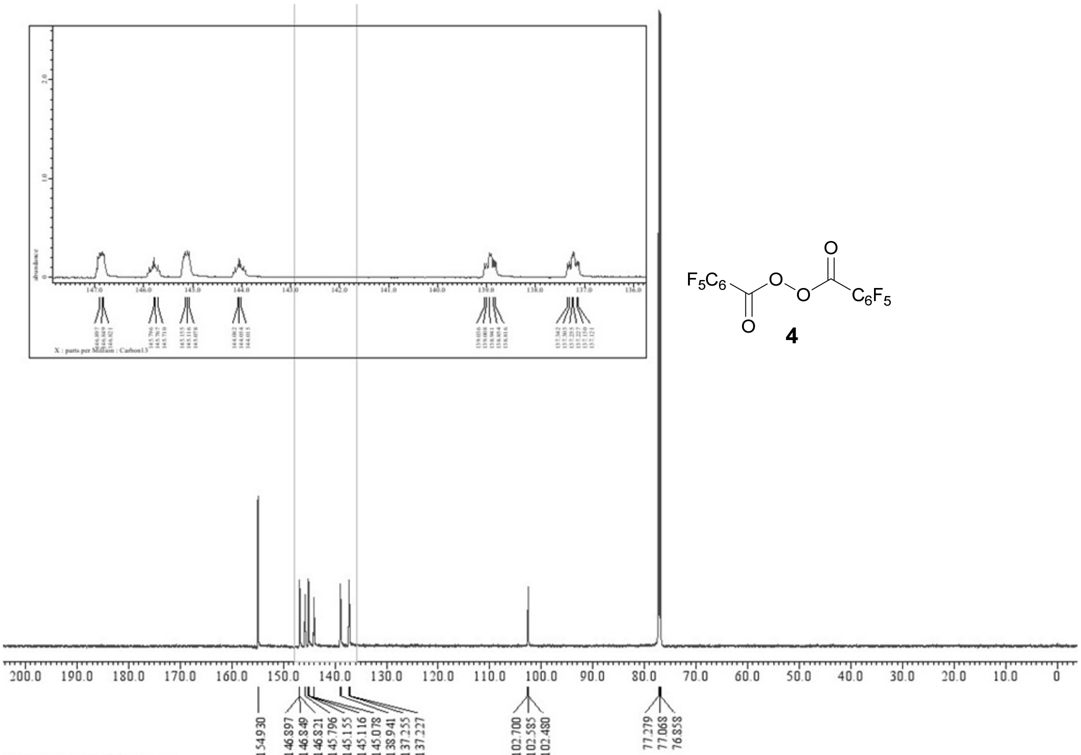

$F_5C_6$
$C_6F_5$
4
154.930
146.897
146.849
146.821
145.796
145.155
145.116
145.078
138.941
137.255
137.227
102.700
102.585
102.480
77.279
77.068
76.858

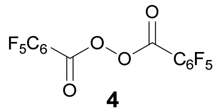
F5C6
O
O
O
O
C6F5

**4**

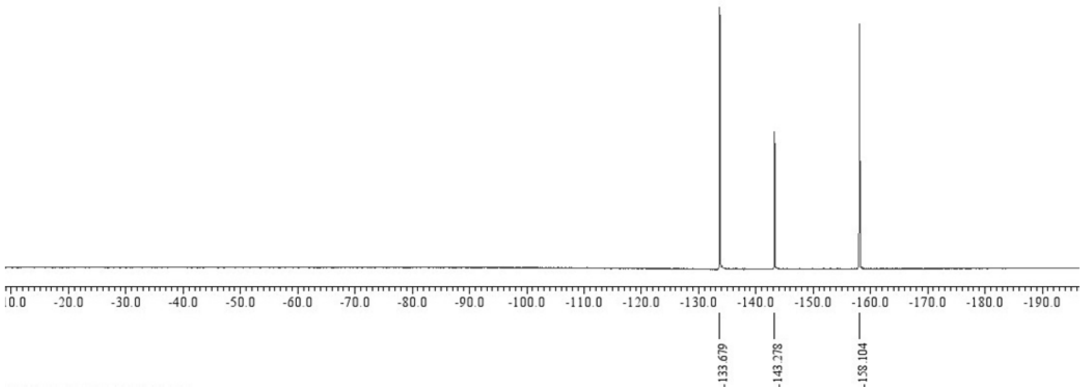
-133.679
-143.278
-158.104

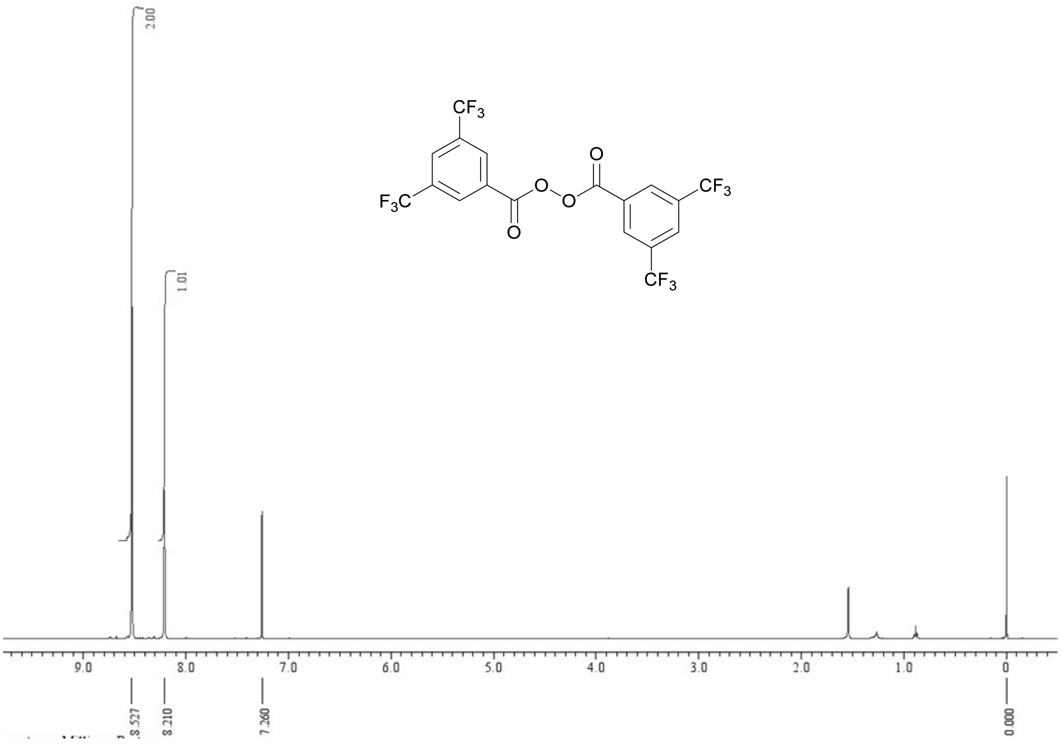
2.00
1.01
8.527
8.210
7.260
0.000

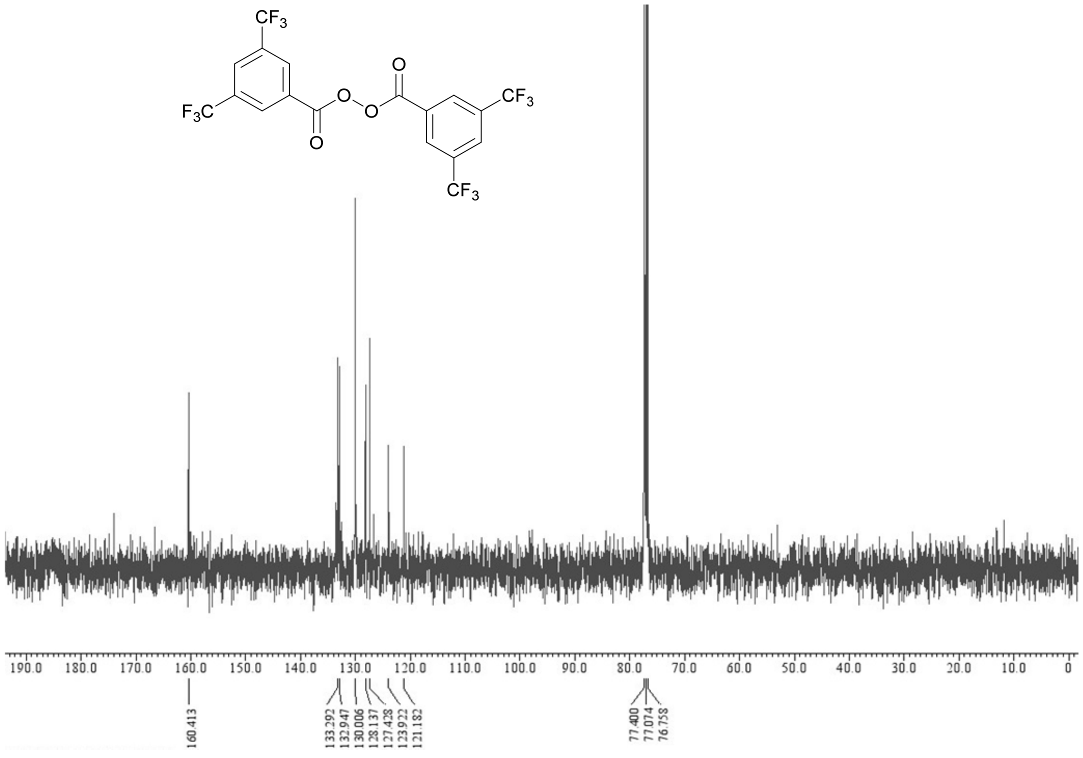
CF3
F3C
O
O
O
O
CF3
CF3
160.413
133.292
132.947
130.006
128.137
127.428
123.922
121.182
77.400
77.074
76.758

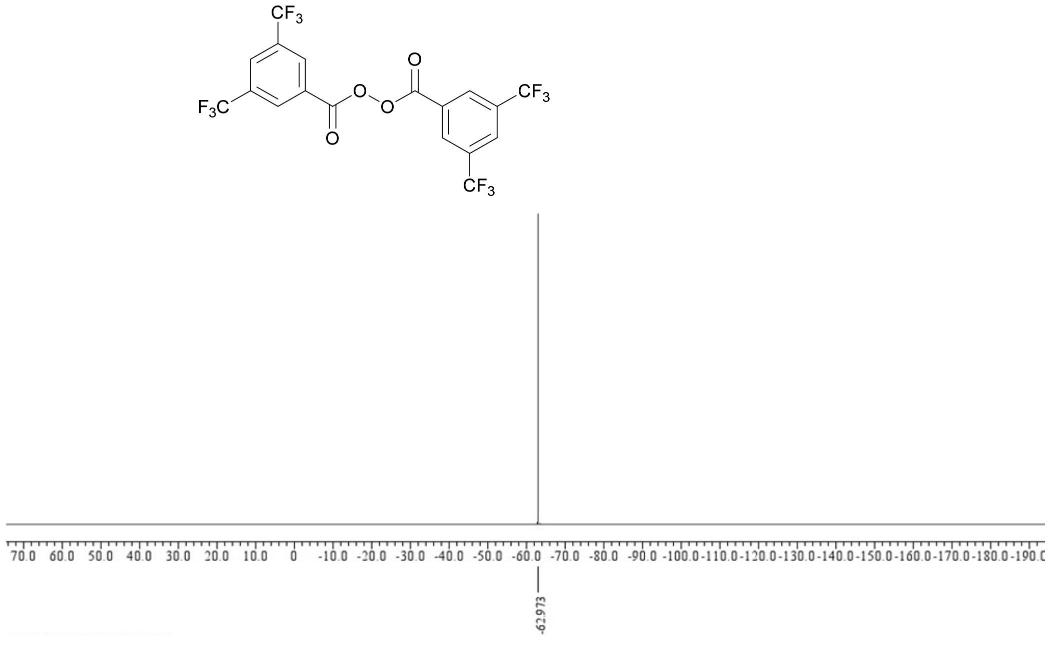
CF3
F3C
O
O
O
O
CF3
CF3
-62.973

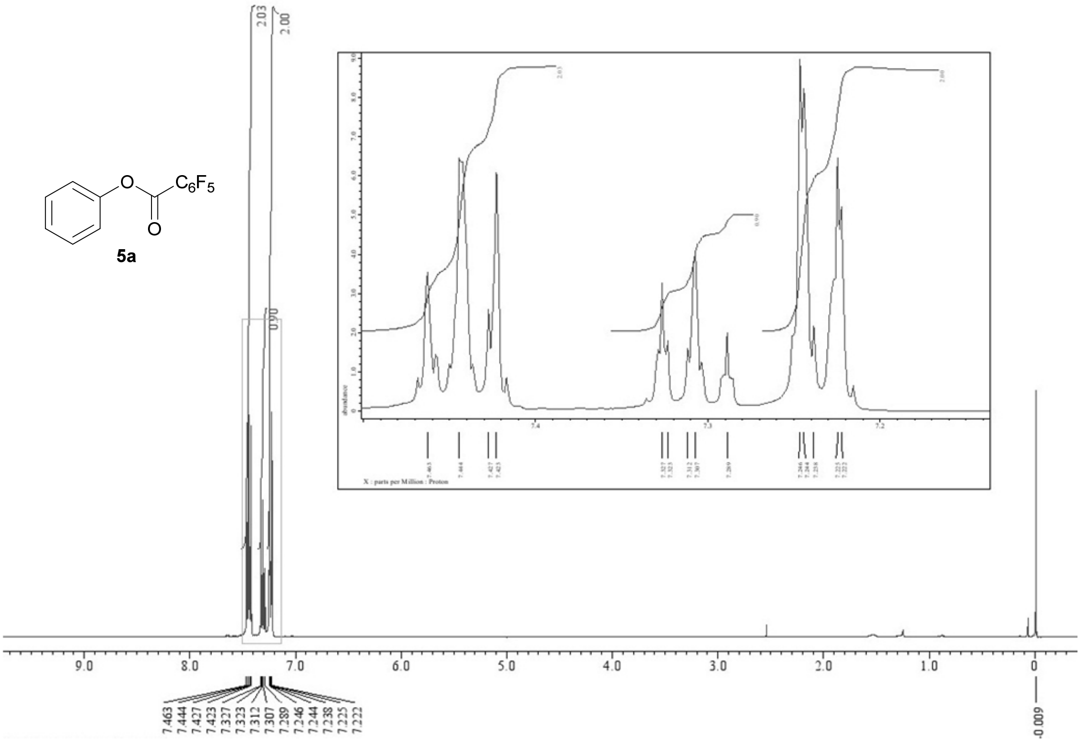
O
C6F5
O
5a
2.03
2.00
0.90
9.0
8.0
7.0
6.0
5.0
4.0
3.0
2.0
1.0
0
7.463
7.444
7.427
7.423
7.327
7.323
7.312
7.307
7.289
7.246
7.244
7.238
7.225
7.222
-0.009

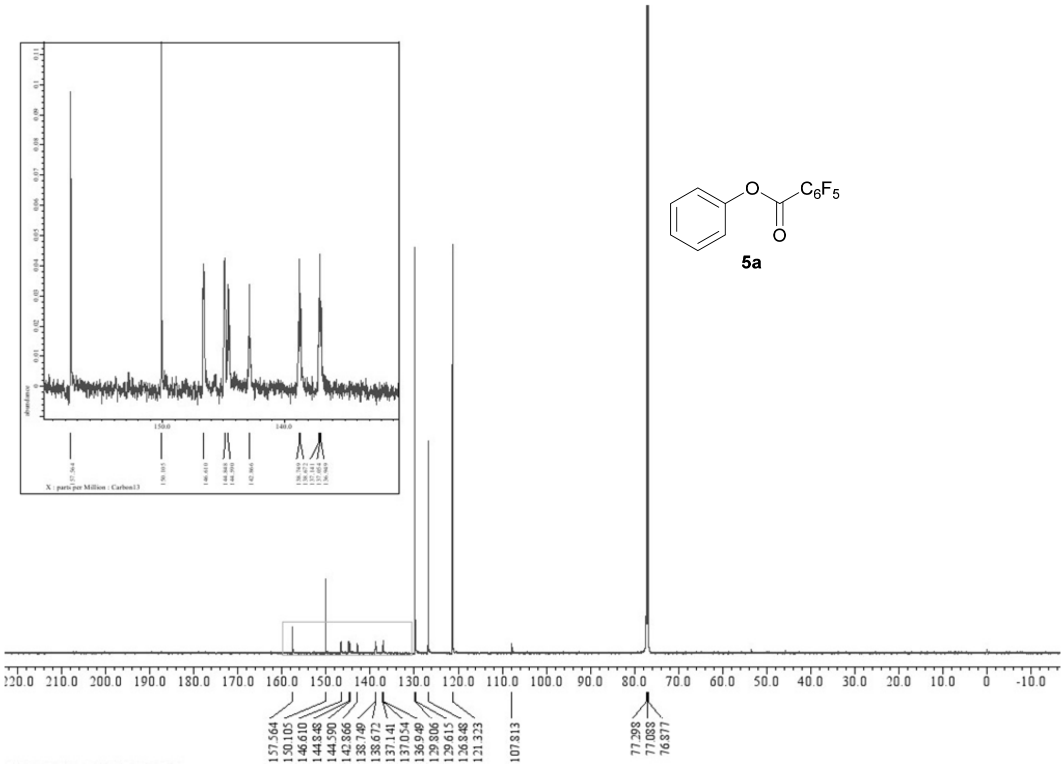
O
C6F5
O
5a
157.564
150.105
146.610
144.848
144.590
142.866
138.749
138.672
137.141
137.054
136.949
129.806
129.615
126.848
121.323
107.813
77.298
77.088
76.877

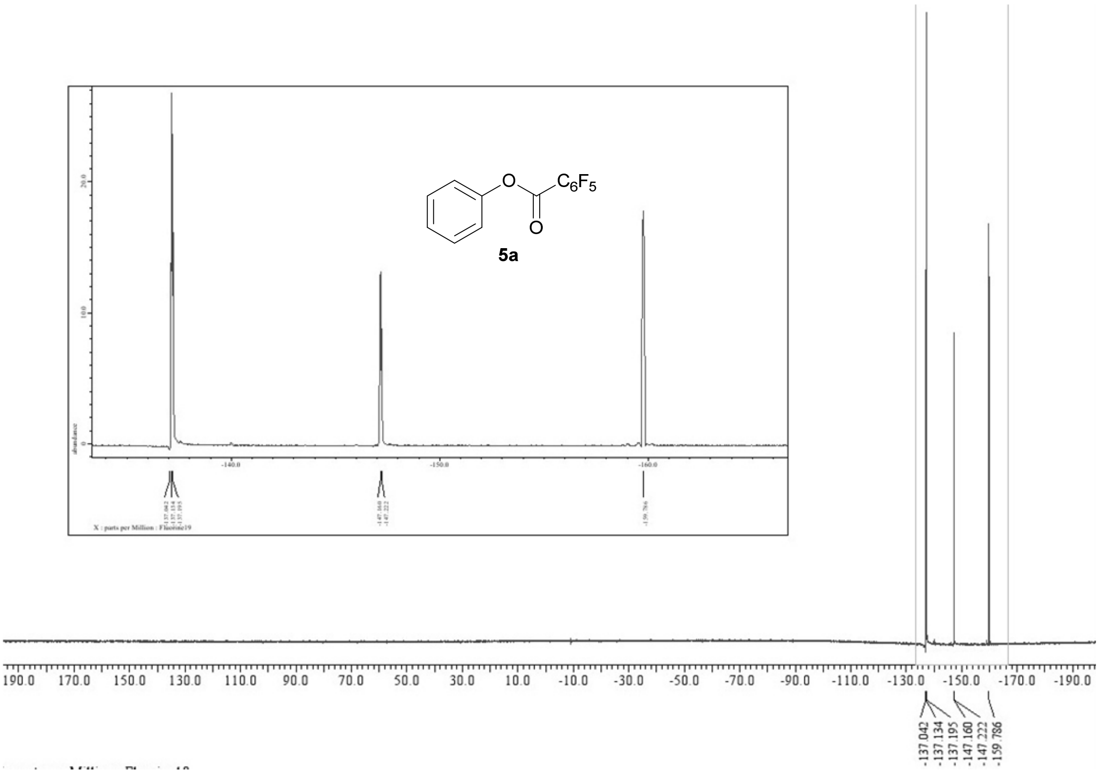

5a
$C_6F_5$
-137.042
-137.134
-137.195
-147.160
-147.222
-159.786


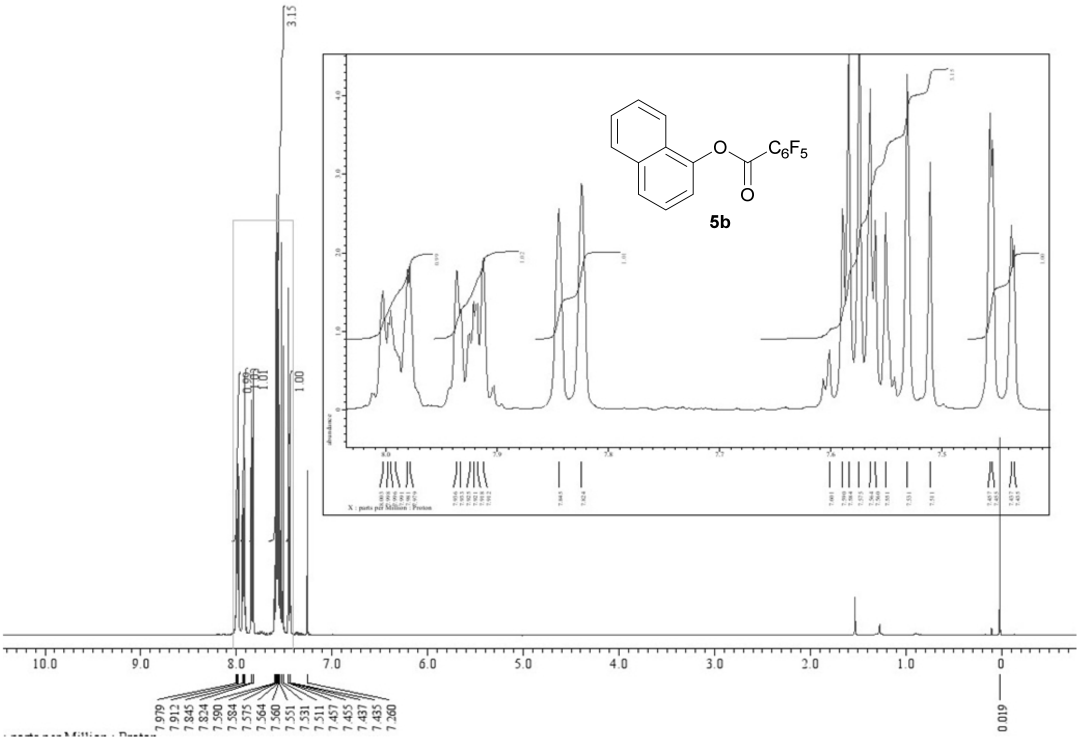

5b
$C_6F_5$
7.979
7.912
7.845
7.824
7.590
7.584
7.575
7.564
7.560
7.551
7.531
7.511
7.457
7.455
7.437
7.435
7.260
0.019

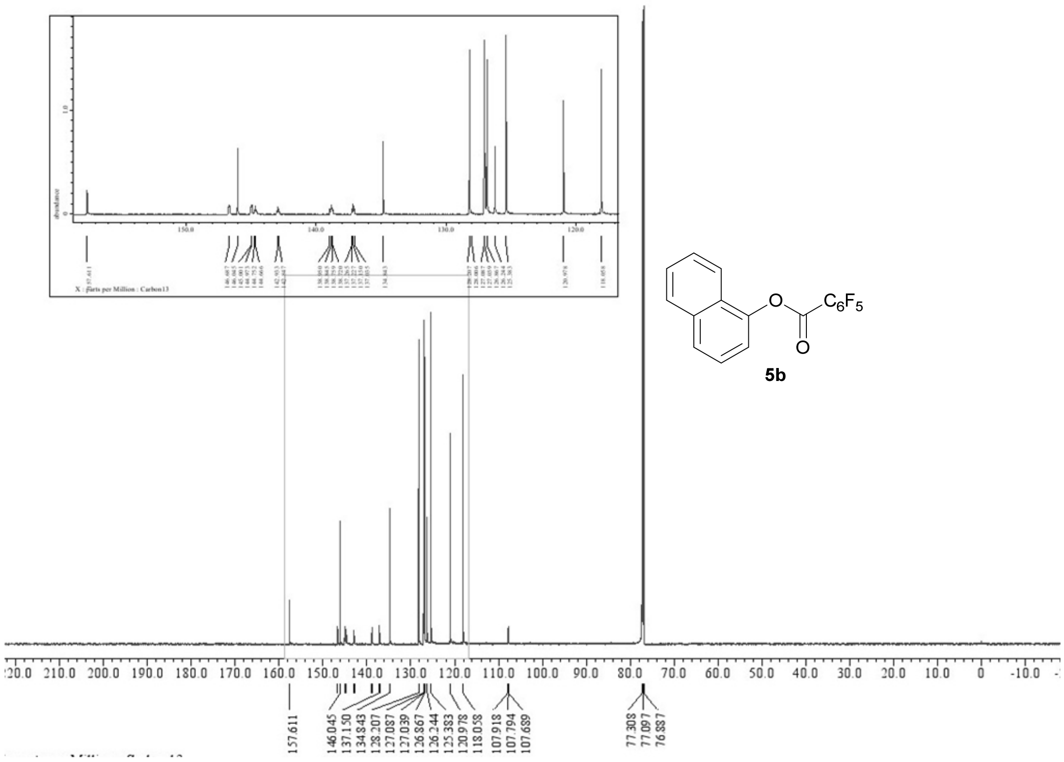
C6F5
O
5b
X : parts per Million : Carbon13
157.611
146.045
137.150
134.843
128.207
127.087
127.039
126.867
126.244
125.383
120.978
118.058
107.918
107.794
107.689
77.308
77.097
76.887

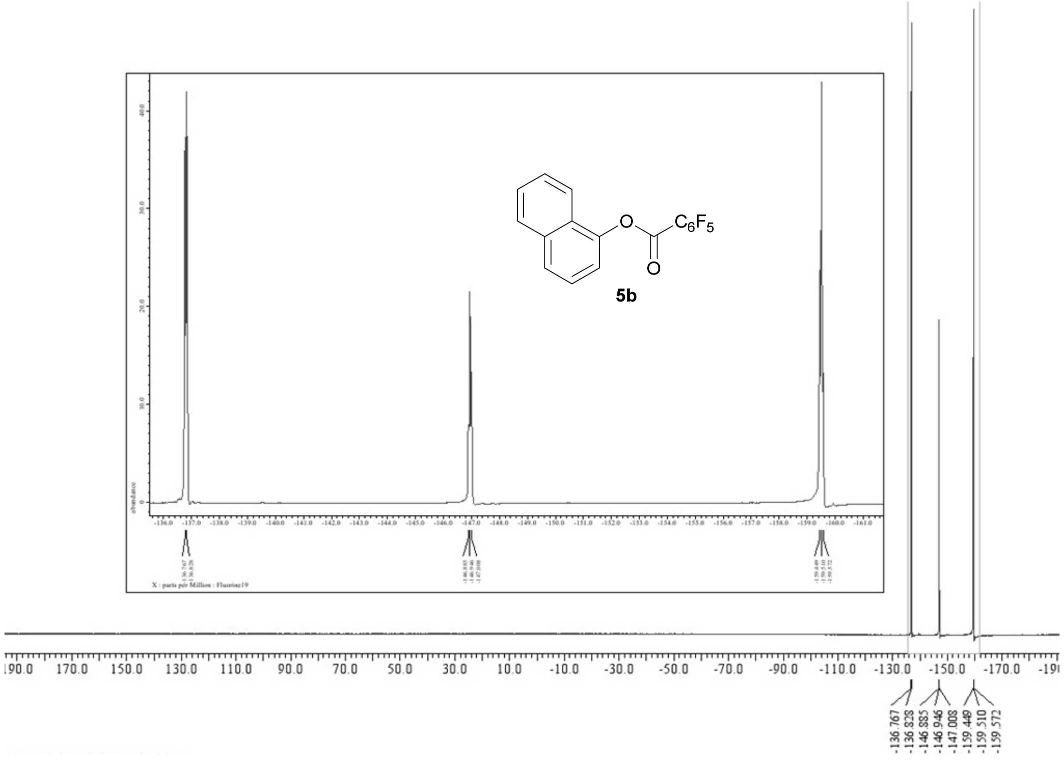
C6F5
O
5b
X : parts per Million : Fluorine19
-136.767
-136.828
-146.885
-146.946
-147.008
-159.449
-159.510
-159.572

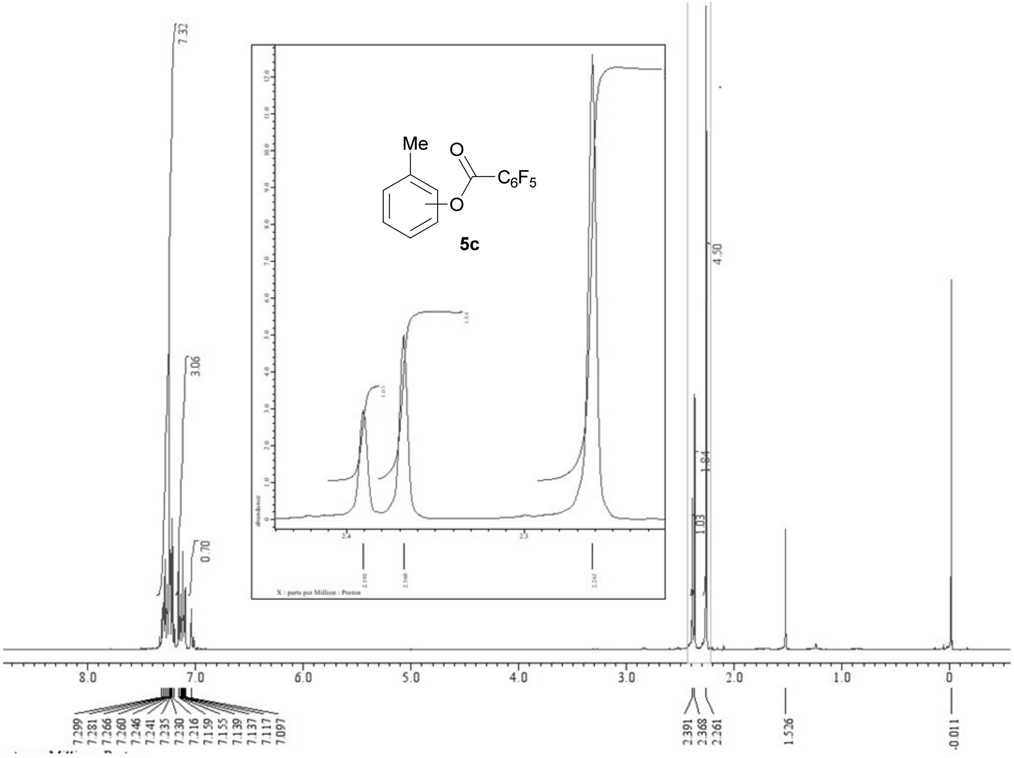
Me
O
O
C6F5
5c
7.32
3.06
0.70
4.50
1.84
1.03
8.0
7.0
6.0
5.0
4.0
3.0
2.0
1.0
0
2.391
2.368
2.261
1.526
-0.011

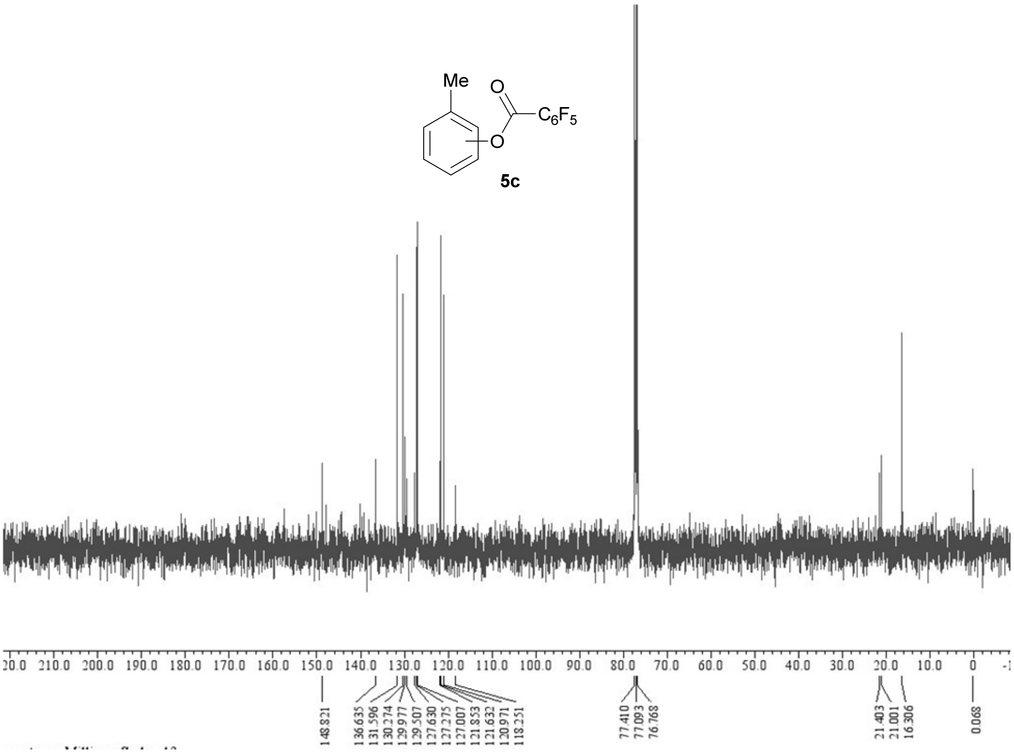
Me
O
O
C6F5
5c
148.821
136.635
131.596
130.274
129.977
129.507
127.630
127.275
127.007
121.853
121.632
120.971
118.251
77.410
77.093
76.768
21.403
21.001
16.306
0.068

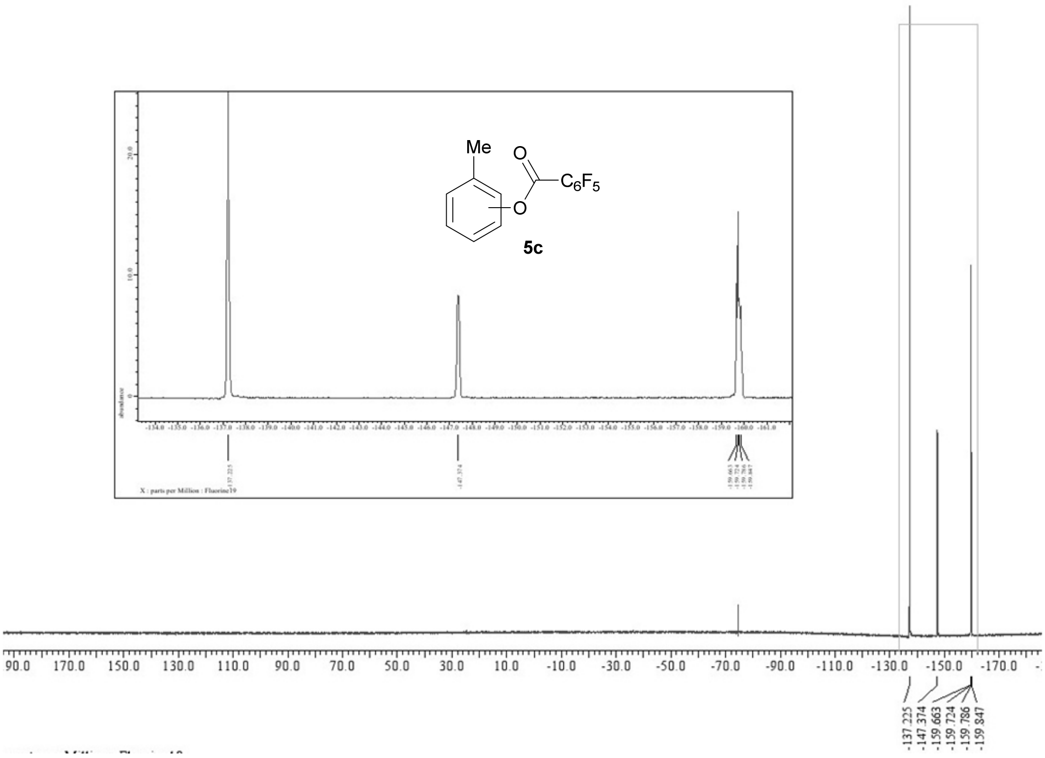
Me
O
$C_6F_5$
O
5c
X : parts per Million : Fluorine19
-137.225
-147.374
-159.663
-159.724
-159.786
-159.847

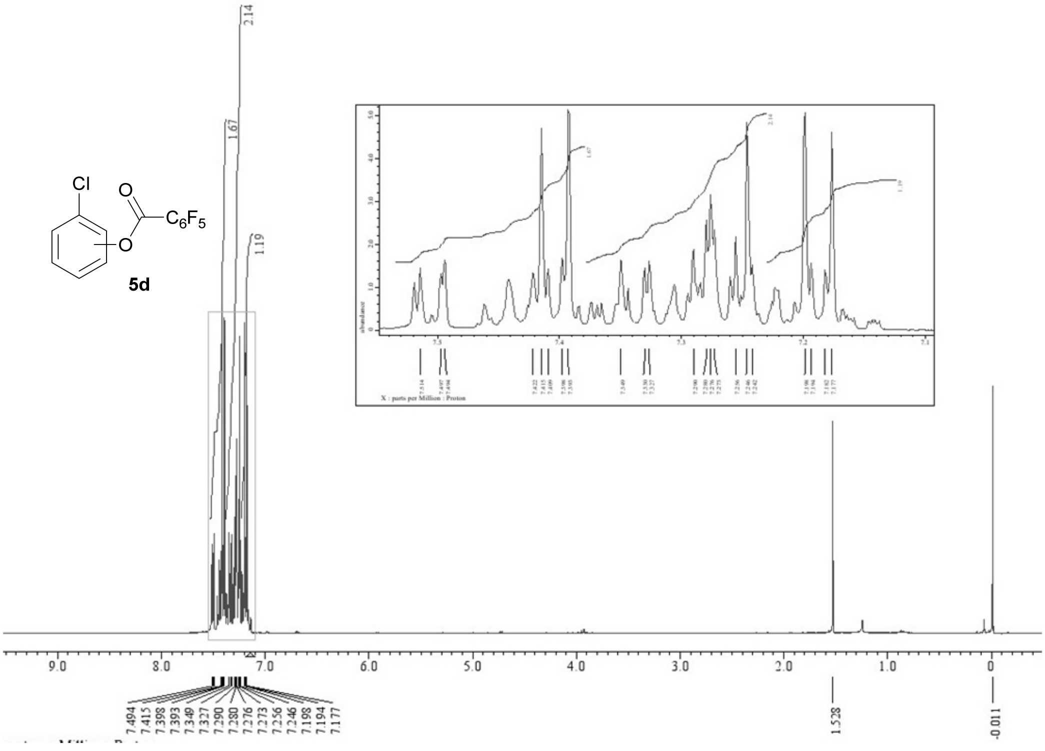
Cl
O
$C_6F_5$
O
5d
2.14
1.67
1.19
X : parts per Million : Proton
7.494
7.415
7.398
7.393
7.349
7.327
7.290
7.280
7.276
7.273
7.256
7.246
7.198
7.194
7.177
1.528
-0.011

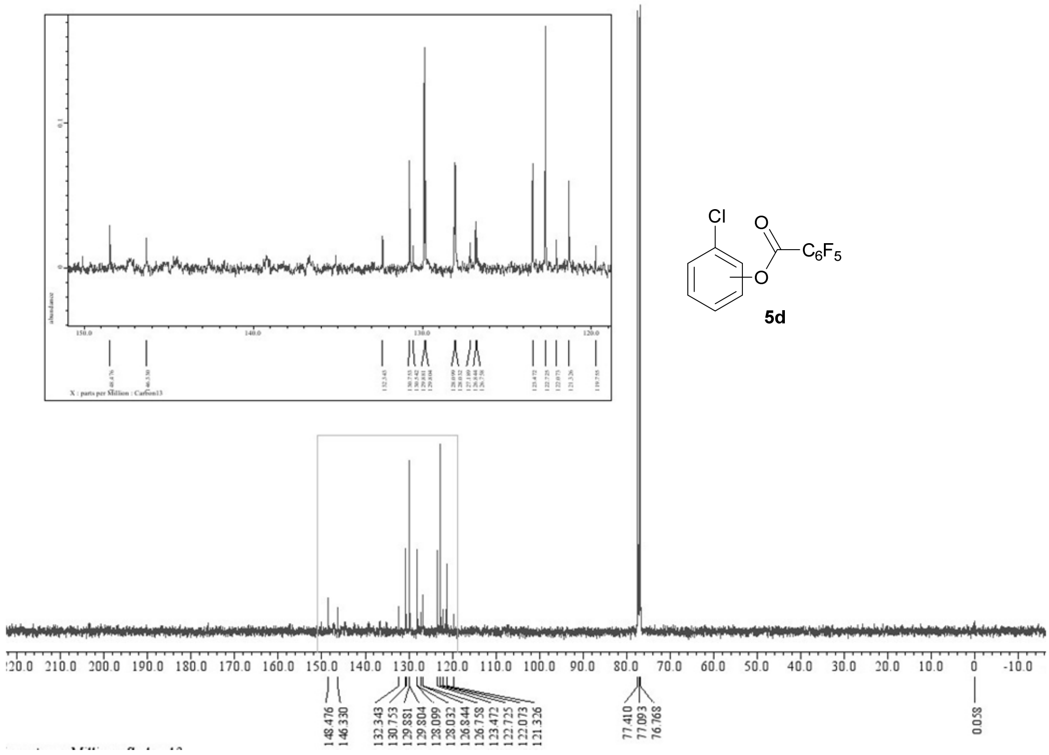
5d
X : parts per Million : Carbon13
148.476
146.330
132.343
130.753
129.881
129.804
128.099
128.032
126.844
126.758
123.472
122.725
122.073
121.326
77.410
77.093
76.768
0.058

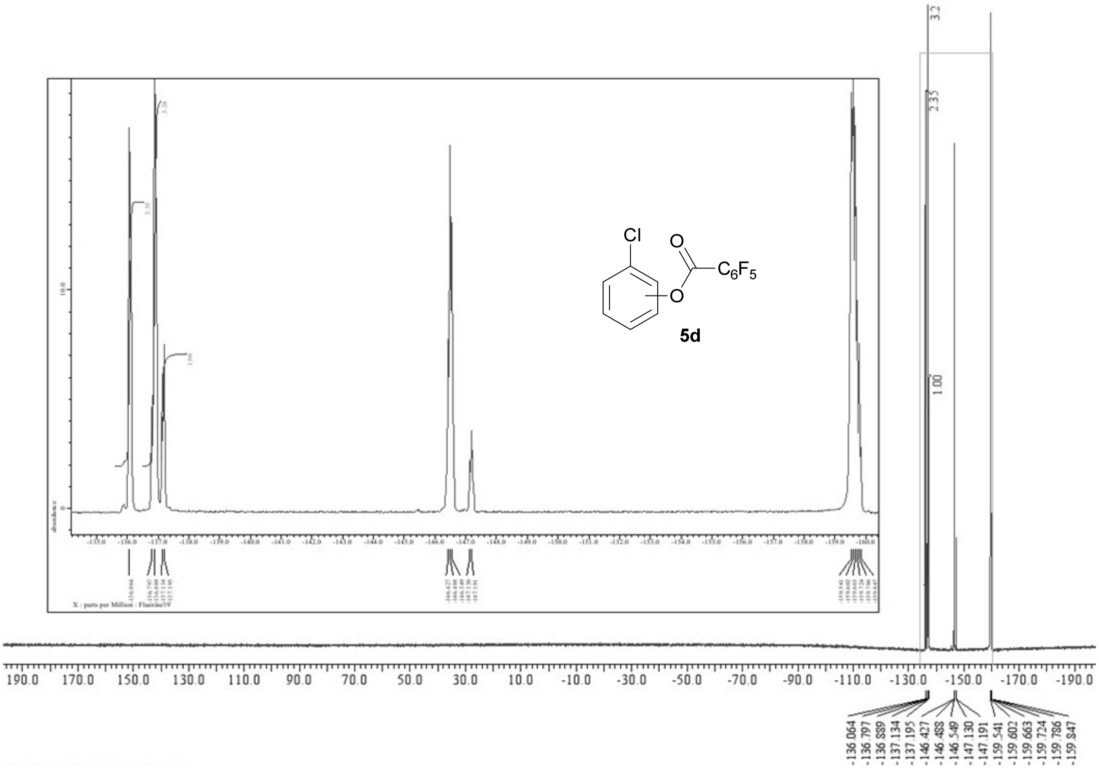
5d
X : parts per Million : Fluorine19
-136.064
-136.797
-136.859
-137.134
-137.195
-146.427
-146.488
-146.549
-147.130
-147.191
-159.541
-159.602
-159.663
-159.724
-159.786
-159.847
